# Introduction of chirality at C1 position of 1-substituted-3,4-dihydroisoquinoline by its enantioselective reduction: synthesis of chiral 1-substituted-1,2,3,4-tetrahydroisoquinoline – a review

**DOI:** 10.1039/d3ra01413d

**Published:** 2023-04-06

**Authors:** Md. Moaz Ahmed Asif, Susmita Roy Lisa, Nazmul Qais

**Affiliations:** a Department of Pharmaceutical Chemistry, Faculty of Pharmacy, University of Dhaka Dhaka 1000 Bangladesh; b Department of Clinical Pharmacy and Pharmacology, Faculty of Pharmacy, University of Dhaka Dhaka 1000 Bangladesh nqais@du.ac.bd

## Abstract

There is a wide range of biological activities associated with C1 chiral carbon containing 1-substituted-1,2,3,4-tetrahydroisoquinolines (1-substituted-THIQs) which constitute the isoquinoline alkaloids, a large group of natural products. This work summarizes several novel catalytic stereoselective approaches to enantioselectively reduce the 1-substituted-3,4-dihydroisoquinolines (1-substituted-DHIQs) to produce the desired 1-substituted-THIQs. The 1-substituted-DHIQs were prepared by using the Bischler–Napieralski reaction. The enantioselective reduction of 1-substituted-DHIQs was accomplished by using chiral hydride reducing agents, by hydrogenation with a chiral catalyst, by enantioselective reduction of DHIQs possessing a chiral auxiliary at the imine nitrogen by achiral metallic hydride reducing agents, or by enzymatic catalysis. Among these methods, much more work was carried out on the hydrogenation of 1-substituted-DHIQs in the presence of a chiral catalyst. This review summarizes articles and advancements on this topic from 1972 to 2023.

## Abstract 2

The chirality introduced at the C1 position of 1-substituted-1,2,3,4-tetrahydroisoquinolines, obtained by four methods of enantioselective reduction of 1-substituted-3,4-dihydroisoquinolines, are vital for various biological activities.

## Graphical abstract

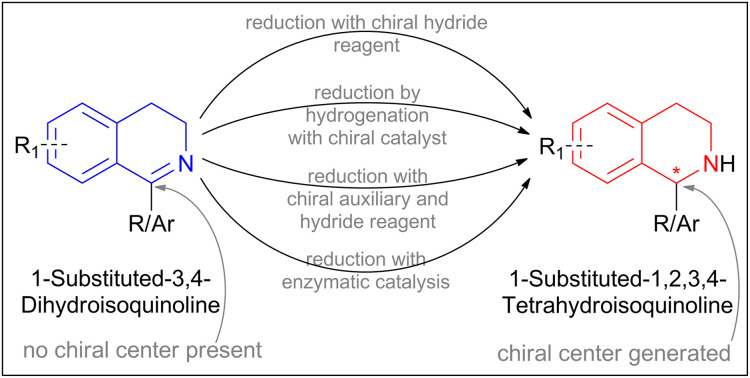

## 1 Introduction

The isoquinoline alkaloids occur primarily in nature and demonstrate a wide range of biological activity and structural diversity. Several effective synthetic techniques have been developed extensively over the last few decades to synthesize these alkaloids in their chiral form with a high enantiomeric excess (% ee). Most of them utilize the diastereoselective or enantioselective catalysis methods for production. These alkaloids have constantly been prominent candidates for organic synthesis because of their structure, bioactivities, and prospective as intriguing intellectual challenges as well as potential therapeutic compounds.^1–5^

**Fig. 1:**
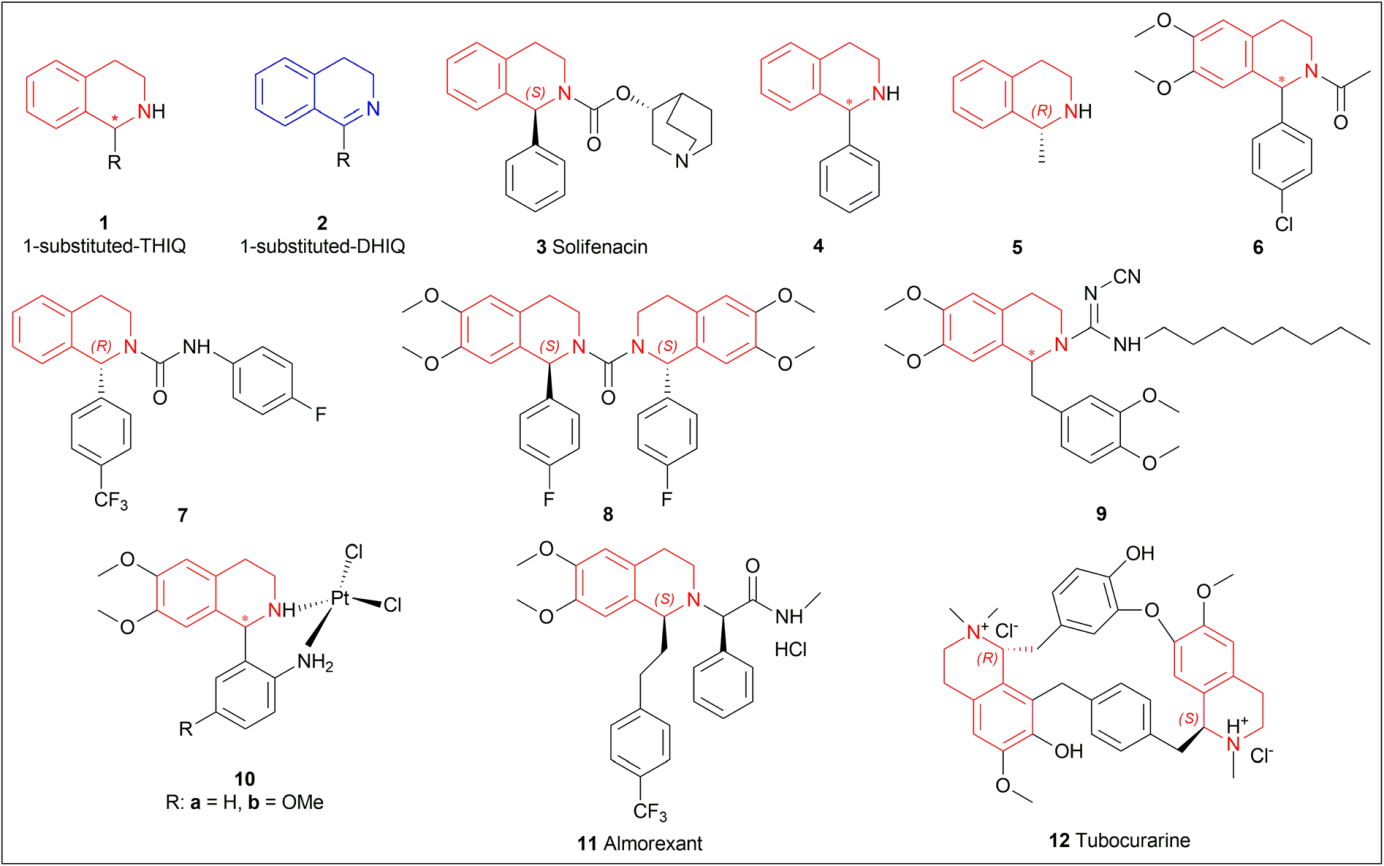
Structures of compounds having 1-substituted-THIQs.

The following examples show that the 1-substituted-THIQs 1, reduction result of 1-substituted-DHIQs 2, possess various biological activities such as solifenacin 3 against overactive bladders,^6^4 shows ataxia,^7^5 against 1-benzyl-THIQ and THIQ-induced Parkinson's disease symptoms,^8,9^6 as a potential noncompetent antagonist of 2-amino-3-(3-hydroxy-5-methylisoxazol-4-yl) propionic acid (AMPA) receptor,^10,11^7 and 8 as transient receptor potential cation channel subfamily M (melastatin) member 8 (TRPM8) channel receptor antagonist,^12,13^9 shows multidrug resistance reversal in cancer,^14^10a and 10b as 2 times more cytotoxic than *cis*-diaminedichloroplatinum(ii) complex against L1210 murine leukemia cells,^15^ almorexant 11 in sleep disorders,^16^ tubocurarine 12 in the South American arrow poisons^17^ (Fig. 1).

**Fig. 2:**
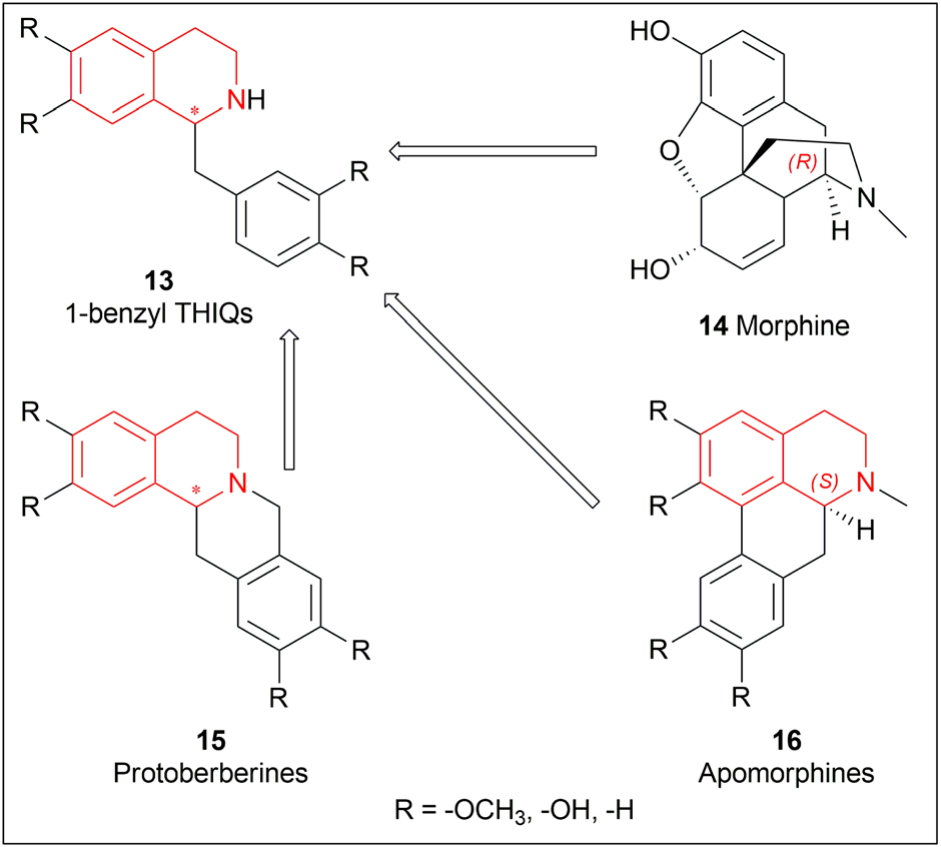
Generation of complex isoquinoline alkaloids from 1-substituted-THIQs.

1-substituted-THIQs 1 are also important for the synthesis of much more complex isoquinoline alkaloids, for example, 1-benzyl-THIQs 13 are necessary precursors for biologically important alkaloids *e.g.*, morphinans 14, protoberberines 15, and apomorphines 16 (Fig. 2).^18^

**Scheme 1:**
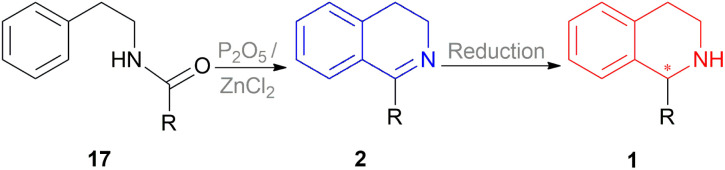
Bischler–Napieralski cyclization.

A vast majority of asymmetric natural and synthetic 1-substituted-THIQ alkaloids show their asymmetricity due to the occurrence of an asymmetric center at the C1 carbon. Hence developing the methodologies to obtain these compounds stereo selectively and access their center for configurational integrity has already been the area of focus. In the past, the classical Bischler–Napieralski reaction has been the topmost recurrently used approach for the synthesis of prochiral 1-substituted-DHIQs 2 (Scheme 1) in which *N*-(2-phenylethyl)acyl amides 17 was cyclized with P_2_O_5_ or dehydrated ZnCl_2_.^19^ The subsequent reduction of 2 can result in 1 in which a chiral carbon is generated at the C-1 position (Scheme 1). Therefore, the enantioselective reduction of 2 may provide optically active 1-substituted-THIQs 1.

The United States Food and Drug Administration mandated in 1992 that information about the biological activity and toxicity of the two enantiomers of each listed racemic medicine must be supplied.^20^ All the early registered racemic medicines also have to be substituted with the appropriate chiral enantiomer under the 1997 complementary regulation.^21^ For this reason, a chiral molecule's enantioselective production of both enantiomers is, therefore, crucial. So, developing an asymmetric synthesis method of 1-substituted-THIQs 1 is highly desirable. The enantioselective reduction of 2 (Scheme 1) can be one for such synthesis. Therefore, we have attempted to discuss here the recent strategies of enantioselective reduction of the 1-substituted-DHIQs 2 to produce the intended 1-substituted-THIQs 1. Besides the conventional methodologies mentioned, this review also includes updates from 1972 to 2021 in this discipline. Various electronic databases such as SciFinder^n^, Google Scholar, and Google Patents were searched widely to obtain information about the synthesis of 1-substituted-THIQs 1.

## 2 Enantioselective reductions of 1-substituted-dihydroisoquinolines (1-substituted-DHIQ)

The enantioselective reduction of the DHIQs 2 may be carried out by the following four methods:

(1) Enantioselective reduction of DHIQs by chiral hydride reducing agents.

(2) Enantioselective reduction of DHIQs by hydrogenation with the help of a chiral catalyst.

(3) Enantioselective reduction of DHIQs possessing chiral auxiliary at the imine nitrogen by achiral metallic hydride reducing agents.

(4) Enantioselective reduction of DHIQs by enzymatic catalysis.

### 2.1 Examples of enantioselective reduction of 1-substituted-DHIQs by chiral hydride reducing agents

#### 2.1.1 Asymmetric reduction with chiral sodium triacyloxyborohydrides

**Scheme 2:**
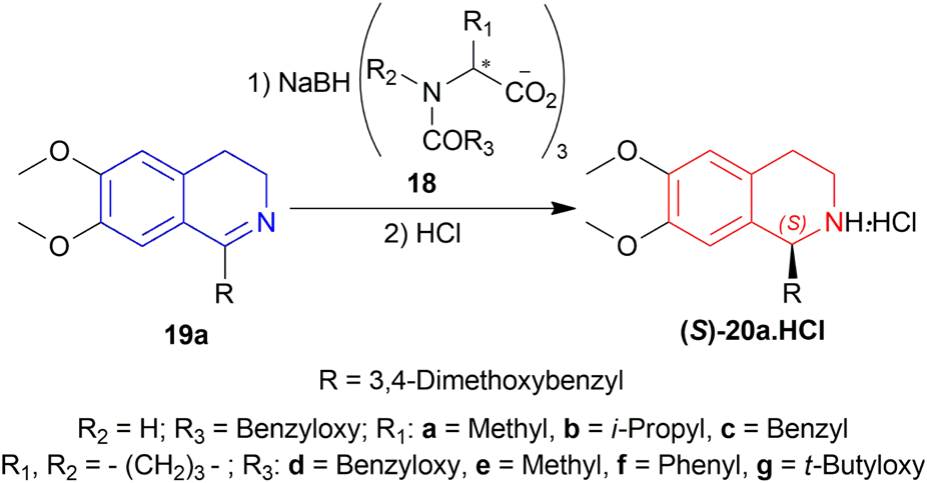
Screening of chiral sodium triacyloxyborohydrides.

Yamada *et al.* screened chiral sodium triacyloxyborohydrides 18a–18g in THF for the asymmetric reduction of 3,4-dihydropapaverine 19a.^22^18d–18g were highly successful in producing (*S*)-norlaudanosine·HCl (*S*)-20a·HCl in high ee of 55–60% with 57–72% chemical yield (Scheme 2).

Among the sodium triacyloxyborohydrides, 18d, prepared from *N*-benzyloxycarbonyl-l-proline and NaBH_4_, was chosen to examine different solvent systems among which, dichloromethane (DCM) and 1,1-dichloroethane produced high chemical yields of 70%, 79% and ee of 71%, 70% respectively. For later experiments, DCM was chosen as the solvent.

**Scheme 3:**
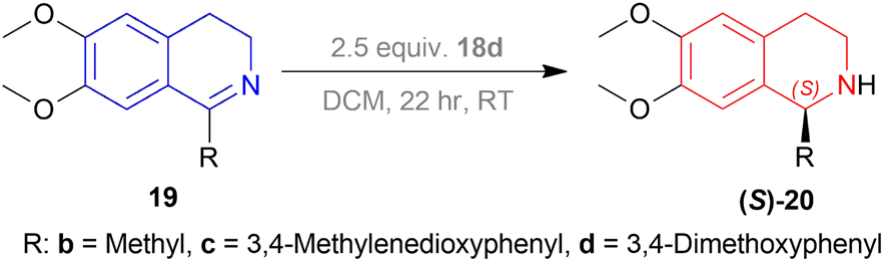
Reduction into (*S*)-salsolidine, (*S*)-norcryptostyline I, and (*S*)-norcryptostyline II.

Then 1-substituted-DHIQs 19b–19d were reduced with 2.5 equiv. of 18d in DCM for 22 h to produce 1-substituted-THIQs (*S*)-salsolidine (*S*)-20b, (*S*)-norcryptostyline I (*S*)-20c, (*S*)-norcryptostyline II (*S*)-20d in high chemical yield of 85%, 90%, 87% and ee of 70%, 86%, 73% respectively (Scheme 3).

So, 18d gave (*S*)-configured products.

#### 2.1.2 Asymmetric chemical reduction of 1-benzyl DHIQs

**Scheme 4:**
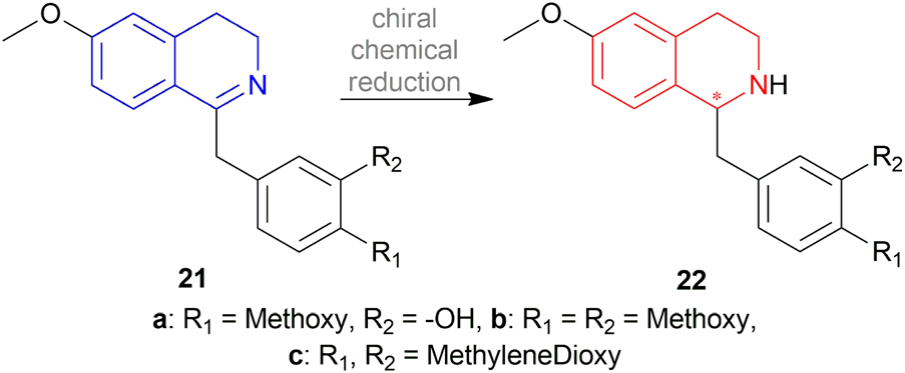
Asymmetric chemical reduction of 1-benzyl-DHIQs.

To synthesize natural and synthetic morphinan compounds, 1-benzyl-1,2,3,4-THIQs are highly useful. For this purpose, rice reduced 1-benzyl-6-methoxy-1,2,3,4-DHIQs 21a–21c to 1-benzyl-6-methoxy-1,2,3,4-THIQs 22a–22c through asymmetric chemical reduction (Scheme 4).^23^

**Fig. 3:**
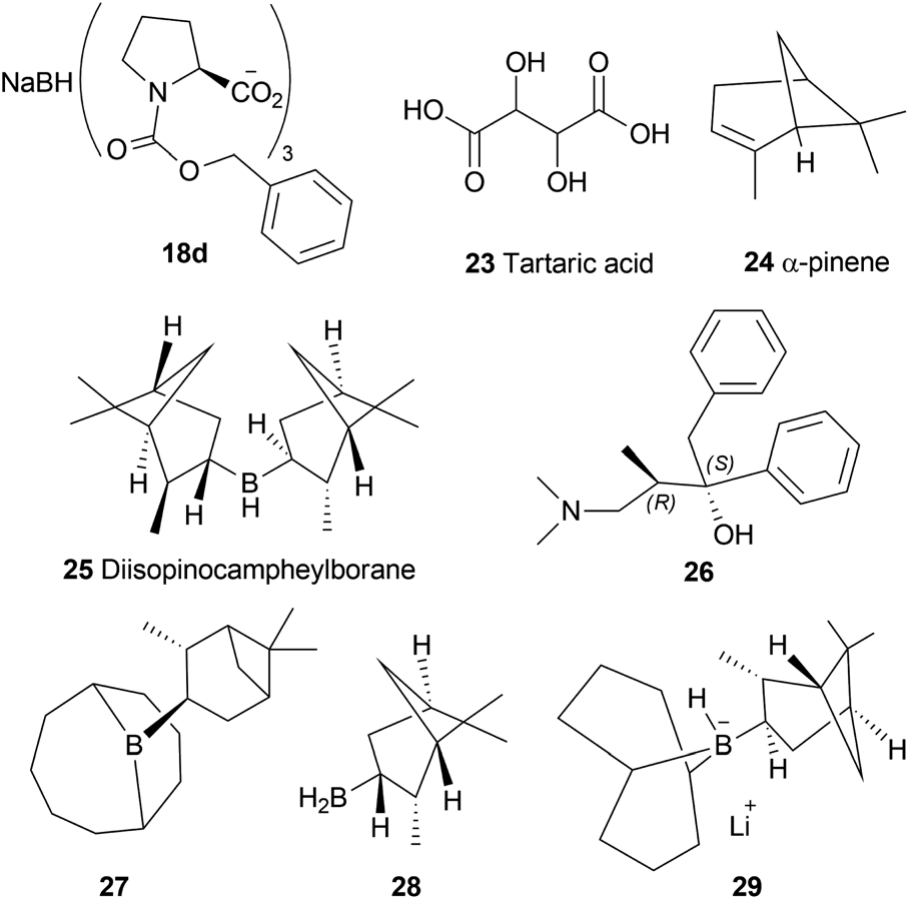
Chiral hydride reducing agents for the reduction of 1-benzyl-DHIQs.

The chiral synthesis of 22a–22c from 21a–21c by asymmetric chemical reduction can be done by many chiral reducing agents. The first one is 18d. With this reducing agent, 85–90% ee was achieved which can be increased by several crystallization. By reacting NaBH_4_ with (+) or (−) tartaric acid 23, other similar chiral reducing agents can be made (Fig. 3).

A 2 : 1 mixture of α-pinene 24 and BH_3_ can produce another reducing agent, diisopinocampheylborane 25. For this agent, 75% chemical yield and 85% ee was observed. (2*S*,3*R*)-4-dimethylamino-3-methyl-1,2-diphenyl-2-butanol or darvon alcohol 26, β-3-pinanyl-9-borabicyclo [3.3.1]-nonane 27, monoisopinocampheylborane 28, and lithium β-isopinocampheyl-9-borabicyclo[3.3.1]-nonyl hydride 29 can also be used (Fig. 3).

The advantages of this process are:

(1). The desired product or isomer has >85–100% ee.

(2). The racemization of the undesirable product and re-resolution is not needed.

#### 2.1.3 Reduction using K-glucoride, Itsuno's reagent, Mosher's reagent

**Fig. 4:**
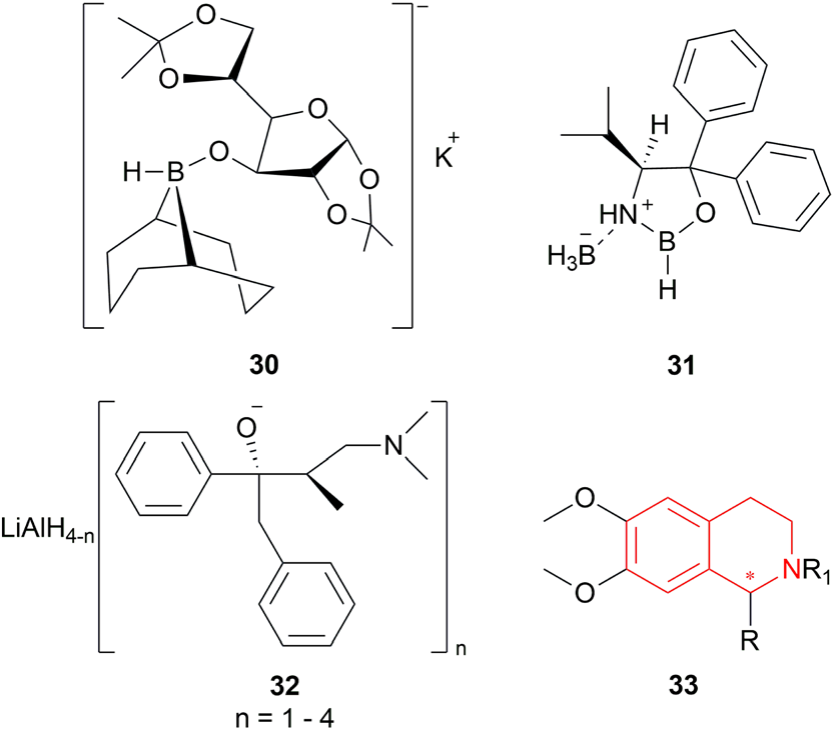
K-glucoride, Itsuno's reagent, and Mosher's reagent to get 1-substituted-6,7-dimethoxy-THIQs.

**Scheme 5:**
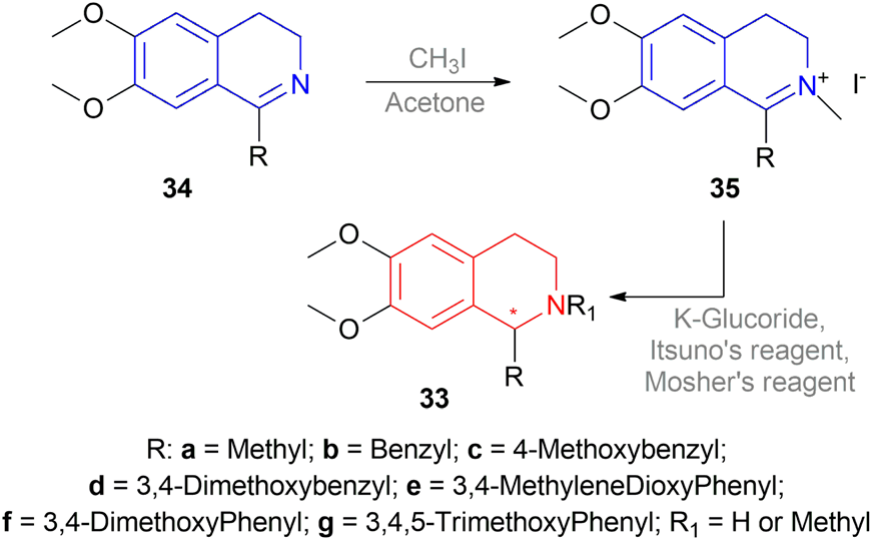
Reduction using K-glucoride, Itsuno's reagent, Mosher's reagent.

K-glucoride 30,^24^ Itsuno's reagent 31,^25,26^ and Mosher's reagent 32^27^ were previously utilized for asymmetric reduction of various ketones (Fig. 4). This led to Cho and Han to examine these reagents to stereoselectively synthesize 1-substituted-6,7-dimethoxy-THIQs 33 (Scheme 5).^28^

But they failed to enantioselectively reduce 1-substituted-6,7-dimethoxy-DHIQs 34 to 33 by asymmetric reduction with the above-mentioned chiral hydride reagents. 34a was reduced very poorly by 30 with 4 equiv. of excess hydride at room temperature. 31 increased yield with the addition of 1 equiv. AlCl_3_ but of low ee of 4.7%.

The authors then converted 34 to their quaternary salt 1-substituted-6,7-dimethoxy-dihydroisoquinolium iodide 35 with excess methyl iodide. This 35 was then readily reduced to 33 (Scheme 5).

35a in dry CH_2_CI_2_ was reduced by 1.1 equiv. 30 in THF at −78 °C with 52.3% ee. This reaction condition was also helpful in reducing 35e–35g with 25.2–43% ee; but not very good for 1-benzyl-6,7-dimethoxy-dihydroisoquinolium iodides 35b–35d. 31 (in THF at 30 °C) reduced 35a–35g with 5.9–21.1% ee while 32 (in THF at 0 °C) reduced 35a with 66.4% ee, and 35c–35g with 1.5–16% ee.

To conclude, 30 and 32 provide the best results for the reduction of 35a; 30 gave better results than those given by 31 and 32 for 1-aryl-6,7-dimethoxy-dihydroisoquinolium iodides (35e–35g); no hydrides gave sufficient results for 1-benzyl-6,7-dimethoxy-dihydroisoquinolium iodides (35b–35d).

So, 30 mainly gave (*R*)-configured products, 32 mainly gave (*S*)-configured products, and 31 gave (*S*) & (*R*) mix-configured products.

#### 2.1.4 Asymmetric reduction of 6,8-dimethoxy-1,3-dimethyl-DHIQ with LiAlH_4_/AlMe_3_

**Scheme 6:**
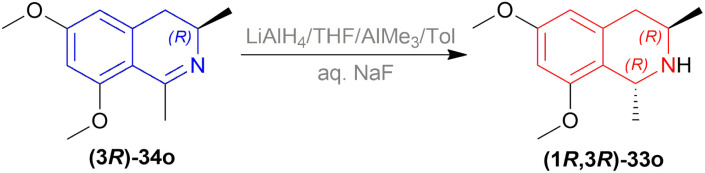
Reduction into (1*R*,3*R*)-6,8-dimethoxy-1,3-dimethyl-THIQ.

**Fig. 5:**
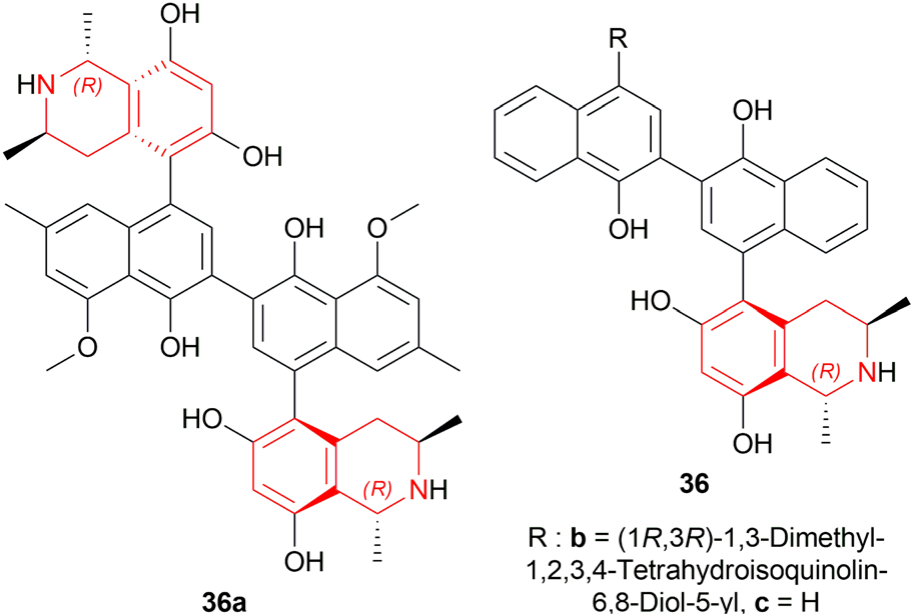
Michellamine A and its two analogs.

Michellamine A 36a is active against HIV strains in lymphocytes *in vitro*. Upender *et al.* synthesized two analogs of 36a which were 36b and 36c (Scheme 6) (Fig. 5).^29^

To produce the THIQ structure, (3*R*)-6,8-dimethoxy-1,3-dimethyl-DHIQ (3*R*)-34o was reduced with LiAlH_4_/AlMe_3_ to get (1*R*,3*R*)-6,8-dimethoxy-1,3-dimethyl-THIQ (1*R*,3*R*)-33o of which the chemical yield was 89% (Scheme 6). Here the single (1*R*,3*R*) diastereomer was obtained due to the presence of the (*R*)-methyl group in position 3.

#### 2.1.5 Synthesis of *O*,*O*-dimethylkorupensamine A

**Scheme 7:**
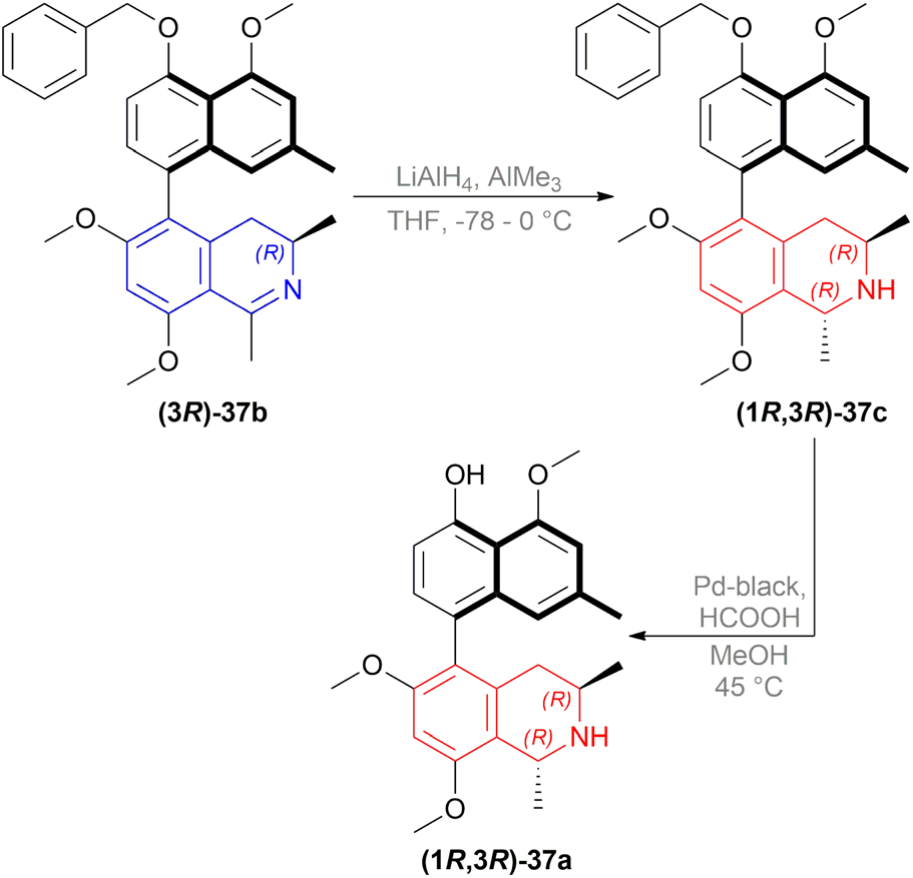
Synthesis of *O*,*O*-dimethylkorupensamine A.

Watanabe and Uemura synthesized *O*,*O*-dimethylkorupensamine A (1*R*,3*R*)-37a.^30^ In the last two steps of the synthesis, (3*R*)-5-(4-benzyloxy-5-methoxy-7-methylNaphthalen-1-yl)-6,8-dimethoxy-1,3-dimethyl-DHIQ (3*R*)-37b was reduced with LiAlH_4_, AlMe_3_ in THF to (1*R*,3*R*)-5-(4-benzyloxy-5-methoxy-7-methylNaphthalen-1-yl)-6,8-dimethoxy-1,3-dimethyl-THIQ (1*R*,3*R*)-37c in 70% chemical yield with 86% ee (Scheme 7). Then, debenzylation of (1*R*,3*R*)-37c was carried out with Pd-black and HCOOH in MeOH to produce *O*,*O*-dimethylkorupensamine A (1*R*,3*R*)-37a with 91% yield.

#### 2.1.6 Asymmetric reduction with sodium *N*,*N*-phthaloylamino acyloxy borohydride

**Scheme 8:**
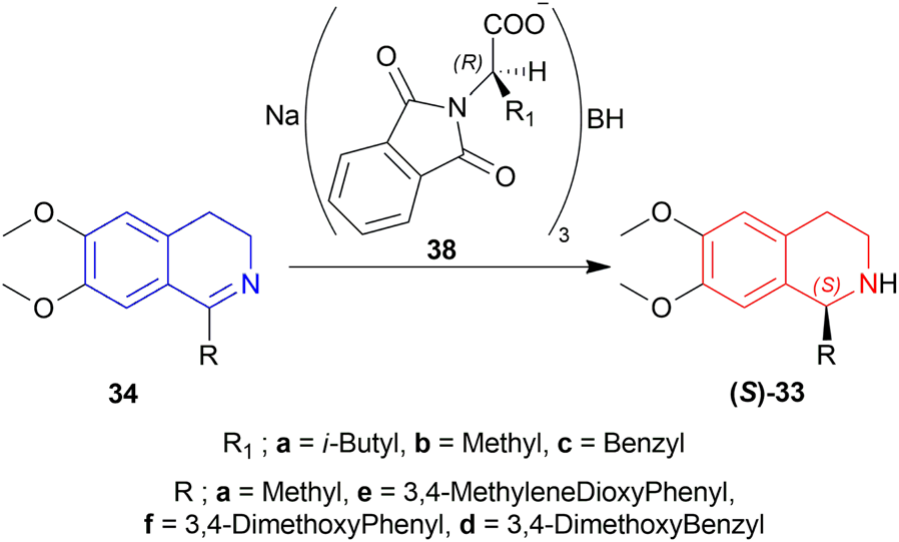
Asymmetric reduction of 1-substituted-6,7-dimethoxy-DHIQs with sodium *N*,*N*-phthaloylamino acyloxy borohydrides.

Hajipour and Hantehzadeh described the asymmetric reduction of 1-substituted-6,7-dimethoxy-DHIQs 34 with sodium *N*,*N*-phthaloylamino acyloxy borohydrides 38 (Scheme 8).^31^

33a, 33d–33f had ee of 65–75% (chemical yield 76–80%); 33a having the most (75% ee) and 33e having the least (65% ee) in the presence of 38a only. With ZnCl_2_ added with 38a, their ee increased to 72–80% (chemical yield 71–80%); 33a having the most (80% ee) and 33e having the least (72% ee) as before.

Among 38a–38c, 38a had yielded 78% of 33a with 71% ee in 5 h whereas 38b and 38c yielded 82% and 80% of 33a with 60% and 62% ee in 4 h. So, 38a was chosen for later reduction.

THF, diethyl ether, DCM, 1,2-dimethoxyethane, and 1,1,2,2-tetrachloroethane were experimented with as solvents. THF had yielded 78% of 33a with 71% ee in just 5 h reaction time without ZnCl_2_.

In solid state condition, 33a, 33d–33f had ee of 93–100% (chemical yield 80–90%); 33a had the most (100% ee) in 60 min reaction time and 33e had the least (93% ee) in 55 min reaction time.

So, 38a gave (*S*)-configured products.

### 2.2 Examples of enantioselective reduction of 1-substituted-DHIQs by hydrogenation with the help of a chiral catalyst

#### 2.2.1 Asymmetric hydrogenation (AH) with diphosphine-Ir(i) catalyst and phthalimide co-catalyst

**Fig. 6:**
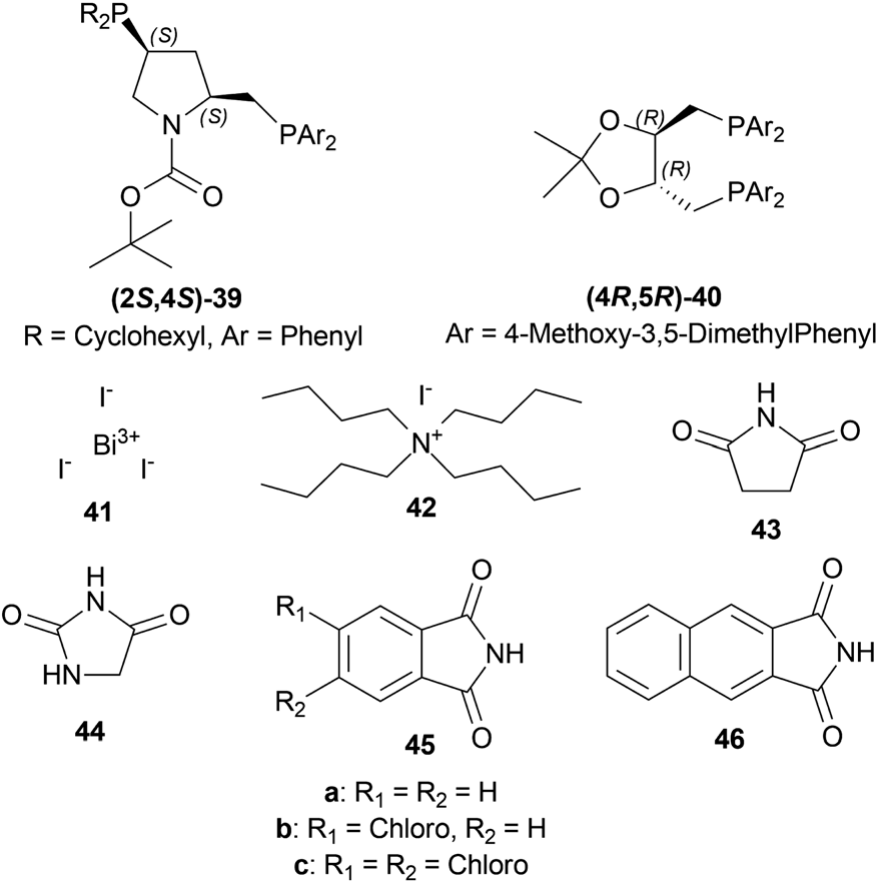
(2*S*,4*S*)-BCPM, (4*R*,5*R*)-MOD-DIOP and the co-catalysts.

Morimoto and Achiwa examined several co-catalysts for AH of 1-alkyl-6,7-dimethoxy-DHIQs 34 with diphosphine-Ir(i) as a catalyst.^32^ The ratio of substrate 34 : diphosphine : Ir(i) ligand [Ir(COD)CI]_2_ : co-catalyst was used as 200 : 2.4 : 1 : 4 with 100 atm H_2_. (2*S*,4*S*)-*tert*-butyl 4-(dicyclohexylphosphino)-2-((diphenyl-phosphino)methyl)pyrrolidine-1-carboxylate or (2*S*,4*S*)-BCPM (2*S*,4*S*)-39, [[(4*R*,5*R*)-5-[bis[(4-methoxy-3,5-dimethylphenyl)-phosphanyl]methyl]-2,2-dimethyl-1,3-dioxolan-4-yl]-(4-methoxy-3,5-dimethylphenyl)-phosphanylmethyl]-(4-methoxy-3,5-dimethylphenyl)phosphane or (4*R*,5*R*)-MOD-DIOP (4*R*,5*R*)-40 were the catalysts chosen for the test. Bismuth(iii) iodide 41, tetrabutylammonium iodide 42, succinimide 43, hydantoin 44, phthalimide 45a, 4-chlorophthalimide 45b, 4,5-dichloro-pthalimide 45c and 2,3-naphthalenedicarboximide 46 (Fig. 6) were the co-catalysts used.

**Scheme 9:**
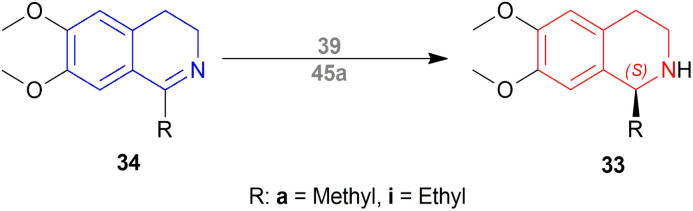
Reduction into (*S*)-salsolidine and (*S*)-1-ethyl-6,7-dimethoxy-THIQ.

With (2*S*,4*S*)-39, there was 10–18% ee of 33a from 34a (Scheme 9) when no co-catalyst or 41, 2 was used. But the ee increased to 43–93% with the use of 43, 44, 45a–45c, and 46. Less polar solvent and lower reaction temperature were the critical factors to increase enantioselectivity. For this reason, a maximum of 85–93% ee was observed for 45a co-catalyst in toluene as solvent at 2–5 °C reaction temperature.

With (4*R*,5*R*)-40, 45a in benzene–methanol solvent at −10 °C had 68% ee. So, (2*S*,4*S*)-39 was chosen as the intended one.

34a and 34i were reduced with (2*S*,4*S*)-39 and 45a to (*S*)-salsolidine (*S*)-33a and (*S*)-33i with 93% and 79% ee respectively (Scheme 9).

So, (2*S*,4*S*)-39 catalyst gave rise to (*S*)-configured products, and when the 1-substitution increases in length, % ee decreases.

#### 2.2.2 Asymmetric transfer hydrogenation (ATH) of 1-substituted-DHIQs

**Fig. 7:**
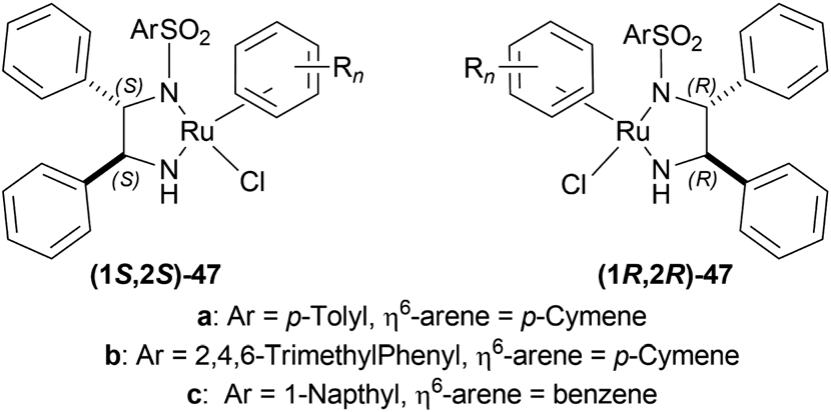
Ru(ii) catalysts for ATH of 1-substituted-DHIQs.

**Scheme 10:**
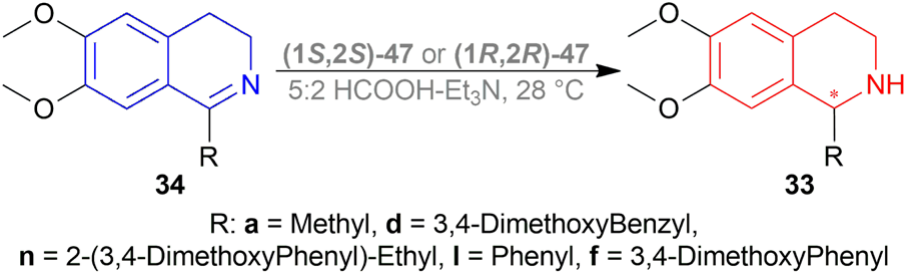
ATH of 1-substituted-DHIQs.

Uematsu *et al.* first described the ATH of 1-substituted-DHIQs 34 with the 5 : 2 HCOOH–Et_3_N azeotrope and catalysts 47 (Fig. 7) (Scheme 10).^33^

For screening, 1-methyl-6,7-dimethoxy-DHIQ 34a (5 mmol) was reduced asymmetrically to (*R*)-salsolidine (*R*)-33a (>99% yield, 95% ee) with (1S,2S)-47a, 5 : 2 HCOOH–Et_3_N azeotrope mixture in ACN at 28 °C, *S*/*C* = 200 : 1, HCOOH/34a = 6 : 1 for 3 h. The same result was found when the catalyst was prepared *in situ* without isolating. Without Et_3_N, no (*R*)-33a was produced. The authors also showed that the asymmetric reduction was caused by the transfer hydrogen by formic acid, no molecular H_2_ interferes here. The above reaction is done under a 600 atm D_2_ atmosphere (where D_2_ : HCOOH = 24 : 1) gave (*R*)-33a (>99% yield, 93% ee) without D_2_ incorporated into the C1 position. Again, 2-propanol-2-d with HCOOH or CH_3_COOH showed that 2-propanol (IPA) can not be used as a hydrogen source.

Then increasing *S*/*C* of 34a/(1*S*,2*S*)-47a to 1000 : 1 in a 20 mmol scale reaction produced 97% yield and 94% ee of (*R*)-33a in 12 h in ACN. 34d and 34n with (1*R*,2*R*)-47b (*S*/*C* = 200 : 1) in *N*,*N*-dimethylformamide (DMF), and DCM afforded 90% and 99% yield; 95% and 92% ee of (*S*)-33b and (*S*)-33c in 7 and 12 h respectively. 34l with (1*S*,2*S*)-47c (*S*/*C* = 200 : 1) in DCM had 99% yield and 84% ee of (R)-33d in 8 h 34f with (1*R*,2*R*)-47c (*S*/*C* = 100 : 1) in DCM gained >99% yield and 84% ee of (*S*)-33e in 12 h (Scheme 10).

So, (*S*)-configurated 47 catalysts afforded (*R*)-configurated products and (*R*)-configurated 47 catalysts afforded (*S*)-configurated products in good % ee.

**Scheme 11:**
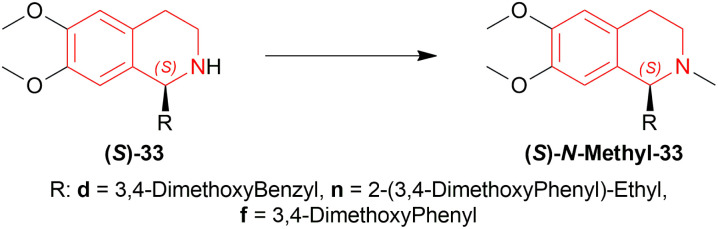
Synthesis of (*S*)-laudanosine, (*S*)-homolaudanosine, and (*S*)-cryptostyline II.

*N*-methylation of (*S*)-33d, (*S*)-33n, and (*S*)-33f can result in naturally occurring (*S*)-*N*-methyl-33d (*S*)-laudanosine, (*S*)-*N*-methyl-33n (*S*)-homolaudanosine, and (*S*)-*N*-methyl-33f (*S*)-cryptostyline II, respectively (Scheme 11).

#### 2.2.3 Production of (*S*)-norlaudanosine, (*S*)-tetrahydrohomopapaverine, and (*R*)-norcryptostyline II by catalytic asymmetric hydrogenation

**Fig. 8:**
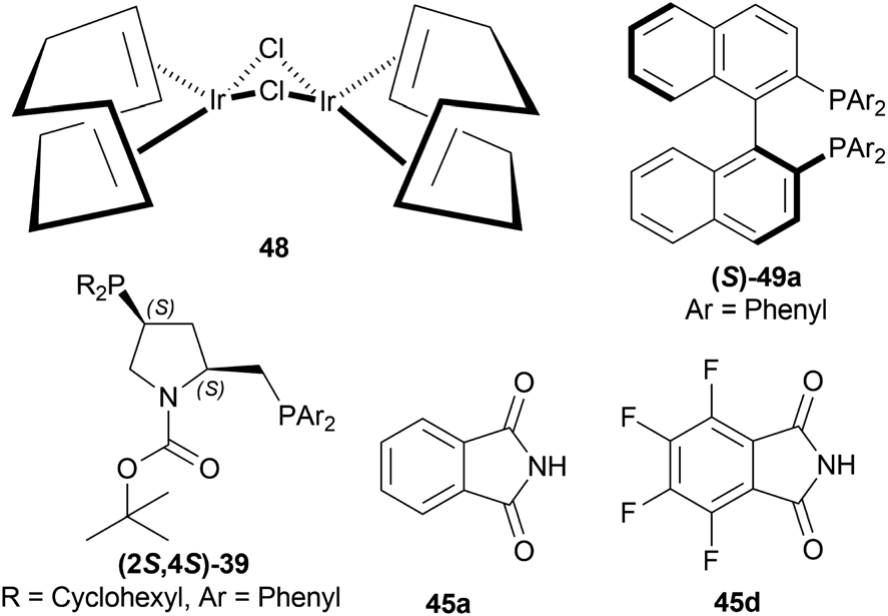
[Ir(COD)Cl]_2_, (*S*)-BINAP, (2*S*,4*S*)-BCPM, phthalimide, and 3,4,5,6-tetrafluorophthalimide.

Morimoto *et al.* hydrogenated 34d, 34n, and 34f catalytically with different chiral diphosphine-Ir(i)-phthalimide complex catalysts to produce (*S*)-norlaudanosine (*S*)-33d, (*S*)-tetra-hydroHomoPapaverine (*S*)-33n and (*R*)-norcryptostyline II (*R*)-33f.^34^ Chloro(1,5-cyclooctadiene)iridium(i) dimer or [Ir(COD)Cl]_2_48, (*S*)-2,2′-bis(diphenylphosphino)-1,1′-binaphthyl or (*S*)-BINAP (*S*)-49a, (2*S*,4*S*)-BCPM (2*S*,4*S*)-39, phthalimide 45a, and 3,4,5,6-tetrafluorophthalimide 45d (Fig. 8) were used in this experiment.

When reducing 34d to (*S*)-33d, 48, (*S*)-49a with methanol (MeOH) had a low conversion of 18% and 35% ee. But when 45a was added to 48, (*S*)-49a, and MeOH, conversion rose to 67% with 84% ee. Also 48, 49 with 45d had the highest conversion of 84% and 88% ee.

**Scheme 12:**
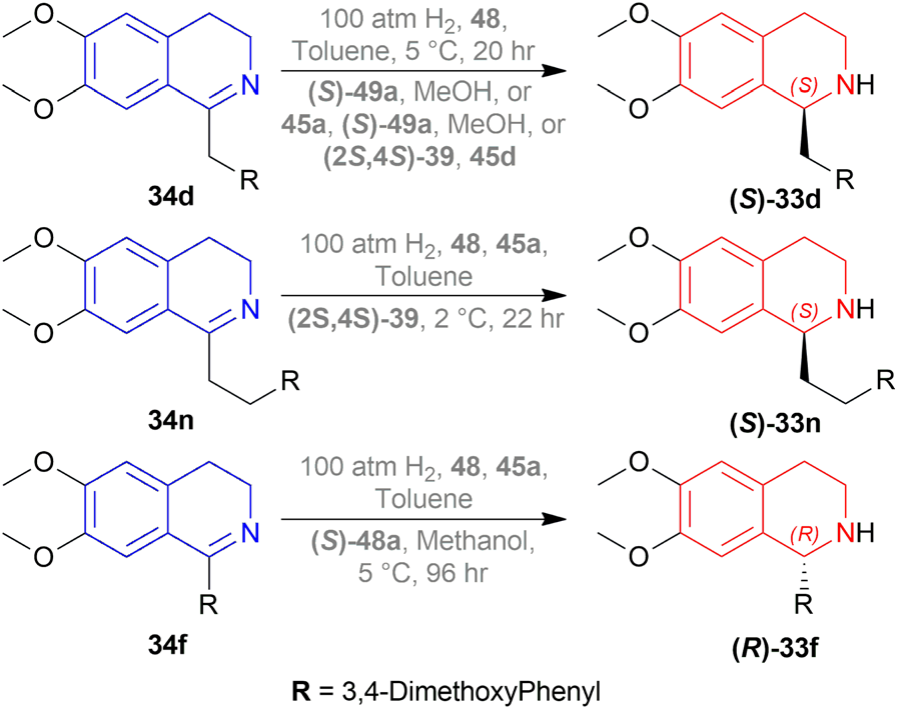
Reduction into (*S*)-norlaudanosine, (*S*)-tetrahydrohomopapaverine, and (*R*)-norcryptostyline II.

With 48, 49, and 45a, reducing 34n gave (*S*)-33n up to 75% conversion and 87% ee, though 48, 45a, and (*S*)-49a with MeOH reduced 34f to (*R*)-33f with 50% conversion and 31% ee (Scheme 12).

So, (2*S*,4*S*)-39 and (*S*)-49 catalysts gave rise to (*S*)-configured products, but when the 1-substitution increases steric hindrance, (*R*)-configured product is found.

#### 2.2.4 Asymmetric reduction of 6,8-dimethoxy-1,3-dimethyl-DHIQ with Pd/C

**Fig. 9:**
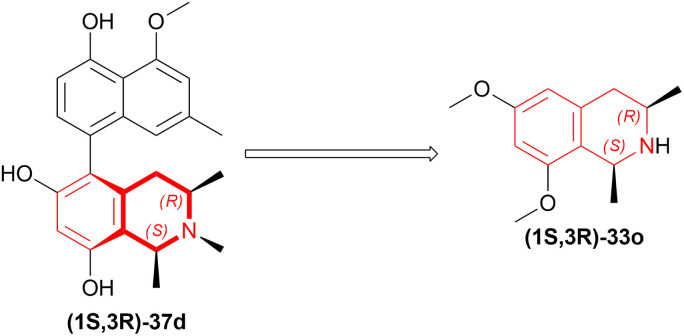
Korupensamine D and it's precursor.

Hoye and Chen carried out a total synthesis of Korupensamine D (1*S*,3*R*)-37d (Fig. 9) for the very first time.^35^

**Scheme 13:**
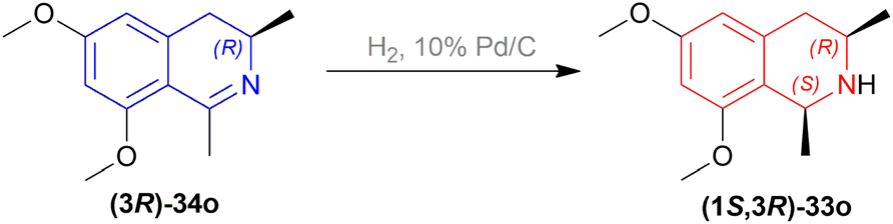
Reduction of (3*R*)-6,8-dimethoxy-1,3-dimethyl-DHIQ.

In the fifth step of this synthesis, they reduced (3*R*)-6,8-dimethoxy-1,3-dimethyl-DHIQ (3*R*)-33o to optically pure (1*S*,3*R*)-6,8-dimethoxy-1,3-dimethyl-THIQ (1*S*,3*R*)-32o with H_2_, 10% Pd/C and had 93% conversion (Scheme 13).

#### 2.2.5 Synthesis of (*S*)-calycotomine *via* AH

**Scheme 14:**
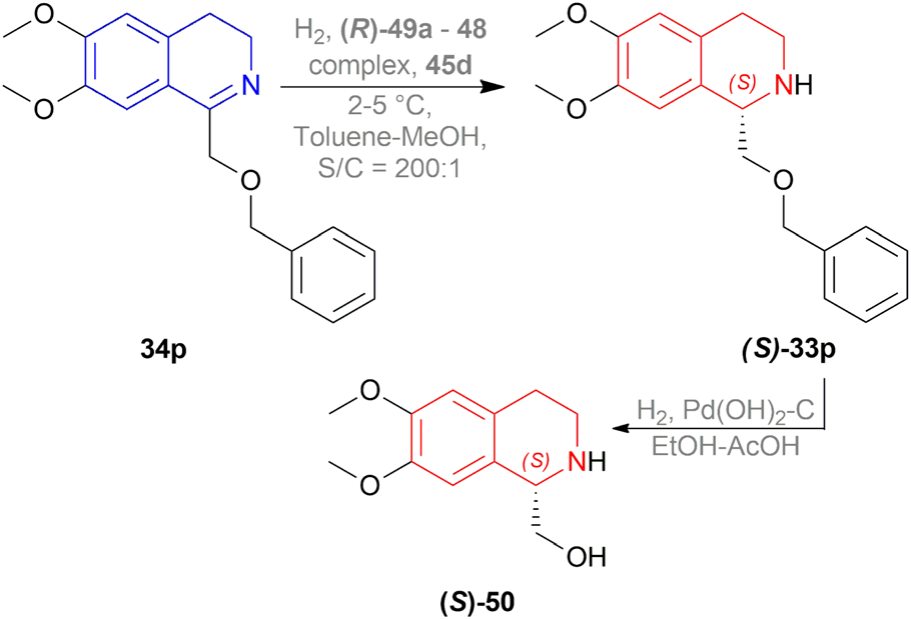
Synthesis of (*S*)-calycotomine.

(*S*)-calycotomine (*S*)-50 is a naturally occurring alkaloid. To synthesize (*S*)-50, Morimoto *et al.* reduced 1-benzyloxymethyl-6,7-dimethoxy-DHIQ 34p by AH to (*S*)-1-benzyloxymethyl-6,7-dimethoxy-THIQ (*S*)-33p (85% yield and 86% ee) with 100 atm H_2_, 0.5 mol% (*R*)-49a–48 complex, 1 mol% 45d (Fig. 8) in toluene–methanol (Scheme 14).^36^ If 45a (Fig. 8) was used as an additive, lower enantioselectivity (75% ee) was observed. Then, hydrogenolysis of the benzyloxy group of (*S*)-33p was carried out by 1 atm H_2_, Pd(OH)_2_ catalyst in a 10 : 1 mixture of ethanol–acetic acid to yield 93% of (*S*)-50 with 86% ee.

**Scheme 15:**
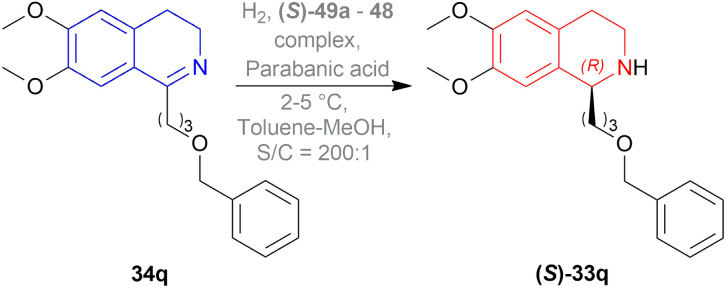
Reduction into 1-[3-(benzyloxy)propyl]-6,7-dimethoxy-THIQ.

They also hydrogenated 1-[3-(benzyloxy)propyl]-6,7-dimethoxy-DHIQ 33q under the above-mentioned asymmetric hydrogenation process to yield 99% of 1-[3-(benzyloxy)propyl]-6,7-dimethoxy-THIQ (*S*)-32q using (*S*)-49a–48 complex (Fig. 8) and parabanic acid (Scheme 15). The product (*S*)-33q was obtained in 89% ee.

So, (*R*)-49a catalyst gave rise to (*S*)-configured product when the benzyloxy part was closer to the 1-substitution, and (*S*)-49a catalyst gave rise to (*S*)-configured product when the benzyloxy part was far away from the 1-substitution.

#### 2.2.6 Asymmetric transfer hydrogenation (ATH) by chiral rhodium complexes

**Fig. 10:**
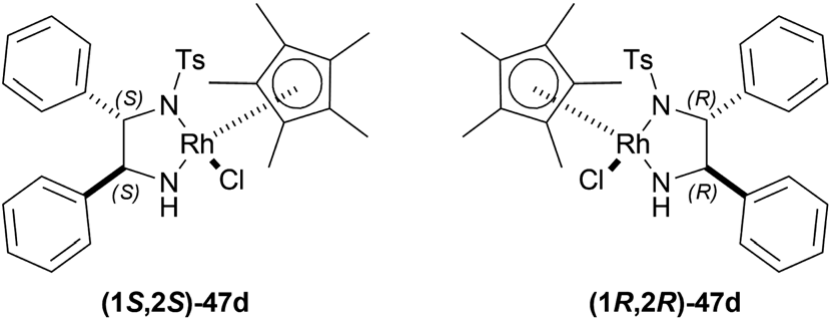
(1*S*,2*S*)-Cp*RhClTsDPEN and (1*R*,2*R*)-Cp*RhClTsDPEN.

Mao and Baker catalysed 1-substituted-6,7-dimethoxy-DHIQs 34 to 1-substituted-6,7-dimethoxy-THIQs 33 by ATH with the help of (1*S*,2*S*)-Cp*RhClTsDPEN (1*S*,2*S*)-47d, a chiral rhodium complex and its enantiomer (1*R*,2*R*)-Cp*RhClTsDPEN (1*R*,2*R*)-47d (Fig. 10).^37^ As the hydrogen source, 5 : 2 HCOOH–Et_3_N azeotrope was used. ATH is advantageous over other reduction processes because it does not require the use of H_2_ (g).

**Scheme 16:**
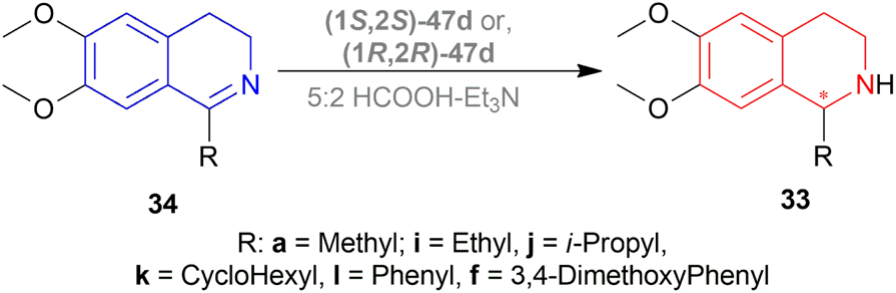
Reduction with (1*S*,2*S*)-Cp*RhClTsDPEN and (1*R*,2*R*)-Cp*RhClTsDPEN.

At first, 34j was catalyzed with (1*S*,2*S*)-47d along with substrate/catalyst (*S*/*C*) molar ratio of 200 : 1, 5 : 2 HCOOH–NEt_3_ azeotrope, 20 °C temperature, DCM as solvent (Scheme 16). After 10 minutes, (*R*)-33j was found with 99% ee. But with an *S*/*C* molar ratio of 1000 : 1, ee reached 93% after 60 minutes. The use of acetonitrile (ACN) as solvent produced a slightly higher ee of 95% at *S*/*C* of 1000 : 1 after 60 minutes.

34a was hydrogenated using (1*S*,2*S*)-47d, ACN as solvent to produce (*R*)-33a in 89% ee; (*S*)-33a was produced in 90% ee with (1*R*,2*R*)-47d, DCM as solvent in a *S*/*C* molar ratio of 200 : 1 after 10 minutes of reaction. 34i was catalyzed using (1*S*,2*S*)-47d with dropped 83% ee of the product (*R*)-33i, while 34k was catalyzed using the same catalyst with an increased 97% ee of the product (*R*)-33k. These showed that the ee stayed within a good range because the substituted alkyl group, R, increased in steric bulk on the imine carbon of 34.

But when aryl groups were introduced, such as in 34l and 34f, they blocked the catalyst (1*S*,2*S*)-47d to bind and as a result, ee dropped dramatically {4.4% and 3.2% respectively for (*R*)-33l and (*R*)-33f, even after 180 minutes}. Here, the second aromatic ring system intervenes with the selective catalyst binding.

So, (1*S*,2*S*)-47d catalyst gave rise to (*R*)-configured products, and (1*R*,2*R*)-47d catalyst gave rise to (*S*)-configured products.

#### 2.2.7 AH by ruthenium-optically active phosphine complex

**Scheme 17:**
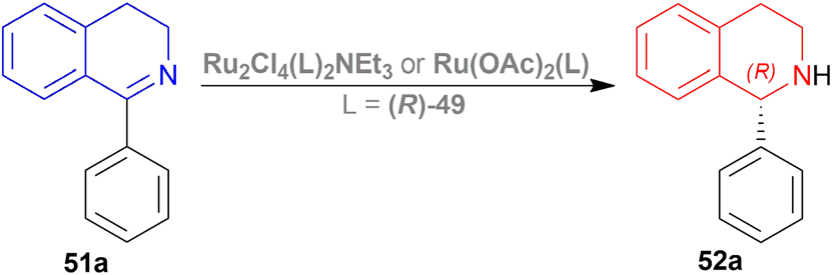
Synthesis of 1-phenyl-THIQ.

Kuriyama *et al.* described the process of converting 1-phenyl-DHIQ 51a to 1-phenyl-THIQ 52a by AH (Scheme 17).^38^ It is done in presence of a ruthenium-optically active phosphine complex derived from an optically active phosphine.

**Fig. 11:**
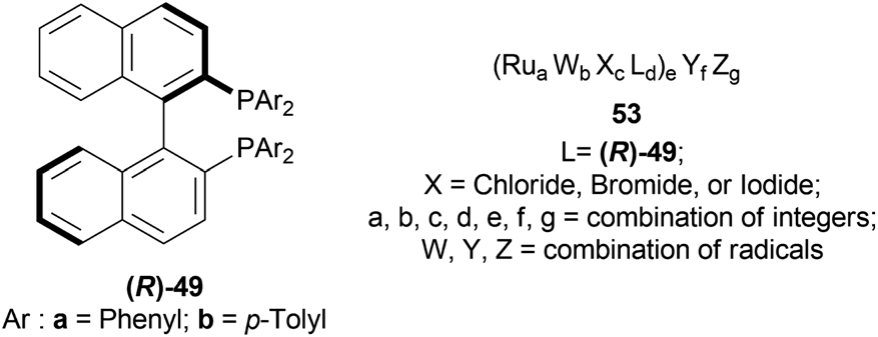
(*R*)-BINAP, (*R*)-T-BINAP, and the ruthenium-optically active phosphine complex.

The optically active phosphines are (*R*)-BINAP (*R*)-49a, and (*R*)-T-BINAP (*R*)-49b (Fig. 11). The ruthenium-optically active phosphine complex has the general formula of 53*e.g*., Ru_2_Cl_4_(L)_2_NEt_3_, Ru(OAc)_2_(L), *etc*. Here, L is the optically active phosphines (*R*)-49 (Fig. 11).

There are 4 examples of the invention described in this patent which had a conversion rate of 56.9–91.4% and ee of 59.7–88.9%. Example 1 had the highest ee of 88.9% with the lowest conversion of 56.9% for the product (*R*)-52a. Ru_2_Cl_4_{(*R*)-T-BINAP}_2_NEt_3_ and 51a were used in approximately 1 : 400 molar ratio. Toluene was added after N_2_ purge, stirred for 15 h at 90 °C, and 6 MPa H_2_ pressure after H_2_ purge. Example 2 showed 59.7% ee and 89.3% conversion of (*R*)-52a. Ru_2_Cl_4_{(*R*)-T-BINAP}_2_NEt_3_ and HCl salt of 51a was used in approximately 1 : 400 molar ratio. MeOH was added after N_2_ purge, stirred for 19 h at 90 °C, and 3 MPa H_2_ pressure after H_2_ purge, toluene and 1 M NaOH (aq) solution were added, and stirred.

Example 3 had 66.7% ee with 91.4% conversion of (*R*)-52a. Ru_2_Cl_4_{(*R*)-BINAP}_2_NEt_3_ and 51awere used in approximately 1 : 1000 molar ratio. Toluene and formic acid were added after N_2_ purge, stirred for 15 h at 90 °C, and 3 MPa H_2_ pressure after H_2_ purge, toluene and 1 M NaOH (aq) solution were added and stirred. And, example 4 showed 65.4% ee and 83.1% conversion of (*R*)-52a. Ru(OAc)_2_{(*R*)-BINAP} and HCl salt of 51awere used in approximately 1 : 200 molar ratio. MeOH and methyl salicylate were added after N_2_ purge, stirred for 19 h at 90 °C and 3 MPa H_2_ pressure after H_2_ purge, toluene and 1 M NaOH (aq) solution was added and stirred.

So (*R*)-49a catalyst, used as phosphine in a ruthenium-optically active phosphine complex, gave rise to (*R*)-configured products. The addition of toluene and 1 M NaOH (aq) solution to the reaction mixture and stirring at the last step, increased the conversion rate of the product but decreased the % ee.

#### 2.2.8 AH with ionic Cp*Rh(iii) catalyst

**Scheme 18:**
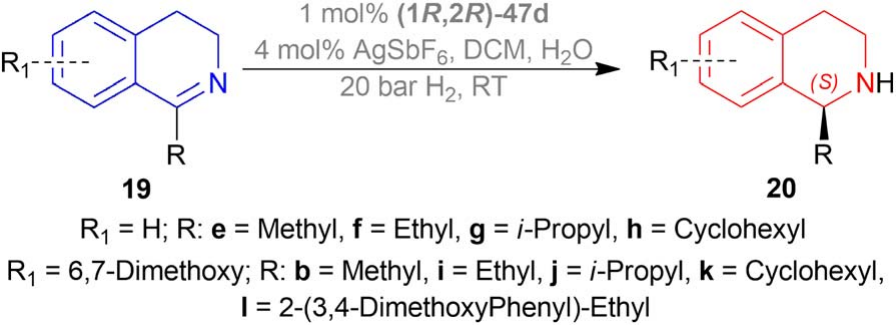
AH with ionic Cp*Rh(iii) catalyst.

Li *et al.* hydrogenated 1-alkyl-DHIQs 19b, 19e–19l with 50 bar H_2_ in the presence of 1 mol% (1*R*,2*R*)-47d (Fig. 10), 4 mol% AgSbF_6_ in DCM with water (Scheme 18).^39^

(*S*)-20e-(*S*)-20h had 90–95% yield but, as the bulkier group were introduced in the 1-position, % ee decreased and reaction time increased. When IPA was used instead of DCM, % ee of (*S*)-20g and (*S*)-20h increased to 83–91%.

(*S*)-20b, (*S*)-20i-(*S*)-20l had 90–95% yield and 93–99% ee. So, not only the steric hindrance but also the electron availability are the critical factors.

So, (1*R*,2*R*)-47d produced (*S*)-configured products.

#### 2.2.9 AH in aqueous media with Noyori type catalysts

**Fig. 12:**
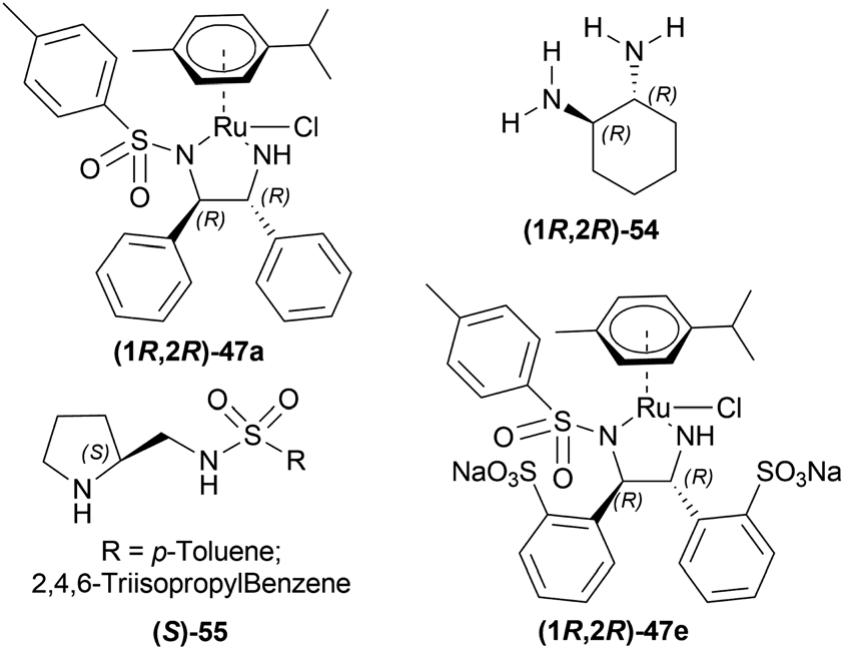
Catalysts for AH in aqueous media.

Canivet *et al.*, Canivet and Süss-Fink previously reported the use of a catalyst based on (1*R*,2*R*)-1,2-diaminocyclohexane (1*R*,2*R*)-54^40^ and (*S*)-(sulfonylamino)methylpyrrolidines (*S*)-55^41^ chiral ligands for reactions in aqueous solution. Ma *et al.*, and Wu *et al.* have also used (1*R*,2*R*)-47e, a sulfonated variant of (1*R*,2*R*)-47a (Fig. 12).^42,43^

**Scheme 19:**
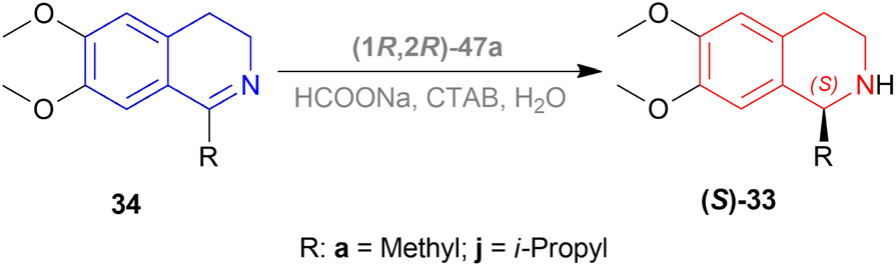
Reduction with sodium formate (aq) solution and CTAB.

Evanno *et al.* reported asymmetric hydrogen-transfer reductions of 1-substituted-6,7-dimethoxy-DHIQs 34 (Scheme 19) with sodium formate (aq) as hydride source and or cetyltrimethylammonium bromide (CH_3_–(CH_2_)_15_–N^+^(CH_3_)_3_Br^−^, CTAB) as cationic surfactant.^44^ Using (1*R*,2*R*)-47a, they obtained the products (*S*)-33a and (*S*)-33j in excellent ee (99% at 25 °C and 99.5% at 40 °C respectively) (Scheme 19).

So, (1*R*,2*R*)-47a catalyst gave rise to (*S*)-configured products.

#### 2.2.10 ATH in water

**Fig. 13:**
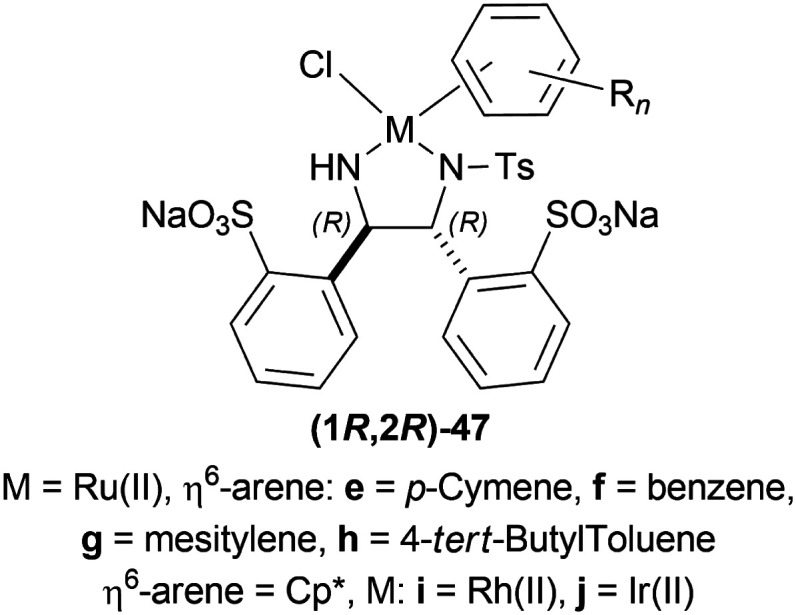
Metal complexes of *O*,*O′*-disulfonated *N*-tosyl-1,2-diphenylethylene diamine.

Wu *et al.* described ATH of 1-substituted-6,7-dimethoxy-DHIQs 34 in water with the help of metal complexes of *O*,*O*′-disulfonated *N*-tosyl-1,2-diphenylethylene diamine (1*R*,2*R*)-47 (Fig. 13).^43^

**Scheme 20:**
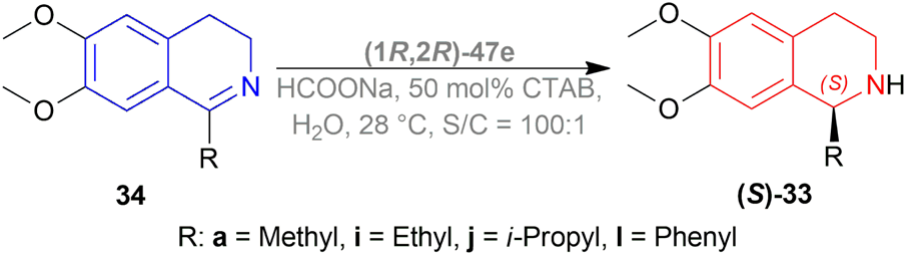
Reduction of 1-substituted-6,7-dimethoxy-DHIQs.

When no surfactant was used with (1*R*,2*R*)-47e and *S*/*C* = 100 : 1 for 10 h of reaction time at 28 °C, 89% yield was found for (*S*)-33a from 34a (90% ee). Adding 50 mol% of sodium dodecyl sulfate (SDS), poly(ethylene glycol) mono [4-(1,1,3,3-tetra-methylbutylphenyl) ether] (triton X-100), cetylpyridium bromide (CPB), 3-(*N*,*N*-dimethyldodecylammonio)propane-sulfonate (DDAPS), or tetrabutylammonium bromide (TBAB) did not improve % ee. But with CTAB, the yield was 97% with 95% ee. Increasing or decreasing the mol% of CTAB, and increasing the reaction temperature also did not improve the % ee. Other complexes like (1*R*,2*R*)-47f-(1*R*,2*R*)-47j had similar results but the reaction time was 20–48 h. So, (1*R*,2*R*)-47e with 50 mol% CTAB was chosen. Then, 34i and 34j were reduced with the optimum conditions for 25 h and their yield was 68% (92% ee) and 90% (90% ee) respectively (Scheme 20). But 34l did not react even after 72 h.

**Scheme 21:**
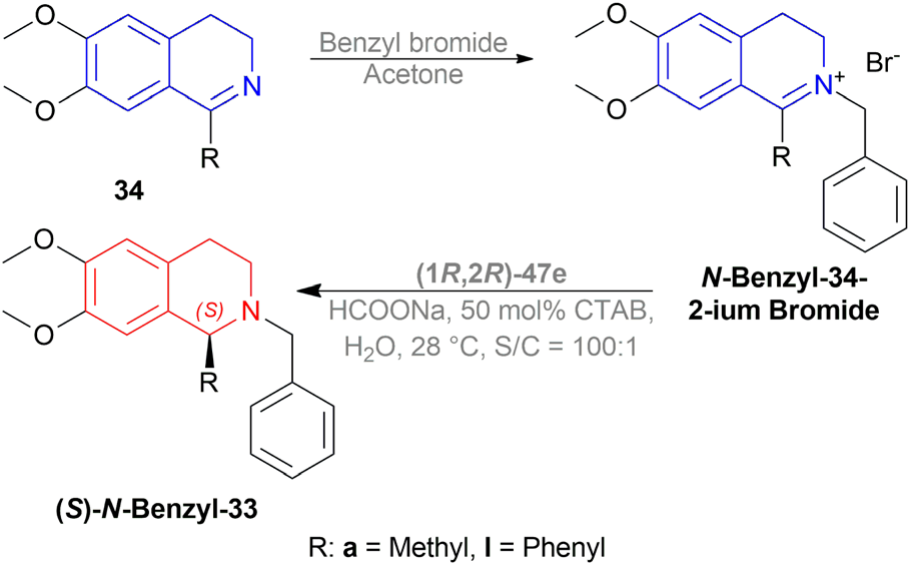
Synthesis of 1-substituted-2-benzyl-6,7-dimethoxy-THIQs.

The authors then benzylated 34a and 34l with benzyl bromide in acetone to produce 1-substituted-2-benzyl-6,7-dimethoxy-3,4-dihydroisoquinolin-2-ium bromides. These were reduced readily with the optimum conditions to yield 86% (*S*)-*N*-benzyl-33a (90% ee) and 94% (*S*)-*N*-benzyl-33l (95% ee) (Scheme 21).

So, (1*R*,2*R*)-47e catalyst gave rise to (*S*)-configured products.

#### 2.2.11 ATH with ruthenium catalyst

**Scheme 22:**
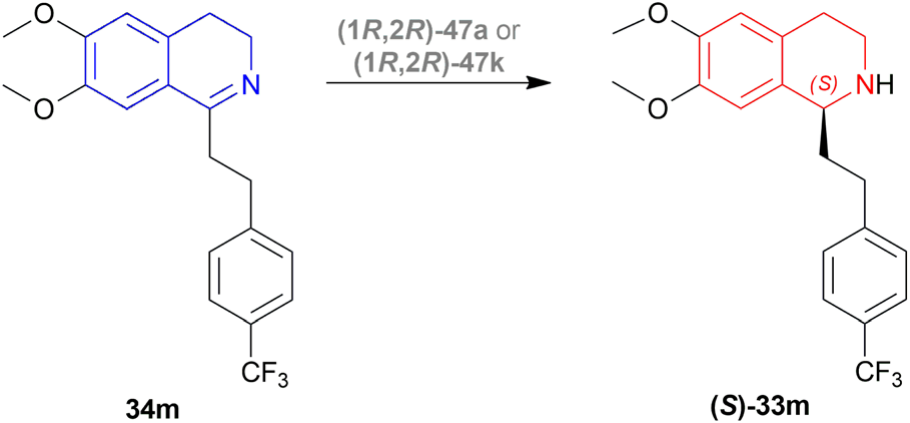
Production of (*S*)-6,7-dimethoxy-1-[2-(4-trifluoromethylphenyl)ethyl]-THIQ.

**Fig. 14:**
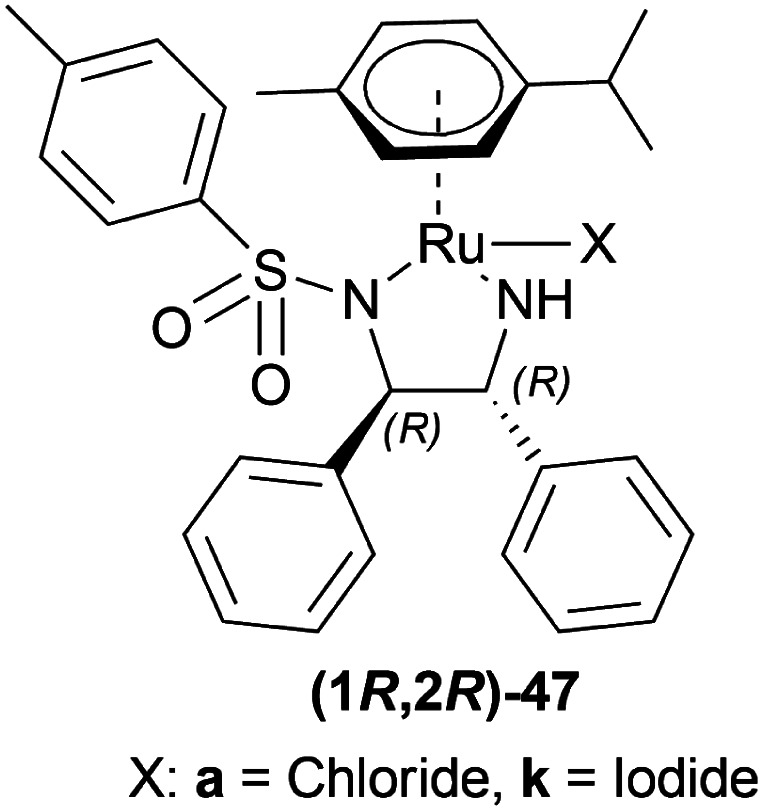
Ruthenium catalysts for ATH of 6,7-dimethoxy-1-[2-(4-trifluoromethyl-phenyl)ethyl]-DHIQ.

Rádl *et al.* described the process of ATH of 6,7-dimethoxy-1-[2-(4-trifluoromethylphenyl)ethyl]-DHIQ 34m to produce (*S*)-6,7-dimethoxy-1-[2-(4-trifluoromethylphenyl)ethyl]-THIQ (*S*)-33m (Scheme 22) along with (1*R*,2*R*)-47 as catalysts (Fig. 14).^45^

There were 10 examples of this process of hydrogenation in which 34m was reduced to (*S*)-33m with 59–100% ee and 52–88% conversion. Only example no. 2, 4, 6, and 9 had more than 80% conversion and 99.4–100% ee (Scheme 22).

Example 2 had a 4 : 3 mix of HCOOH and TEA with (1*R*,2*R*)-47a directly added, the mixture was stirred in N_2_ at 30 °C for 3 hours. With 7.5 mmol of 33m in 30 mL dimethylfumarate, the mixture was evaporated and evaporation residue dissolved in 30 mL of DCM, washed with NaHCO_3_, water, and brine saturated solution, then dried with MgSO_4,_ and solvent was evaporated, the residue dissolved in IPA and ethanolic solution of HCl (5 M, 3 mL) dropwise under intensive stirring, the solvent was evaporated, then the residue was crystallized out of an IPA/MeOH mixture. 83% conversion with 99.6% ee was found for this example.

In the case of example no. 4 and 9, (1*R*,2*R*)-47a was prepared *in situ*, and TEA solution in ACN by stirring at 80 °C for 1 h 4 : 3 mix of HCOOH and TEA was added with 33m in dimethylfumarate, and dimethylfumarate with TEA respectively; stirred at 35 °C in N_2_ for 3 hours. In example 4, the reaction mixture was evaporated, the residue dissolved in 250 mL ethyl acetate, and the rest of the process was the same as in example no. 2 with 88% conversion and 99.8% ee. While, example 9 reaction mixture was poured into water, extracted 3 times with ethyl acetate, washed 3 times with brine, dried with MgSO_4_, dropwise addition of HCl (g) in ethanol, stirred for 30 min, then the solid is sucked off, washed with ethyl acetate, dried, recrystallized out of an IPA/MeOH mixture. 86% conversion with 100% ee was found for this example. We think that example 9 is the best process for ATH of 34m to (*S*)-33m.

In example 6, (1R,2R)-47k (Fig. 14) was prepared *in situ*, THF was solvent of 34m, the mixture was stirred at 40 °C in N_2_ for 6 h, and the rest of the process was the same as example 2 with 82% conversion and 99.4% ee.

So, (1*R*,2*R*)-47a and (1*R*,2*R*)-47k catalysts gave rise to (*S*)-configured products.

#### 2.2.12 AH with synphos ligand and iridium catalyst

**Fig. 15:**
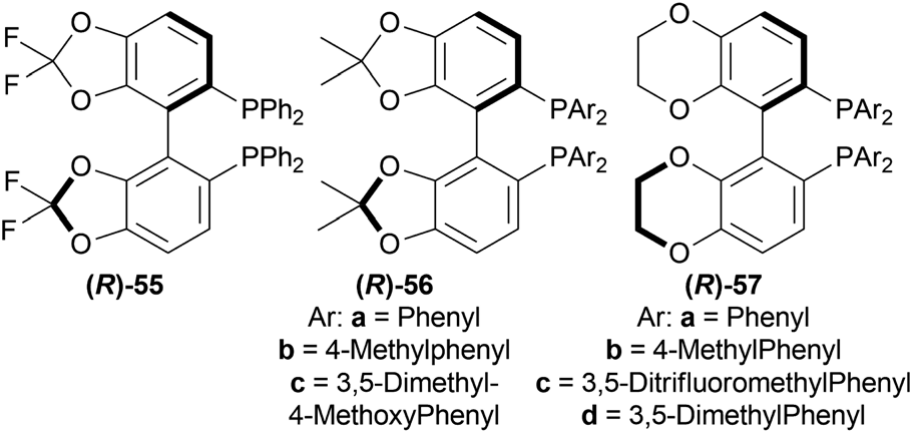
Difluorphos ligand, sunphos ligands, and synphos ligands.

**Scheme 23:**
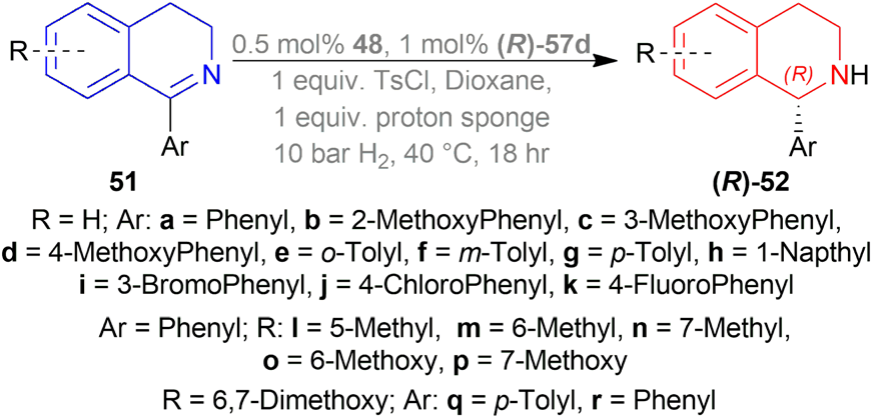
Generation of (*R*)-1-aryl-THIQs.

Berhal *et al.* experimented with 1 mol% difluorphos ligand (*R*)-55, sunphos ligands (*R*)-56a-(*R*)-56c, and synphos ligands (*R*)-57a-(*R*)-57d (Fig. 15) in THF along with 0.5 mol% 48 (Fig. 8), 30 bar H_2_ for 18 h at 40 °C for AH of 1-phenyl-DHIQ 51a to (*R*)-1-phenyl-THIQ (*R*)-52a (Scheme 23).^46^ Among the ligands, 55 and (*R*)-56a-(*R*)-56c gave moderate ee of 35–39%; (*R*)-57a-(*R*)-57d improved ee to 46, 42, 69, and 71% respectively. So, (*R*)-3,5-diMe-synphos (*R*)-57d was selected as the ligand.

Dioxane was chosen as solvent as it maximized chemical yield to 95% and optical yield ee to 73%. 1 equivalent tosyl chloride was successful in increasing ee to 92% as an additive but a side product was formed which was nullified by using 1 equivalent proton sponge as a base. Using 10 bar H_2_ gave the highest yield of 95% and ee of 94%.

Lastly, 1-aryl-DHIQs 51a–51r were hydrogenated with the above conditions to produce (*R*)-1-aryl-THIQs (*R*)-52a-(*R*)-52r in good yields and the ee varied from 81–94% (Scheme 23). This variation was due to substitution in the aromatic rings. (*R*)-52b, (*R*)-52e, and (*R*)-52h (*ortho*-substituted in the 1-phenyl ring) had 83, 80 and 82% ee respectively compared to (*R*)-52a (94% ee) because of steric hindrance.

(*R*)-52i-(*R*)-52k (bearing electron-withdrawing group in the 1-phenyl ring) had 82, 81, and 84% ee respectively; while (*R*)-52d, (*R*)-52f, (*R*)-52g (bearing electron-donating group in the 1-phenyl ring) had 90% ee each. Methyl substitution in the 5, 6, or 7 position of the THIQs had lower ee (84, 84, and 88% for (*R*)-52l – (*R*)-52n respectively) than one or two methoxy substitution in the 6, or/and 7 position of the THIQs (88, 89, 88, and 92% ee for (*R*)-52o-(*R*)-52r respectively). Even after single crystallization, the ee can be enhanced to 90–99%.

So, (*R*)-55, (*R*)-56, and (*R*)-57 catalysts gave rise to (*R*)-configured products.

#### 2.2.13 AH with taniaphos ligand and iridium catalyst

**Scheme 24:**
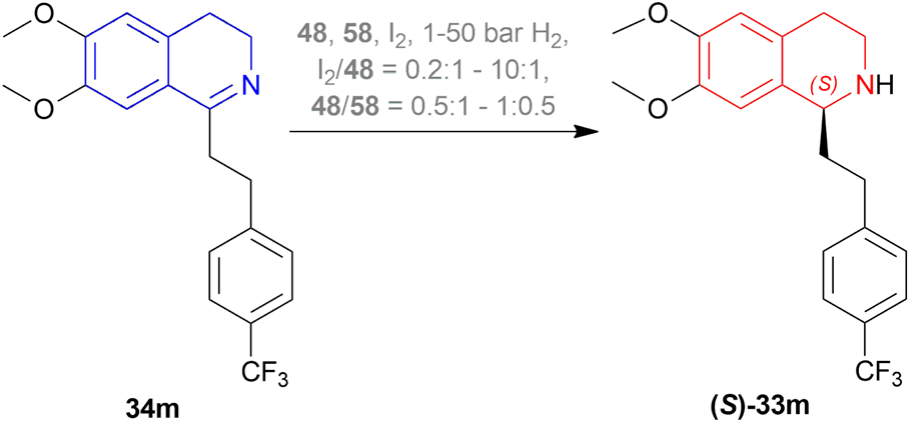
AH of 6,7-dimethoxy-1-[2-(4-trifluoromethyl-phenyl)ethyl]-DHIQ.

**Fig. 16:**
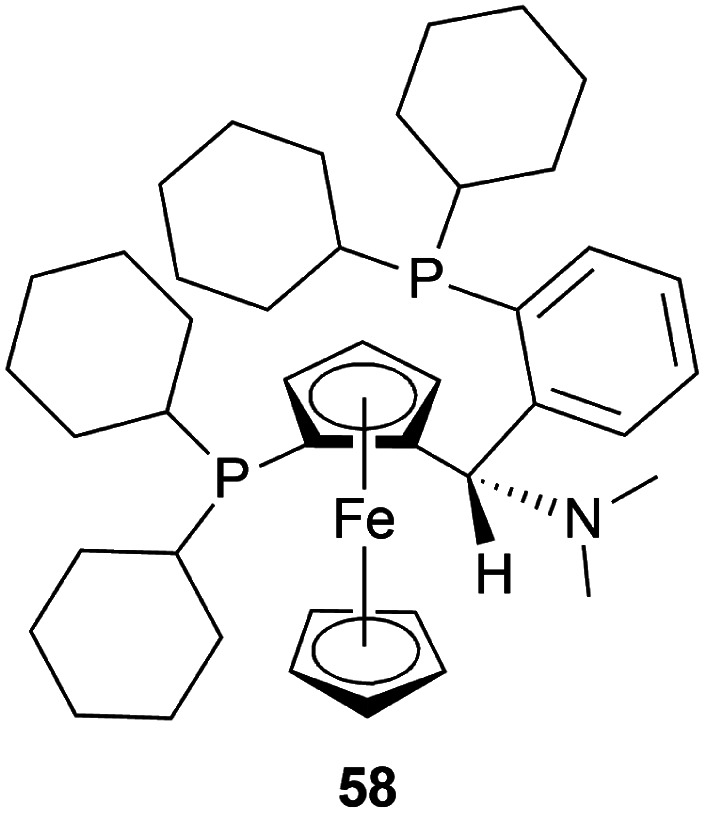
Taniaphos ligand.

Bappert *et al.* stated 3 methods of hydrogenation of (*S*)-33m from 34m (Scheme 24) with taniaphos ligand 58 (Fig. 16),^47^48 (Fig. 8), I_2_, 1–50 bar H_2_.^48^ I_2_/48 is needed in between 0.2 : 1 and 10 : 1, and the ratio of 48/58 is in between 0.5 : 1 and 1 : 0.5 (Scheme 24).

The first method involved 58 adding to a solution of 48 in DCM at 20 °C, and a solution of I_2_ in DCM is added to it until dissolved. Then 34m is added to this at 5 bar H_2_. I_2_/48 ratio of 1 : 1 and *S*/*C* ratio of 20 : 1 had a full conversion and 89% ee. When the *S*/*C* ratio was increased to ten folds, both conversion and ee decreased. I_2_/48 ratio of 2 produced 100% conversion with the highest 95% ee whether the *S*/*C* ratio is 20 : 1 or 200 : 1. But the I_2_/Ir ratio of 4 : 1 decreased ee to 92% when the *S*/*C* ratio is 20 : 1 and ee was further decreased when *S*/*C* ratio is 200 : 1.

The second method had DCM and MeOH mixed instead of DCM only. 4 : 1 mix of toluene and heptane, only toluene as a solvent for 34m was experimented with of which both had 98% conversion and 99% ee of (*S*)-33m after work-up.

The third method required mixing of 58 and 48 put under 4 cycles of high vacuum (1–2 mbar) and argon (1 bar). The mix is kept under argon, degassed MeOH is added, stirred at 25 °C (RT) for 3 h, solid I_2_ is added, stirred again for 30 min. Under 1 mbar and RT, solvent is removed and dried for 30 min. DCE is added next under argon, solution of 34m is added to the intended solvent, mixed with the above-prepared solution at 5 bar H_2_. When 24 mL of 9 : 2 : 1 toluene : THF : DCE solvent system was used to react 7.5 mmol of 34m at RT, I_2_/48 ratio of 3 : 1, *S*/*C* ratio of 1000 : 1, and the catalyst stirred with MeOH for 1 h; 100% conversion with the highest ee of 97% was found. But when the catalyst was stirred with MeOH for 3 h, no reaction had full conversion except when the catalyst was stored one day after its preparation before being used. In that case, 91 mL of the same ratio of the solvent system was used to react with 38 mmol of 34m at 16 °C with the same I_2_/48 ratio and increased *S*/*C* ratio of 2500. But the ee was reduced to 95.2%. Both the reaction took almost the same time (30 and 29 min). Also, when 119 mL of 13 : 4 : 1 toluene : THF : DCE solvent system was used to react 38 mmol of 34m at 16 °C with the same I_2_/Ir ratio, stirred for 3 h, *S*/*C* ratio of 3000; 98.8% conversion with 95% ee was found after 60 min of reaction time.

There are some technical advantages of this process, compared to the previously known processes-

(1) For the AH of 34m, various chiral catalysts have been tested. It has been found that only the taniaphos catalyst 58 shows a surprisingly high ee of 92–95%; not even the Noyori transfer hydrogenation catalyst.

(2) This novel process of AH does not need the separation of enantiomers through diastereomeric salt formation. And so, the error of recycling the other enantiomer does not occur, which is disadvantageous of the racemic resolution.

(3) Large-scale production shows that the taniaphos catalyst 58 shows a more stable ee in the AH as compared to the Noyori transfer hydrogenation catalyst.

So, 58 catalyst gave rise to (*S*)-configured product.

#### 2.2.14 AH with P-phos ligands and BINAP catalysts

**Fig. 17:**
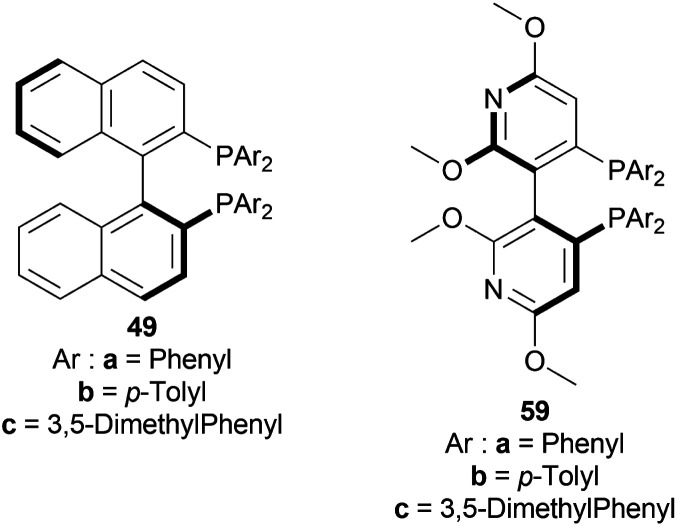
BINAP and P-phos moieties.

**Scheme 25:**
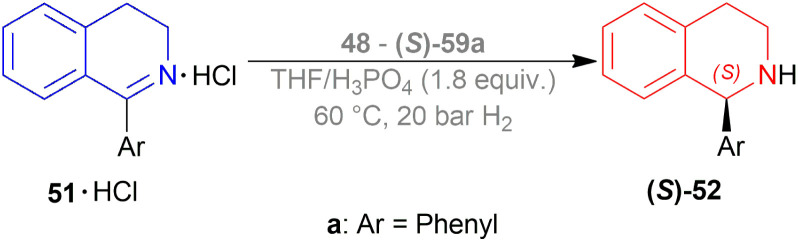
AH with [Ir(COD)Cl]_2_ and (*S*)–P-phos.

Ružič *et al.* tested various BINAP 49 and P-phos 59 moieties (Fig. 17) in DCE (with *S*/*C* of 43 : 1, 50 °C, 30 bar H_2_, 3 h) for screening phosphine ligands with [Ir(COD)Cl]_2_48 (Fig. 8) to reduce HCl salt of 51a to (*S*)-52a (Scheme 25).^49^ Among them (*S*)-BINAP (*S*)-49a, (*R*)-Tol-BINAP (*R*)-49b, (*R*)-Xyl-BINAP (*R*)-49c, (*R*)-P-phos (*R*)-59a, (*S*)-tol-P-phos (S)-59b, (*R*)-xyl-P-phos (*R*)-59c showed good yields (86–98%) but only (*R*)-59a and (*S*)-59b had excellent ee (84% and 78% respectively).

To choose additives, 48 and 49 catalysts in THF (with *S*/*C* of 43 : 1, 50 °C, 30 bar H_2_, 3 h) were experimented with. 0.2 equiv. NaI, 0.2 equiv. MgI_2_, 0.2 equiv. BuN_4_Br had 82–92% yield but low ee (46–62%). Different amounts of H_3_PO_4_ such as 1.2 equiv. (anhydrous), 1.2 equiv. (aq, 85% w/w in water), 2.4 equiv. (anhydrous), 2.4 equiv. (anhydrous) with 0.1 equiv. Potassium iodide (KI) showed full conversion (>97% yield) and ee (82–94%). 12 equiv. CH_3_COOH and 1.2 equiv. (*R*)-1,1-binaphthyl-2,2-diyl hydrogen phosphate also yielded high results (89% and >97% respectively) and ee (78% and 96% respectively).

More tests were done for fine tuning *S*/*C*, temperature, and H_2_ pressure. The use of 48 – (*S*)-59a in THF/H_3_PO_4_ was preferred over other combinations in terms of high % ee values (Scheme 25). Increasing *S*/*C* from 340 : 1 at 50 °C to 425 : 1 at 60 °C increased yield from 97% to full conversion (>97% yield) with the same 95% ee at 30 bar H_2_. And lower reaction pressure reduced yield values. So ultimately, *S*/*C* was increased to 850 : 1 and 1275 : 1 at increased reaction temperatures (60 °C), increased reaction pressure (20 bar H_2_), and longer reaction times (72 h) for optimal results.

So, (*S*)-59a catalyst gave rise to (*S*)-configured product.

#### 2.2.15 AH by chiral spiro iridium phosphoramidite complex

**Fig. 18:**
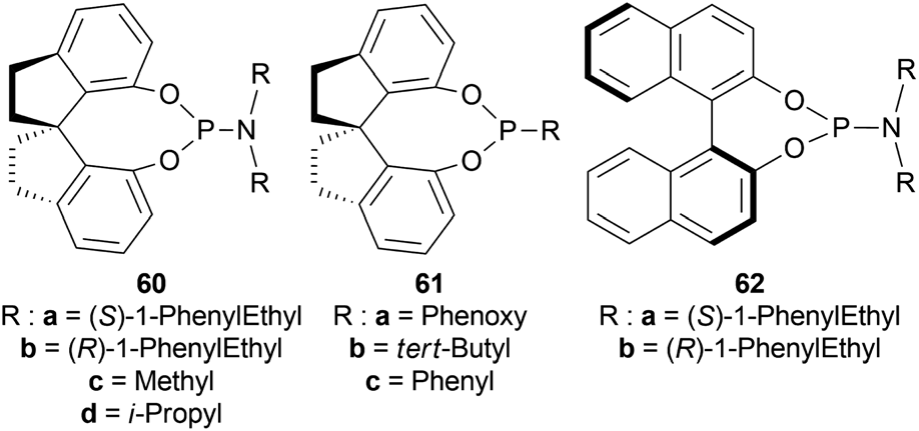
Various chiral spiro iridium phosphoramidite complexes.

**Scheme 26:**
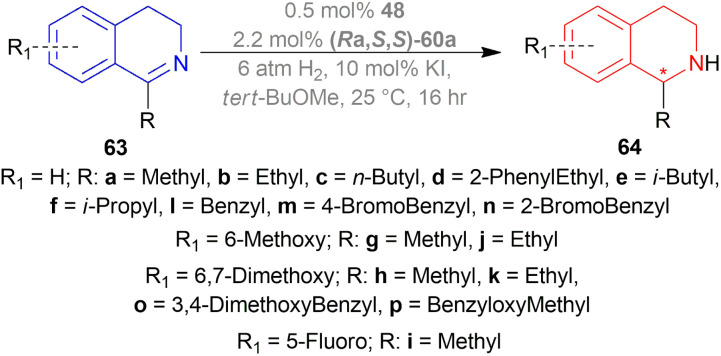
AH with chiral spiro iridium phosphoramidite complex.

Xie *et al.* chose different chiral ligands *e.g.* (*R*a,*S*,*S*)-60a, (*R*a,*R*,*R*)-60b, (*R*)-60c, (*R*)-60d, (*R*)-61a, (*R*)-61b, (*R*)-61c, (*S*a,*S*,*S*)-62a, (*S*a,*R*,*R*)-62b (Fig. 18) for screening to optimally reduce 63a to (*S*)-64a (Scheme 26).^50^

Among the spiro phosphoramidites 60, (*R*a,*S*,*S*)-60a was the best ligand with 100% conversion and 91% ee of (*S*)-64a with THF as the solvent, I_2_ as an additive, and 50 atm H_2_ pressure. Et_2_O and *tert*-BuOMe solvent provided 100% conversion with 99% ee. With *tert*-BuOMe, H_2_ pressure can be reduced to 20 atm without losing conversion or ee. When I_2_ was taken away, only 9% conversion occurred. Lithium iodide and KI as additives also showed 99% ee with 100% conversion. With KI, H_2_ pressure can be reduced to 6 atm without losing conversion or ee.

The spiro phosphite (*R*)-61a and the spiro phosphonites (*R*)-61b, (*R*)-61c had shown good conversion (90–100%) but not very good ee (16–77%). (*S*a,*S*,*S*)-62a and (*S*a,*R*,*R*)-62b ligands also showed 95% conversion with 87% ee of (*S*)-64a and 22% conversion with 82% ee of (*R*)-64a respectively. (*R*a,*S*,*S*)-60a was chosen for later tests.

Under the optimal conditions, (*S*)-64a-(*S*)-64o, (*R*)-64p were hydrogenated from various 1-substituted-DHIQs 63a–63p (Scheme 26). When the substituted part increased in bulk, ee decreased for (*S*)-64a-(*S*)-64f even after H_2_ pressure and/or reaction time increased. Substitution on the benzene ring (with electron-donating two methoxy groups in (*S*)-64h and (*S*)-64k, or an electron-withdrawing fluoride in (*S*)-64i) of THIQs decreased ee (91–94%) with 93–96% conversion. 1-Benzyl-DHIQs 63l–63o were hydrogenated to (*S*)-64l – (*S*)-64o with high ee (96–97%) and 88–96% conversion. 1-Benzyloxymethyl-DHIQ 63p was also hydrogenated with 95% ee and 94% conversion to produce (*R*)-64p.

**Scheme 27:**
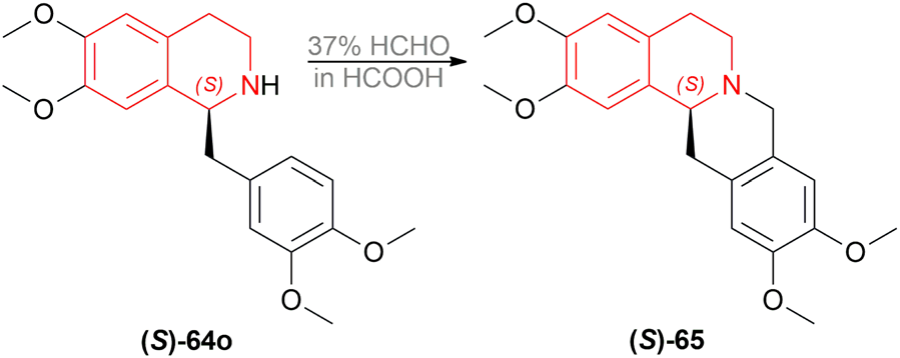
Synthesis of (*S*)-xylopinine.

So, (*R*a,*S*,*S*)-60a catalyst gave rise to (*S*)-configured products except for 63p, (*R*)-configured product was found.

(*S*)-64o was prepared by the optimum reaction condition with 88% yield and 96% ee; which was reacted with 37% HCHO in HCOOH for 2 h at 90 °C to yield (*S*)-xylopinine (*S*)-65 (85%) with the same 96% ee (Scheme 27).

#### 2.2.16 Catalytic asymmetric reduction using Noyori catalyst

**Fig. 19:**
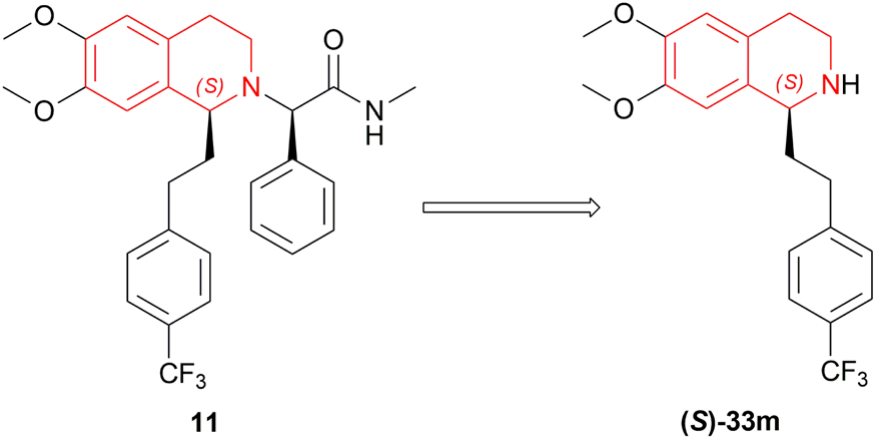
Almorexant and its intermediate.

Almorexant 11 is an efficient antagonist for orexin receptors which was active on both orexin OX_1_ and OX_2_ receptors.^51^ Verzijl *et al.* prepared 11 by asymmetric reduction of 34m to the key intermediate (*S*)-33m (Fig. 19).^52^

**Scheme 28:**
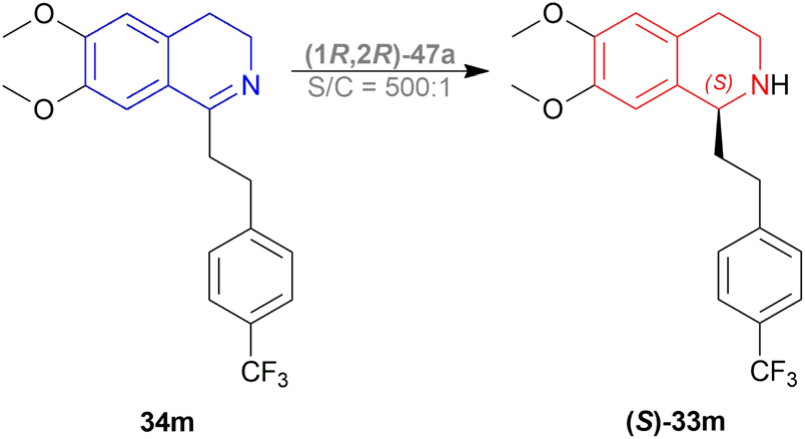
Synthesis of the almorexant intermediate.

On a 250–300 g scale using (1*R*,2*R*)-47a (Fig. 12), 11 was obtained in high yield (95%) with good ee (81–95%) at *S*/*C* of 500 : 1 (Scheme 28). In MeOH, HCl salt of the product was found up to 99% ee. And during a 30 kg production campaign, the product yield dropped to 57–60% and ee also dropped to 76–80%; because of the *N*-formylated THIQ, a by-product.

So, (1*R*,2*R*)-47a catalyst gave rise to (*S*)-configured product.

#### 2.2.17 Ruthenium-catalyzed ATH of 1-aryl DHIQs

**Fig. 20:**
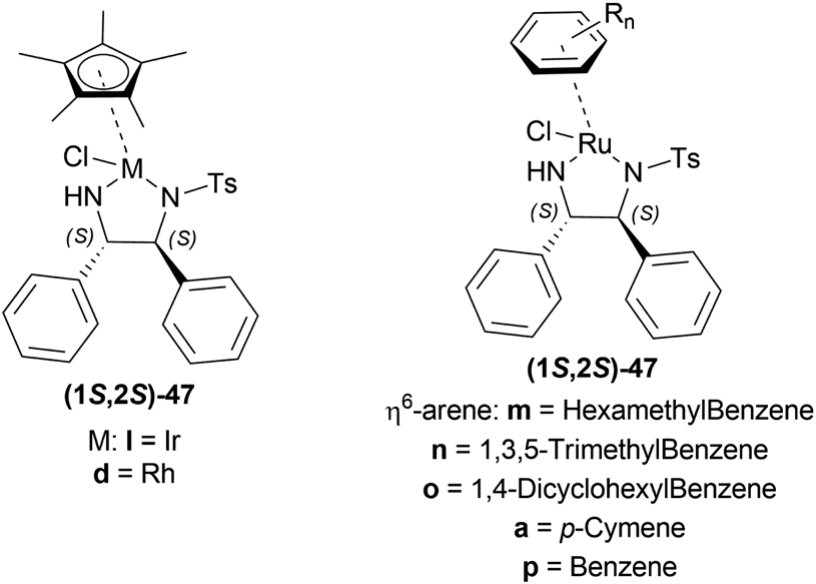
TsDPEN complexes.

Wu *et al.* screened for ATH of 1-phenyl-DHIQ 51r in DCM at 30 °C with a 5 : 2 azeotropic mixture of HCOOH–Et_3_N and TsDPEN complexes 47 (Fig. 20) for 16 h.^53^ Cp*Ir(iii)-TsDPEN (1*S*,2*S*)-47l or Cp*Rh(iii)-TsDPEN (1*S*,2*S*)-47d gave 100% conversion with racemic 52r. Conversion declined significantly for (1*S*,2*S*)-47m–(1*S*,2*S*)-47p and (1*S*,2*S*)-47a, but (1*S*,2*S*)-47p had 100% conversion with enantiomeric ratio (er) of 87.5 : 12.5 (75% ee). Then different solvents were experimented with instead of DCM and 100% conversion was found for each of them only varying in er. ACN, DMF, dioxane, MeOH, THF, toluene, IPA gradually increased er from 72.5 : 27.5 (45% ee) to 91 : 9 (82% ee). For IPA, reaction at 50 °C only decreased er to 86 : 14 (72% ee) and reaction at 0 °C decreased conversion to 30% while er was 90.5 : 9.5 (81% ee).

Then, 51r–51q′ were tested under optimum conditions which showed 72–97% yields of 52r–52q′ with 90.5 : 9.5–99.5 : 0.5 er (81–99% ee). Methyl, bromide, and chloride substitution at the *meta* position of the 1-phenyl moiety of 6,7-dimethoxy-THIQs 52s–52u had similar ee (82–84%) as 52r (82%) but higher yields were found (90–93% *vs.* 82%). Methoxy, fluoride, and chloride substitution at the para position of the 1-phenyl moiety of 6,7-dimethoxy-THIQs 52v–52x had not only higher ee (87–91%) but also higher yields (91–94%). Electron-withdrawing or electron-donating groups at the *ortho* position of the 1-phenyl moiety of 6,7-dimethoxy-THIQs 52y–52g′ had also excellent ee (96–99%) and higher yields (83–97%). These results represent that when the bulkiness of the *ortho*-position substituents increases, enantioselectivity also gradually increases.

**Scheme 29:**
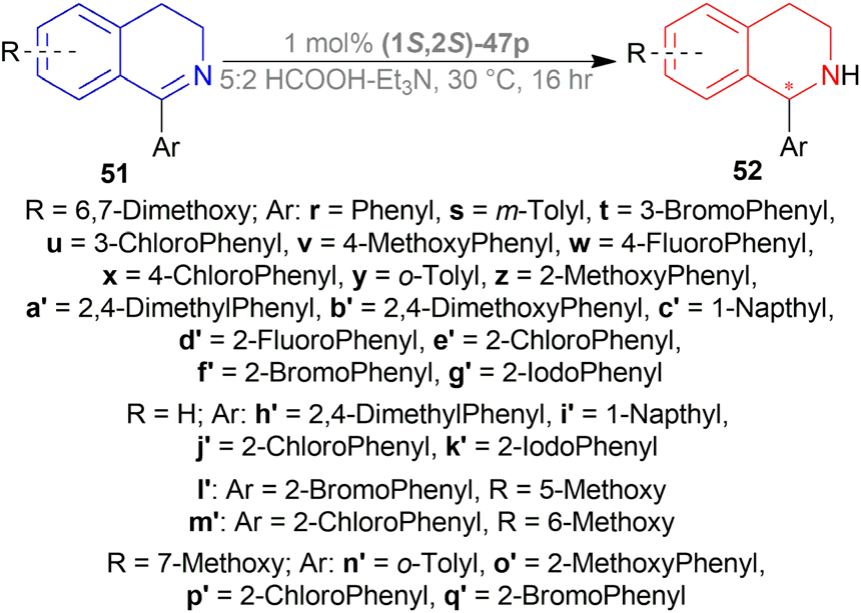
ATH with TsDPEN complexes.

On a different note, THIQs bearing no substituents, 52h′–52k′ had lower yields (72–75%) and lower ee (90–92%) than similar 6,7-dimethoxy-THIQs 52a′, 52c′, 52e′, 52g′ even after 40 h reaction time. Again, in the case of 51l′–51q′ having one methoxy at 5, 6, or 7 position of DHIQ core, reaction results are less affected for electron-withdrawing groups bearing substrates (51e′, 51f′*vs.*51l′, 51m′, 51p′, 51q′) than for electron-donating groups bearing substrates (51y, 51z*vs.*51n′, 51o′), producing 52l′–6q′ in high yields (86–90%) and 97.5 : 2.5–98 : 2 er (95–96% ee) (Scheme 29).

**Scheme 30:**
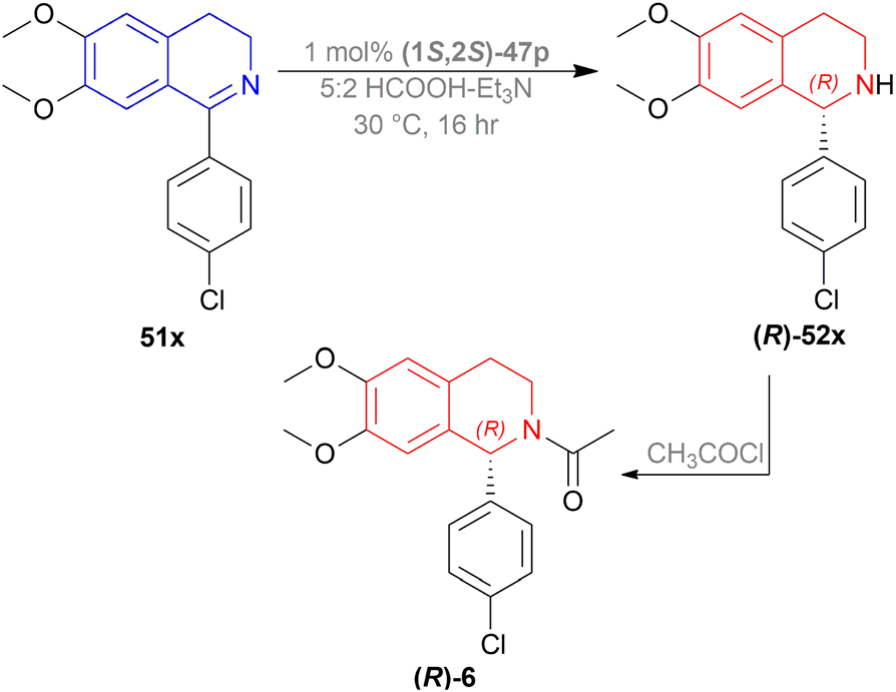
Synthesis of an AMPA receptor antagonist.

A gram synthesis of compound 6, an AMPA receptor antagonist, was done *via*51x by optimum ATH conditions having 93% yield and 93.5 : 6.5 er (87% ee). When recrystallized in MeOH, the yield was 80% and had an ee of 98% (Scheme 30).

#### 2.2.18 Ruthenium-catalyzed AH of 1-alkyl DHIQs in ionic liquid

**Scheme 31:**
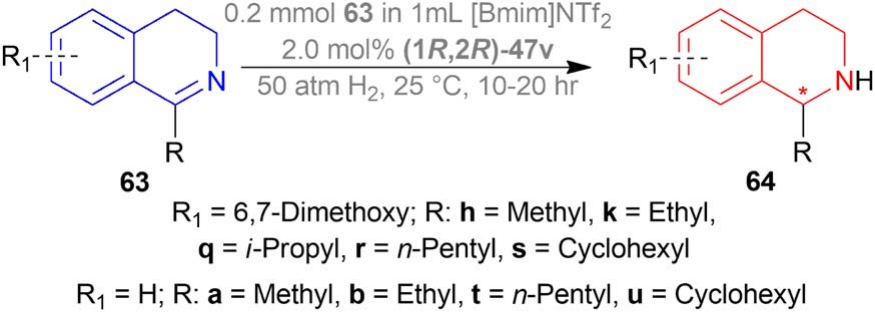
AH of 1-alkyl-DHIQs.

Ding *et al.* investigated AH reactions of 1-alkyl-DHIQs 63 (Scheme 31) using different chiral cationic Ru complex as catalysts in imidazolium ionic liquids.^54^

**Fig. 21:**
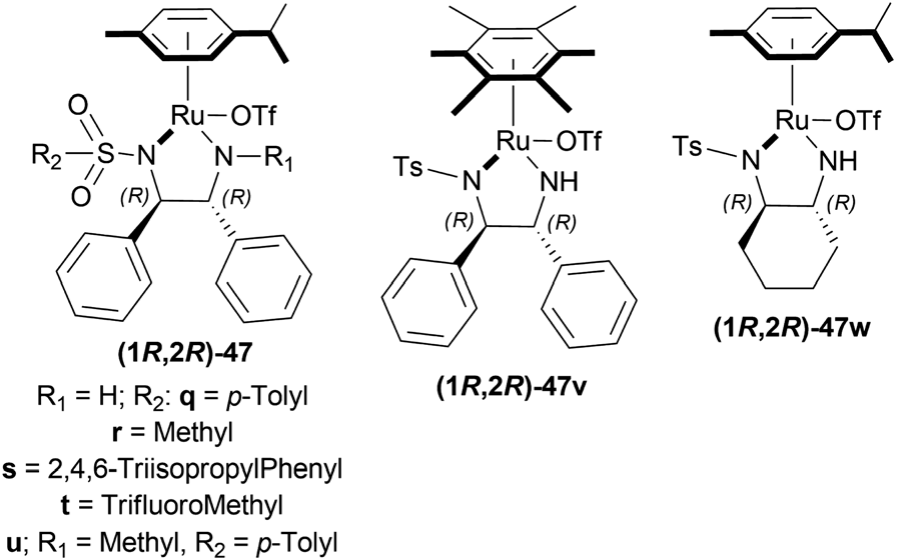
Chiral cationic Ru complexes.

Hydrogenation of 6,7-dimethoxy-1-methyl-DHIQ 63h in the presence of (1*R*,2*R*)-47q (Fig. 21) in 1-*n*-butyl-3-methyl-imidazolium hexafluorophosphate or [Bmim]PF_6_ afforded the (*S*)-6,7-dimethoxy-1-methyl-THIQ (*S*)-64h with 100% conversion with an ee of 96%. Then, hydrogenation of 63h in [Bmim]SbF_6_ showed 95% ee. The highest ee of 98% was observed in [Bmim]NTf_2_, which was better than that in MeOH (96% ee). So [Bmim]NTf_2_ was selected as the solvent for the AH of 1-alkyl-DHIQs 63.

After that, (1*R*,2*R*)-47q-(1*R*,2*R*)-47w (Fig. 21) catalysts were screened at 2.0 mol%. The *N*-(*p*-toluenesulfonyl)-1,2-diphenylethylenediamine or TsDPEN ligand (1*R*,2*R*)-47u gave the best result (100% conversion, 99% ee). The *N*-(*p*-toluenesulfonyl)-1,2-cyclohexanediamine or TsCYDN ligand (1*R*,2*R*)-47w showed a much lower conversion of 78% and a lower ee of 75%. So, (1*R*,2*R*)-47u was the best choice of catalyst. Also, low temperature (0 °C) or low hydrogen pressure (20 atm) resulted in lower conversions of 70 and 83%. 1.0 mol% catalyst also afforded a lower conversion of 75% with 97% ee.

At 25 °C and 50 atm H_2_, various 1-alkyl-DHIQs 63 was hydrogenated in [Bmim]NTf_2_ with 2.0 mol% (1*R*,2*R*)-47v (Scheme 31). All had excellent 93–99% ee. Compared to 63h, substrates having more steric (*i*-propyl, cyclohexyl) or longer (ethyl, *n*-pentyl) side chains gave slightly lower ee of 97%, 93%, 98%, 97% for 63k, 63q, 63r, 63s respectively. Hydrogenation of DHIQs with no 6,7-dimethoxy substitutions bearing ethyl or cyclohexyl side chain had a slight decrease in % ee (96 and 94% for 63b and 63u; compared to 63k and 63s).

So, (1*S*,2*S*)-47v catalyst gave rise to (*S*)-configured products for 63h, 63k, 63s, 63b, 63t and (*R*)-configured products for 63q, 63r, 63a, 63u.

#### 2.2.19 ATH in water varying molar ratio of HCOOH and NEt_3_

Shende *et al.* screened molar ratio of HCOOH and NEt_3_ for ATH of 6,7-dimethoxy-1-methyl-DHIQ 34a with (1*S*,2*S*)-47d (Fig. 10) at 40 °C.^55^ The azeotropic mixture of 2.5 : 1 molar ratio of HCOOH and NEt_3_ in DCM (pH 5) had 99% conversion and 89% ee in 10 min. While the same mixture in water (pH 3) took 24 h to reach 99% conversion with just 2% ee. But a molar ratio of 1.1 : 1 of HCOOH and NEt_3_ in water (pH 5.1) needed 10 min for full conversion. So, 1.1 : 1 molar ratio was chosen for later tests.

**Scheme 32:**
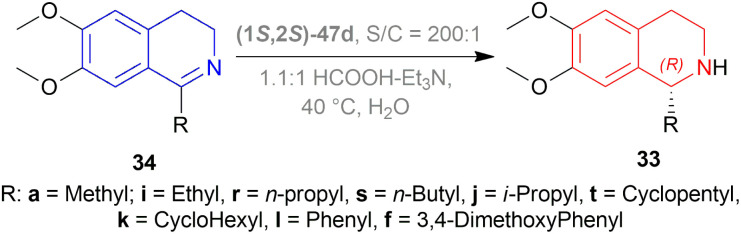
ATH in water with 1.1 : 1 molar ratio of HCOOH and NEt_3_.

Then 34a, 34f, 34i–34l, 34r–34t were reduced with (1*S*,2*S*)-47d, *S*/*C* = 200 : 1, HCOOH : NEt_3_ = 1.1 : 1, 40 °C in water (Scheme 32). 6,7-dimethoxy-1-alkyl-THIQs (*R*)-33a, (*R*)-33i-(*R*)-33k, (*R*)-33r-(*R*)-33t had 95–98% yield with 83–99% ee in 6 min. But 1-aryl-DHIQs had lower yields and % ee. (*R*)-33l and (*R*)-33f had 87 and 29% yield with 5 and 2% ee respectively.

Here, (1*S*,2*S*)-47d gave rise to (*R*)-configured products.

#### 2.2.20 Ru-Catalyzed ATH of 1-aryl-DHIQs

**Scheme 33:**
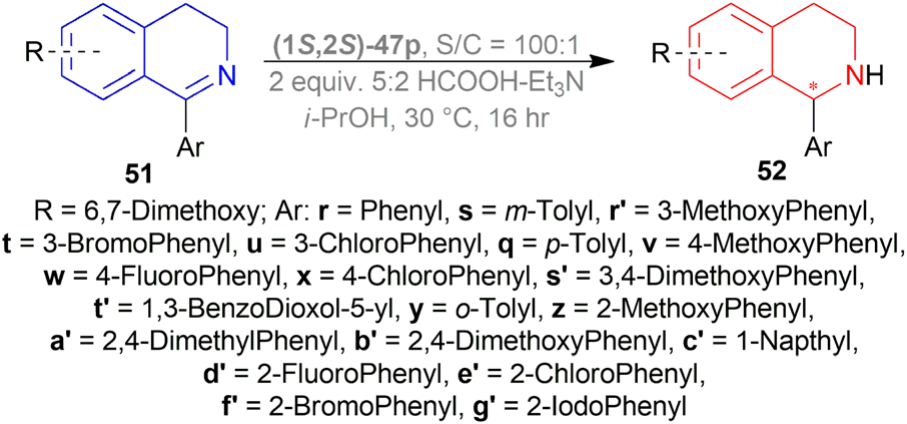
ATH of 1-aryl-6,7-dimethoxy-DHIQs.

Perez *et al.* optimized the ATH of 1-phenyl-6,7-dimethoxy-DHIQ 51r to form 1-phenyl-6,7-dimethoxy-THIQ 52r (Scheme 33).^56^ They experimented with 1 mol% of 14 metal-based diamine complexes at 30 °C in DCM for 16 h using a 5 : 2 HCOOH–Et_3_N azeotropic mixture as a hydrogen source. Among the complexes, (1*S*,2*S*)-47p (Fig. 20) (with 100% yield and 74% ee) was selected for later optimization.

THF, toluene, and dioxane (less polar solvents) yielded products with a higher ee of 65–70% than ACN and DMF (polar aprotic solvents) of 45–48% ee. But when IPA was used, the best yield of 90% and ee of 82% was achieved. Decreasing the temperature from 30 °C to 20 or 10 °C, the yield or ee did not fluctuate that much. Decreasing the temperature to 0 or −10 °C reduced yield dramatically to 27–28% while maintaining ee to 80%. Again, increasing the temperature to 50 °C provided a yield of 87% but decreased ee to 72%. When catalyst loading was lowered to 0.5 mol% at 30 °C for 30 h, yield decreased slightly to 83% while ee was almost the same (81%). So, optimal conditions for ATH of 1-aryl-6,7-dimethoxy-DHIQs 51q–51g′, 51r′–51t′ were as following: 1 mol% of catalyst (1*S*,2*S*)-47p, IPA as solvent, 5 : 2 HCOOH–Et_3_N azeotropic mixture as H_2_ source, 30 °C for 16 h (Scheme 33).

In 51s–51u, where there are electron-withdrawing or electron-donating substituents on the *meta* position, yield and ee were 90–93% and 82–84%, almost the same as 51r. Except in 51r′, the methoxy group provides such steric and electronic effects that reduce yield to 83% and ee to 69%.

Para-substituted DHIQ 51q, 51v–51x were reduced with a similar range of good yields (90–92%) and ee (87–91%). *Meta*- and *para*-disubstituted DHIQs 51s′ and 51t′ had a drop in both yield (85 and 87%) and ee (75 and 82%). *Ortho*-substituted DHIQs 51y–51g′ had excellent yields (83–97%) and ee (96–99%) irrespective of having electron-donating or withdrawing groups. These results are proof that steric effects are more responsible than the electronic effects. 51d′–51g′ had a fluoride, a chloride, a bromide, and an iodide on the *ortho*-position and as the atom size of the halogen increases, ee also increased from 96% to 99%.

**Scheme 34:**
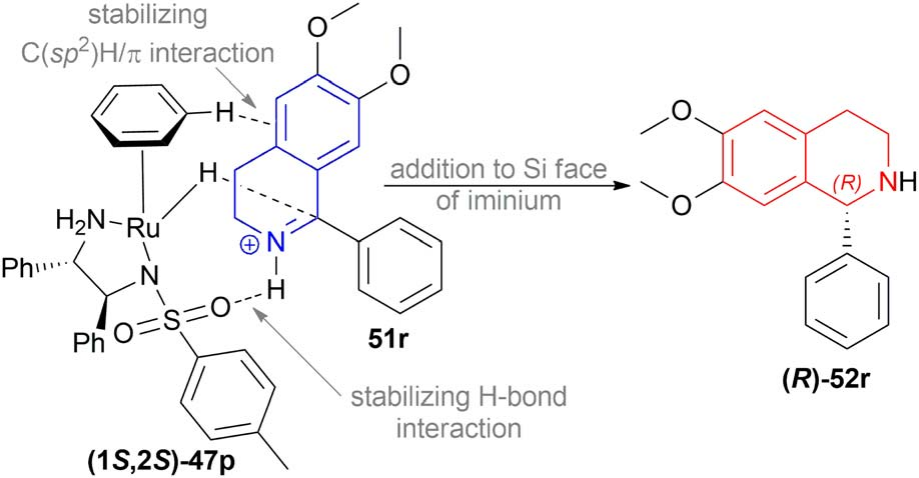
Reduction using Ru(ii)-TsDPEN catalyst.

Then, 1-aryl-DHIQs 51a–51h, 51u′–51x′ were reduced using Ru(ii)-TsDPEN catalyst under the optimized conditions (Scheme 33). 51a was reduced with a high yield of 90% but a low ee of 29% than 51r. This may be due to the fact that the two methoxy groups in 51r donate electron that increases the C=N bond electron density. Thus, stronger C(sp_2_)H/π interactions between a hydrogen atom on the η^6^-benzene ligand and the aromatic ring of the isoquinoline skeleton are possible (Scheme 34).

**Scheme 35:**
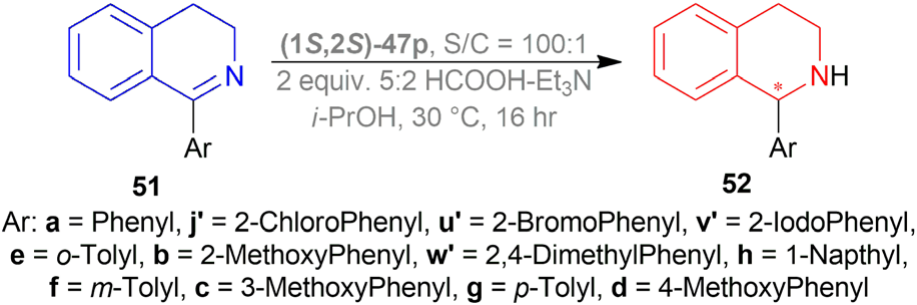
ATH of 1-aryl-DHIQs.

*Ortho*-substituted 1-aryl-DHIQs 51b, 51e, 51h, 51j′, 51u′–51w′ had lower yields (71–78%) and ee (79–94%) than *ortho*-substituted 1-aryl-6,7-dimethoxy-DHIQs 51y–51g′. *Meta*- and *para*-disubstituted 1-aryl-DHIQs 51c, 51d, 51f, 51g had higher yield (87–93%) but lower ee (33–39%) than *meta*- and *para*-disubstituted 1-aryl-6,7-dimethoxy-DHIQs 51s′ and 51t′ (Scheme 35).

**Scheme 36:**
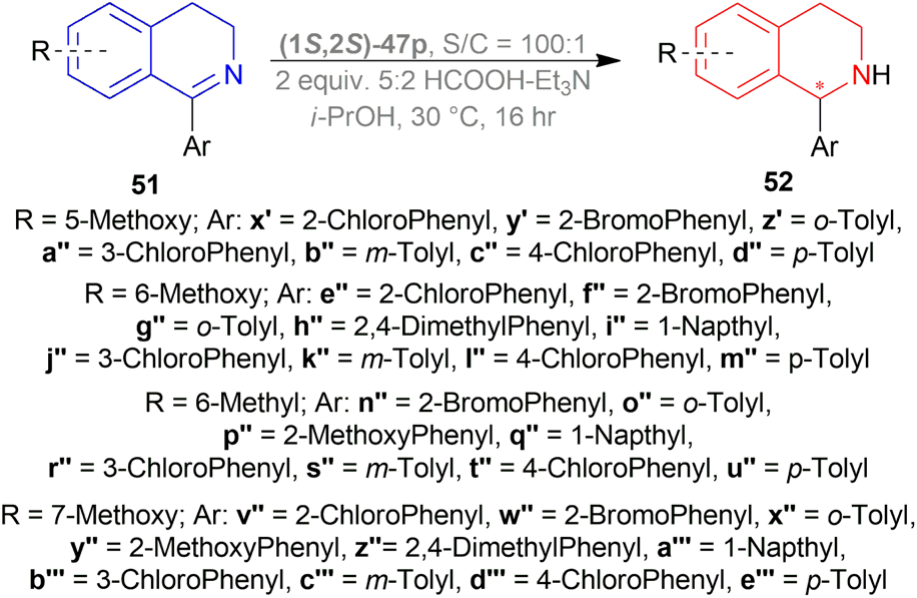
ATH of 1-aryl-5-methoxy-DHIQs, 1-aryl-6-methoxy-DHIQs, and 1-aryl-7-methoxy-DHIQs.

1-Aryl-5-methoxy-DHIQs 51x′–51d′′, 1-aryl-6-methoxy-DHIQs 51e′′–51m′′, 1-aryl-6-methyl-DHIQs 51n′′–51u′′, 1-aryl-7-methoxy-DHIQs 51v′′–51e′′′ (Scheme 36) were also reduced by ATH to outline the effects of electron-rich substituents present on the isoquinoline core.

*Ortho*-substituted DHIQs 51x′–51z′, 51e′′–51i′′, 51n′′–51q′′, and 51v′′–51a′′′ had good ee (84–95%, 91–96%, 79–93%, and 92–96% respectively). This confirms that the *ortho*-substitution on the 1-phenyl ring is important for achieving high levels of selectivity, as also seen for 1-aryl-6,7-dimethoxy-DHIQs 51y–51g′ (Scheme 33) and 1-aryl-DHIQs 51b, 51e, 51h, 51j′, 51u′–51w′ (Scheme 35), irrespective of the substitution pattern on the benzene ring of the isoquinoline core.

51a′′–51d′′ having a methoxy group at the 5-position, were reduced with ee of 3–34%. 51j′′–51m′′and 51r′′–51u′′, bearing a methyl or a methoxy group at the 6-position, were reduced with ee from 33–60% and 27–53% respectively. 51b′′′–51e′′′ having a methoxy group at the 7-position, were reduced with a higher ee of 54–80% (Scheme 36).

Rather than electronic effects, the catalytic efficiency of the process for monosubstituted DHIQs 51x′–51e′′′ (Scheme 36) was due to the increased steric hindrance near the reactive center during the approach of the Ru catalyst.

**Scheme 37:**
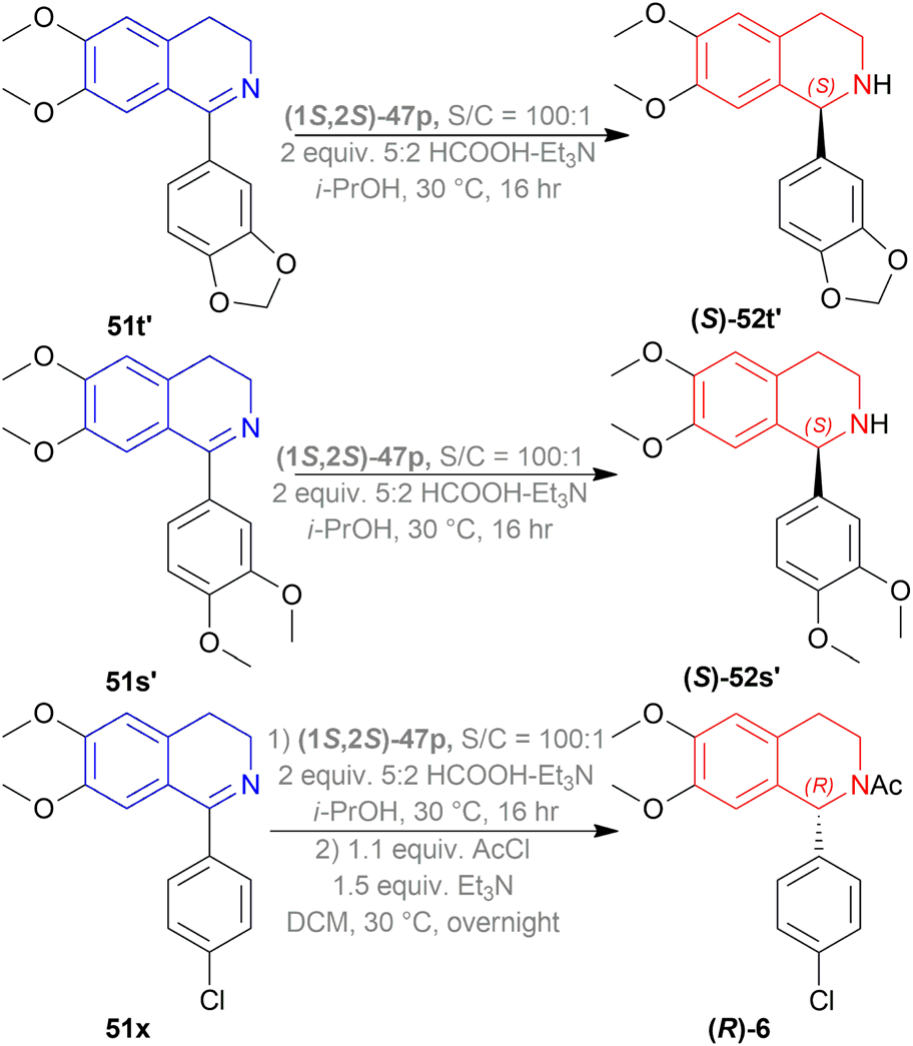
Synthesis of (S)-norcryptostyline I, (S)-norcryptostyline II, and an AMPA receptor antagonist.

51t′, 51s′, 51x were used to synthesize (*S*)-52t′ or (*S*)-norcryptostyline I (87% yield, 82% ee), (*S*)-52s′ or (*S*)-norcryptostyline II (85% yield, 75% ee) and AMPA receptor antagonist 6 (80% yield, 98% ee) respectively (Scheme 37).

#### 2.2.21 Ir-catalysed hydrogenation of DHIQ hydrochlorides with P-trifluoromethyl ligands derived from Josiphos

**Fig. 22:**
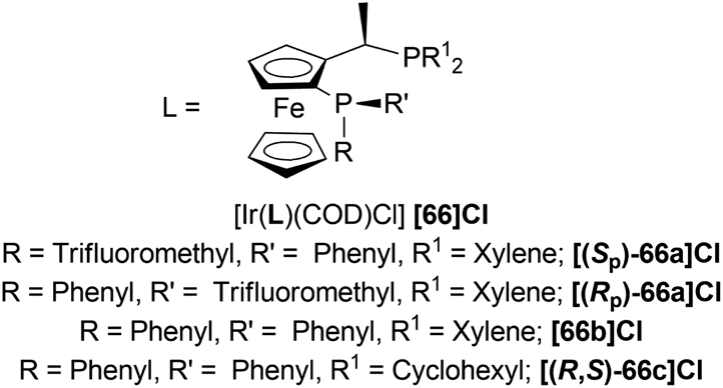
P-Trifluoromethyl ligands.

**Scheme 38:**
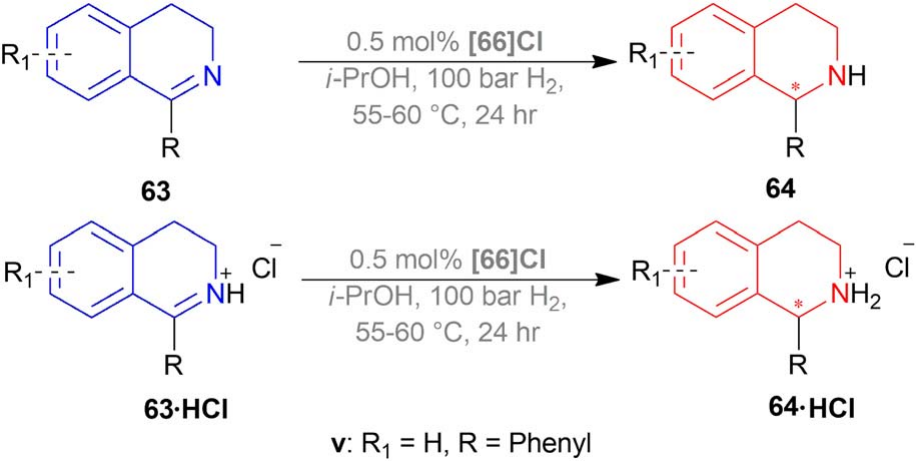
AH of DHIQ hydrochlorides with P-trifluoromethyl ligands and by Ir-catalyst.

Schwenk and Togni reduced 1-phenyl-DHIQ 63v with [(*S*_P_)-66a]Cl, [(*R*_P_)-66a]Cl, [66b]Cl, and [(*R*,*S*)-66c]Cl (Fig. 22).^57^ [(*S*_P_)-66a]Cl and [(*R*_P_)-66a]Cl (Fig. 22) both yielded poorly (25% and 18% respectively) and ee values were also low (30% and 16% respectively). [66b]Cl yielded more than them (75%) but ee value did not increase (21%). When 63v was activated by protonation, 63v·HCl gave more yield than free 63v. (*R*)-1-[(*S*)-2-(DiphenylPhosphino)-ferrocenyl]EthylDicyclohexylPhosphine or (*R*,*S*)-josiphos [(*R*,*S*)-66c]Cl catalyzed 63v·HCl poorly (yield 60%, ee 30%) but (*S*)–P-phos (*S*)-59a (Fig. 17) gave high yield (>99%) and ee value (97%). While [(*R*_P_)-66a]Cl had low enantioselectivity (23% ee), [(*S*_P_)-66a]Cl gave a satisfactory ee of 96%. These two catalysts also had >99% yield without the use of any additives (Scheme 38).

**Scheme 39:**
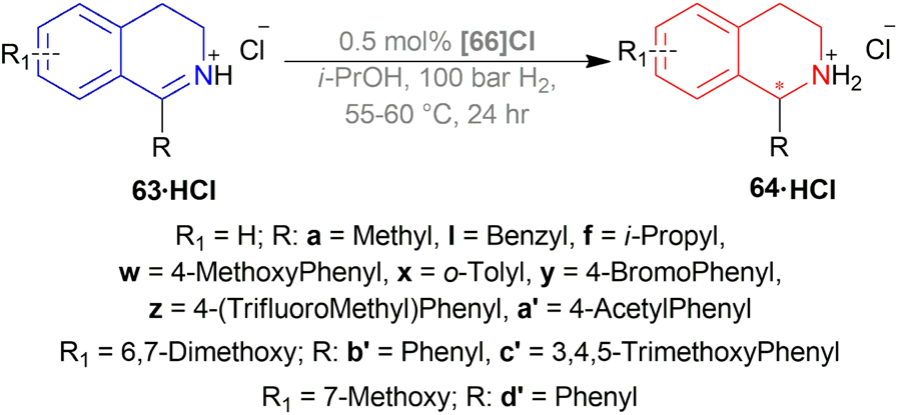
AH of DHIQ hydrochlorides with P-trifluoromethyl ligands and by Ir-catalyst.

Then, 63a·HCl, 63l·HCl, 63f·HCl, 63v·HCl-63d′·HCl were reduced with [(*S*_P_)-66a]Cl and [66b]Cl catalysts. Having steric bulk moieties in position 1, 64v·HCl and 64f·HCl (ee of 96% and 84%; 93% and 85% respectively) had higher ee than 64a and 64l (ee of 28% and 50%; 35% and 38% respectively). Again, electron-rich moieties in position 1 lowered ee in 64w, 64b′, 64c′, and 64d′ (ee of 89% and 90%; 34% and 46%, 57% and 33%; 75% and 77% respectively) (Scheme 39).

(*S*_P_)-66a is the relatively electron-poor ligand, so [(*S*_P_)-66a]Cl produced 64v·HCl of 96% ee than [(*S*_P_)-66a]I (64v·HI of 93% ee). On the other hand, 66b is an electron-rich ligand. For this reason, [66b]I afforded 64v·HI of 93% ee than [66b]Cl (64v·HCl of 84% ee).

#### 2.2.22 AH of DHIQs by Noyori – Ikariya half – sandwich complexes and their analogs

**Fig. 23:**
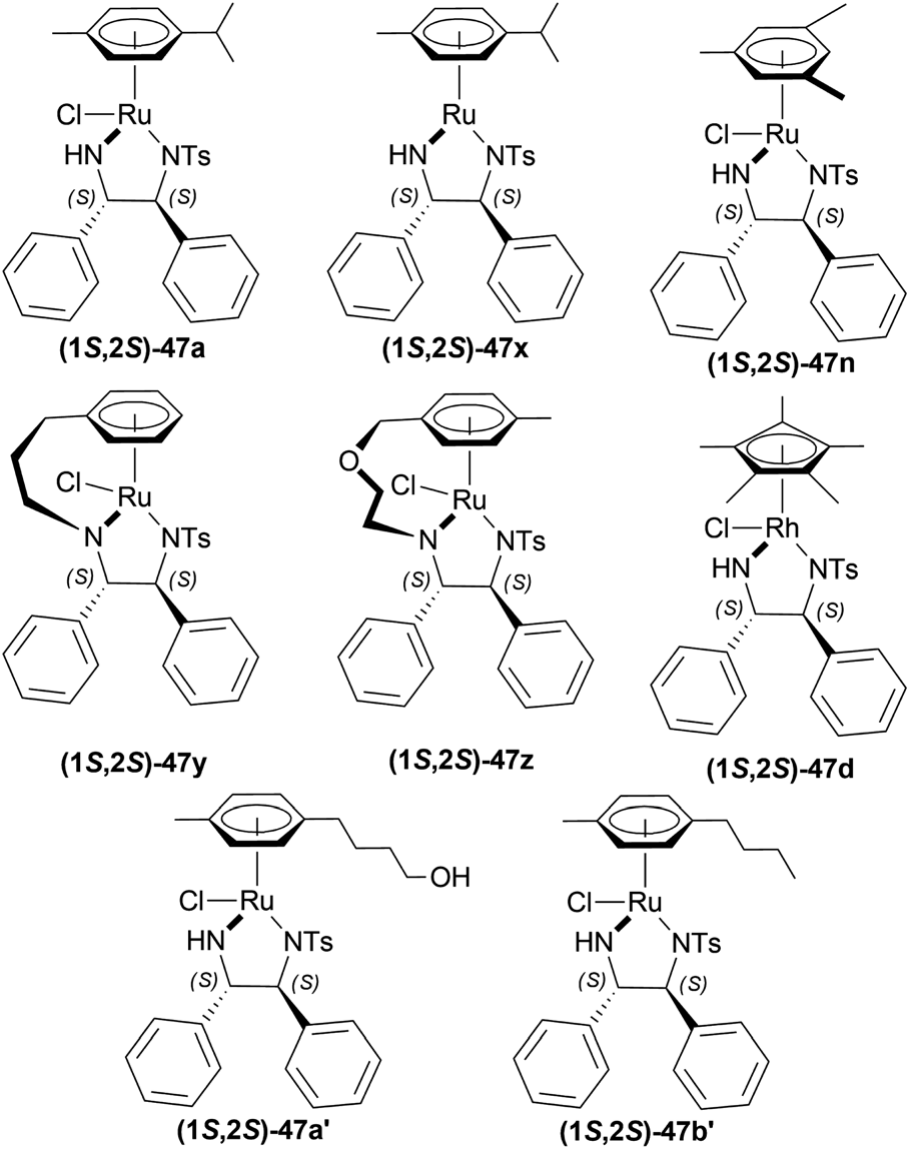
Noyori – Ikariya half – sandwich complexes and their analogs.

**Scheme 40:**
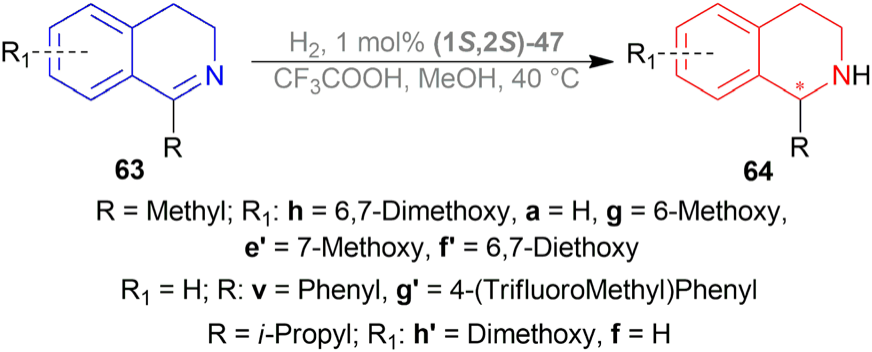
AH of 1-alkyl-DHIQs.

Vilhanová *et al.* screened solvents for AH of 6,7-dimethoxy-1-methyl-DHIQ 63h with (1*S*,2*S*)-47a (Fig. 23) (Scheme 40).^58^ ACN did not even start the reaction and DMSO, MeOH had a very low conversion rate (2–4% and 6–10% respectively). To undergo reduction, the imine needed to be activated by the polarization of the C=N bond. For this, an acid was required in MeOH. 1 equiv. Trifluoromethanesulfonic acid had no effect of its own, showing a 9% conversion. While 1 equiv. of HBF_4_ (48% in water), and trifluoroacetic acid increased conversion to 19% and 57% separately. When the temperature was increased to 40 °C, the conversion was satisfactory (96%). But then it was found that the order of addition of the mixture was critical as the substrate had to be protonated first as follows: substrate, MeOH, acid, and then catalyst. In these optimal conditions, substrates 63a, 63f–63h, 63v, 63e′–63h′ (Scheme 40) were catalyzed with (1*S*,2*S*)-47a, (1*S*,2*S*)-47d, (1*S*,2*S*)-47n, (1*S*,2*S*)-47x-(1S,2S)-47b′ respectively (Fig. 23).

63a, 63g, 63h, 63e′ showed full conversion (>99%) with (1*S*,2*S*)-47a (87–96% ee), (1*S*,2*S*)-47x (85–96% ee), (1*S*,2*S*)-47n (81–96% ee), and (1*S*,2*S*)-47d (75–90% ee). (1*S*,2*S*)-47a and (1*S*,2*S*)-47x had similar results which meant that the auto-dissociation of the Ru–Cl bond of (1*S*,2*S*)-47a is not a rate-limiting step. With (1*S*,2*S*)-47y, 63h, 63a, 63e′ had also full conversion (>99%) except 63g (96%) with 59–78% ee. (1*S*,2*S*)-47z had lower conversion for 63h (44%; 94% ee) and 63g (84%; 90% ee) than 63a and 63e′ (>99% for both; 84% and 91% ee respectively) (Scheme 40).

63f′ and 63v, which are 1-aryl-DHIQs, had full conversion (>99%) only with (1*S*,2*S*)-47d having 7% and 9% ee respectively. So, it is clear that 1-aryl-DHIQs require more reactive and different catalysts.

63g′ and 63h′, the 6,7-dialkoxy-DHIQs had high conversion except for (1*S*,2*S*)-47y (70% and 90% conversion; 83–96% ee) and (1*S*,2*S*)-47z (31% and 27% conversion; 95–98% ee). 63h′ and 63f both are 1-*i*-propyl-DHIQs but 63f only showed full conversion (>99%) for (1*S*,2*S*)-47y (66% ee).

(1S,2S)-47a′ and (1*S*,2*S*)-47b′ also showed similar or larger conversion and ee values compared to (1*S*,2*S*)-47a, (1*S*,2*S*)-47n, (1*S*,2*S*)-47x – (1*S*,2*S*)-47z. Greater conversion was found for substrate 63f (88% and >99% conversion respectively) which was noteworthy (Scheme 40).

For ATH, (1*S*,2*S*)-47a, (1*S*,2*S*)-47a′ and (1*S*,2*S*)-47b′ showed good ee values for 63a, 63g, 63h, 63e′, 63h′ (>85%). And again, (1*S*,2*S*)-47a′ and (1*S*,2*S*)-47b′ had large ee values (70% and 76% respectively) than (1*S*,2*S*)-47a (50%). It was suggested that (1*S*,2*S*)-47a followed first-order kinetics, and (1*S*,2*S*)-47a′, (1*S*,2*S*)-47b′ showed zero-order kinetics for ATH.

#### 2.2.23 ATH of 1-aryl-DHIQs using a Cp*Ir(TsDPEN) complex

**Fig. 24:**
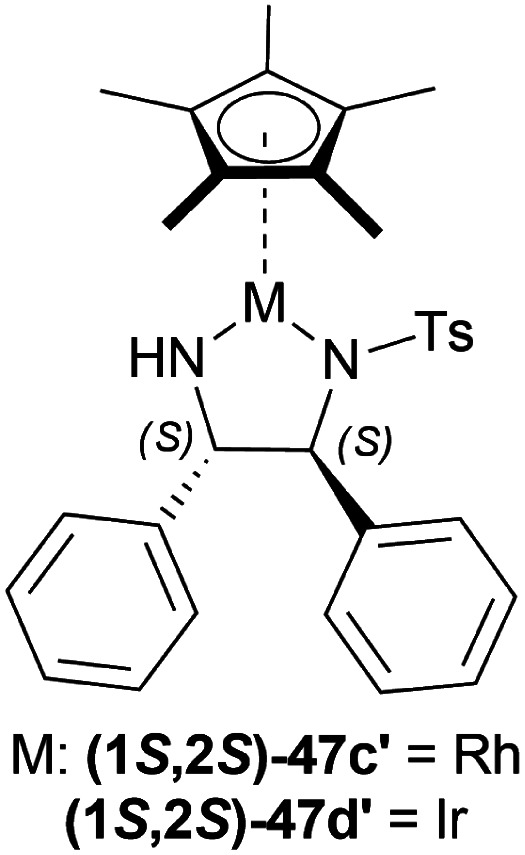
Cp*Rh(TsDPEN) and Cp*Ir(TsDPEN) complex.

**Scheme 41:**
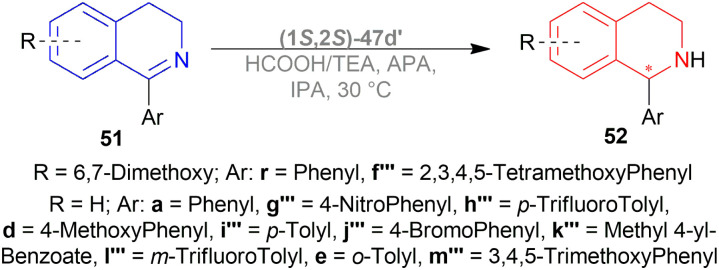
ATH of 1-aryl-DHIQs.

Vilhanová *et al.* observed (1*S*,2*S*)-47d (Fig. 23), (1*S*,2*S*)-47c′, (1*S*,2*S*)-47d′ (Fig. 24) complexes in ACN, IPA, DCM, DMSO, 1,1,1,3,3,3-hexafluoro-2-propanol (HFIP), HCOOH/TEA hydrogen-donor solvents for 24 h but no combinations gave full conversion to 52a from 51a (Scheme 41).^59^ ACN gave a very low conversion of 2–3% with (1*S*,2*S*)-47d, (1*S*,2*S*)-47c′ but a good conversion of 87% with (1*S*,2*S*)-47d′ (10% ee). IPA had an increasing conversion rate with the complexes (64%, 66%, and 83% respectively) but moderate ee (60%) with (1*S*,2*S*)-47d′ only. Reaction without TEA had a lower conversion of 54% (34% ee) and without HCOOH or both had almost no conversion at all. DCM had moderate conversion (57%, 7% ee) but DMSO and HFIP had no reaction done. HCOOH/TEA mixture at a molar ratio of 2.5 : 1 had 80% conversion with 71% ee while at 1 : 1 had 72% conversion with 77% ee. A 1 : 1 molar ratio was selected for further tests.

Several additives were also experimented on. Among them, 85% aq. solution of orthophosphoric acid increased the ee to 82%. Anhydrous Phosphoric Acid (APA) increased ee to 86% at >99% conversion with anhydrous IPA as a solvent and (1*S*,2*S*)-47d′ as complex.

But without APA, conversion lowered to 65% with lower ee also (77%). Without HCOOH, TEA, or both, the reaction would not proceed. (1*S*,2*S*)-47a, (1*S*,2*S*)-47x, (1*S*,2*S*)-47d (Fig. 23), (1*S*,2*S*)-47c′ (Fig. 24) also resulted in low conversion.

According to the optimum conditions, 1-phenyl-DHIQ 51a had full conversion (>99%) with 86% ee in 3 h while 6,7-dimethoxy-1-phenyl-DHIQ 51r had 88% conversion with 72% ee. The high conversion of 51d, 51g′′′–51k′′′ was not altered to a significant extent even after changing the *para*-substitution of the 1-phenyl moiety. For 51l′′′ and 51e, trifluoromethyl substitution in the *meta*-position of the 1-phenyl moiety decreased catalytic activity (93% conversion in 18 h) and methyl substitution in *ortho*-position had poor reactivity (55% conversion). 51m′′′ having the 3,4,5-trimethoxyphenyl group needed 6 h to gain 96% conversion. As 51r, highly methoxylated 51f′′′ showed a decrease in conversion (only 46%). Common features of these two substrates may be the reason for inhibiting ATH under these conditions (Scheme 41).

**Scheme 42:**
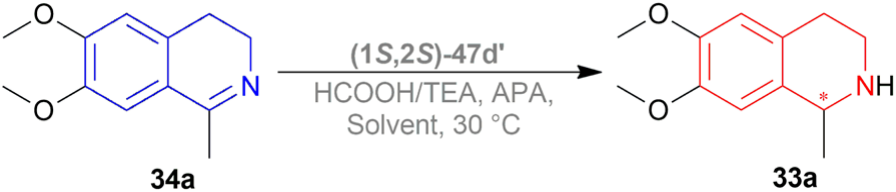
ATH of 1-methyl-6,7-dimethoxy-DHIQ.

A decrease of ee is seen in ATH of 1-methyl-6,7-dimethoxy-DHIQ 34a (Scheme 42) catalyzed by (1*S*,2*S*)-47d′ as the reaction time increases, and sometimes producing the other enantiomeric product. In ACN with APA, ee changed from 14% to 45% of (*S*)-1-methyl-6,7-dimethoxy-THIQ (*S*)-33a; without APA, ee changed from 77% to 58% of (*R*)-1-methyl-6,7-dimethoxy-THIQ (*R*)-33a. Similarly, in IPA with APA, ee changed from 43% of (*R*)-33a to 56% of (*S*)-33a; without APA, ee changed from 80% to 74% of (*R*)-33a.

So, (1*S*,2*S*)-47d′ catalyst gave rise to (*S*)-configured products with APA, and (*R*)-configured products without APA.

#### 2.2.24 Dual stereocontrol for AH of DHIQs by changing the mol% of *N*-bromosuccinimide

**Scheme 43:**
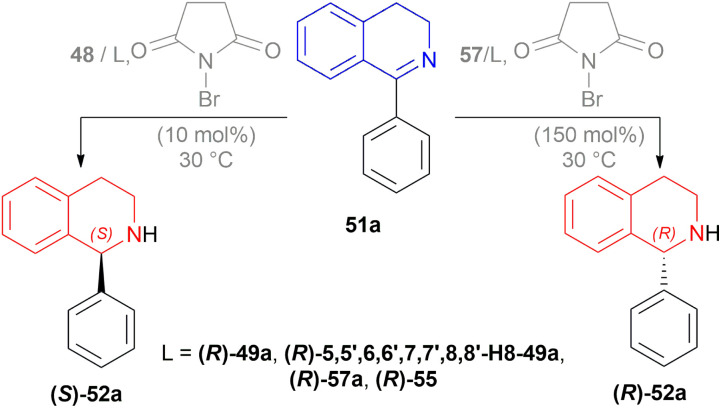
AH of 1-phenyl-DHIQs with dual stereo-control.

Instead of using two chiral reagents or two ligands of opposite configuration to get both enantiomers of chiral THIQs, Ji *et al.* explored dual enantioselective hydrogenation of 1-aryl-DHIQs with a single chiral catalyst.^60^*N*-BromoSuccinimide (NBS) elevated the valence state of iridium (1 to 3) which improved the catalytic performance or reacted with the substrate as an oxidant. This enabled dual stereo-control (Scheme 43) in iridium-catalysed AH of 1-aryl-DHIQs 51 by changing the amount of NBS.

1-phenyl-DHIQ 51a substrate with 48 – (*R*)-49a catalyst was evaluated. (*S*)-1-phenyl-THIQ (*S*)-52a was obtained in 82% ee (and full conversion) with 10 mol% NBS in 1,2-dichloroethane (DCE). Other ligands *e.g.*, (*R*)-5,5′,6,6′,7,7′,8,8′-H8-49a, (*R*)-57a, (*R*)-55 and other solvents *e.g.*, toluene, THF, or MeOH produced ee in the range of 63–79%. The ee increased to 86% when the reaction temperature was lowered to 0 °C.

The ee decreased to 55 and 13% when NBS was increased to 50 and 75 mol%. Increasing NBS to 90, 100, 120, and 150 mol% even produced (*R*)-1-phenyl-THIQ (*R*)-52a with increasing ee of 16–91%.

**Scheme 44:**
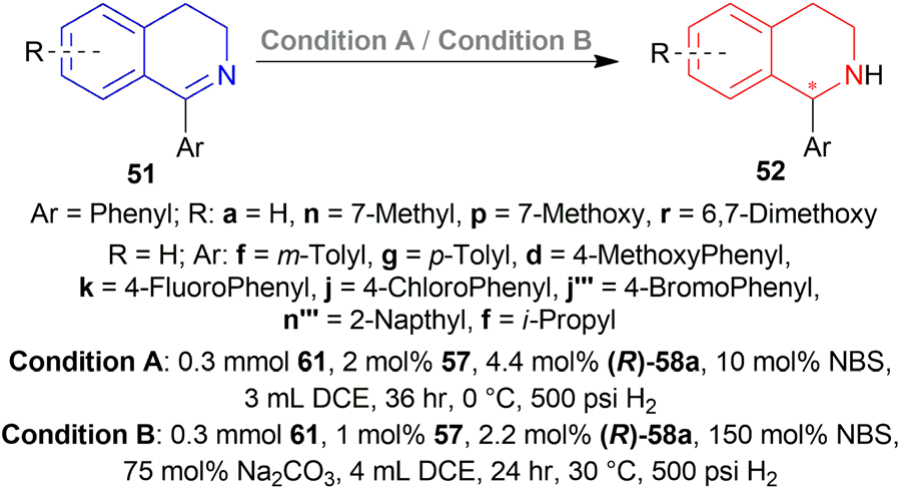
AH of 1-aryl-DHIQs with dual stereo-control.

Then 2 conditions were set, condition A and condition B (Scheme 44). For condition A, high ee was not dependent on the electronic properties of the substrates. Quite the opposite happened for condition B. The electronic properties of the substituted group of the benzene ring in the isoquinoline core affected the ee. Electronically deficient groups on 1-aryl position containing 51k, 51j, 51j′′′ had high ee of 95%, 94%, and 93% respectively. Electron-donating groups on 1-aryl position containing 51f, 51g, and 51d had low ee of 89%, 83%, and 66%. The methoxy group on the 1-aryl position containing 51d had a low ee of 66%. *i*-Propyl groups on 1-aryl position containing 51f had low ee of 66%.

**Scheme 45:**
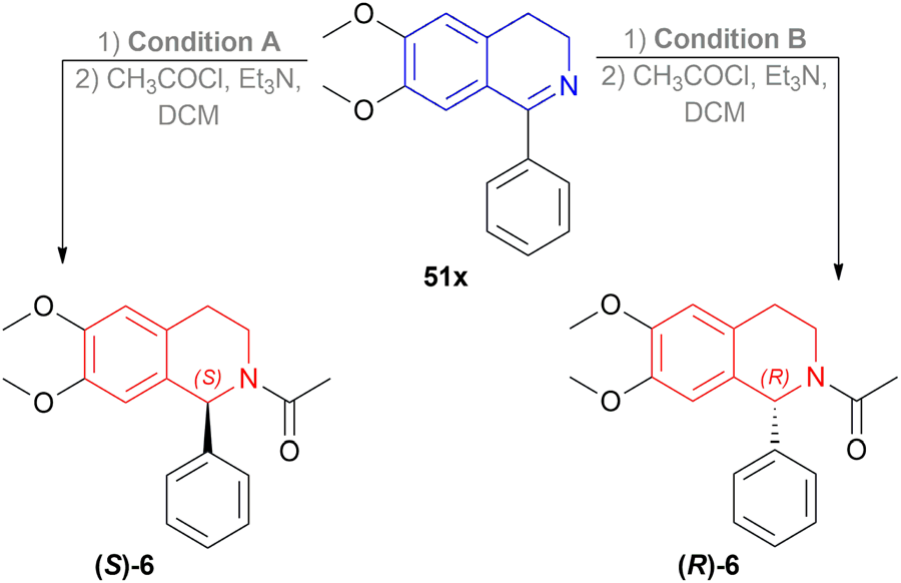
Synthesis of an AMPA receptor antagonist with dual stereo-control.

6, a biologically active compound, was prepared under the above standard conditions of the dual enantioselective hydrogenation of 51x (Scheme 45). condition A followed by acylation with acyl chloride, TEA, and DCM yielded 97% (*S*)-6 having 81% ee. While condition B with the same acylation process produced (*R*)-6 (98% yield, 91% ee) which is a potent non-competitive AMPA receptor antagonist.

#### 2.2.25 Josiphos-type binaphane ligands for iridium-catalyzed AH of 1-aryl-DHIQs

**Fig. 25:**
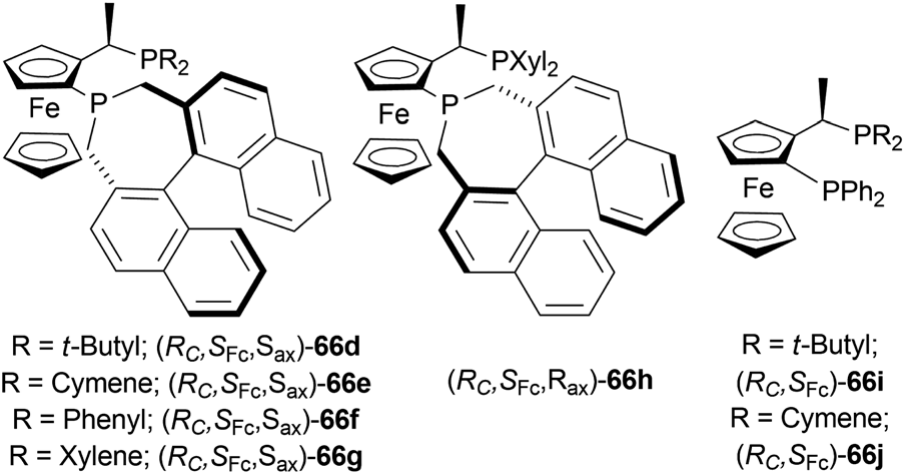
Josiphos-type binaphane ligands.

**Scheme 46:**
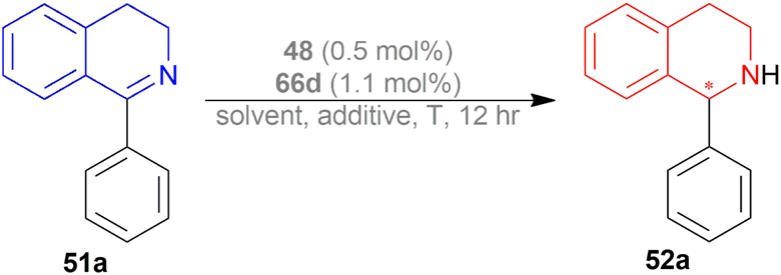
Screening AH of 1-phenyl-DHIQ with Josiphos-type binaphane ligands.

Nie *et al.* evaluated the ligands 66d–66j (Fig. 25) for asymmetric reduction of 1-phenyl-DHIQ 51a with 48 (Fig. 8), I_2,_ and TFA in toluene at 30 °C (Scheme 46).^61^66d–66h produced >99% yield. Among them, 66d had the highest 81% ee. 66i and 66j gave very poor results (65% and 60% yield; 35% and 21% ee respectively). So, 66d was chosen for the following experiments.

With I_2_ and TFA as additives, and THF as the solvent, 66d had >99% yield and 84% ee. No additive, I_2_, and TFA separately did not increase ee that much. Though 40% aq. solution of HBr was able to produce >99% ee. For this experiment, 40% aq. solution of HBr solution activated the substrate through HBr, and improved the catalytic activity by a six-membered cyclic transition state, forming salts between the substrate and HBr. Then increasing the temperature to 50 °C while reducing catalyst loading from 0.5 mol% to 0.1 mol% and 0.02 mol% decreased both yield and % ee.

**Scheme 47:**
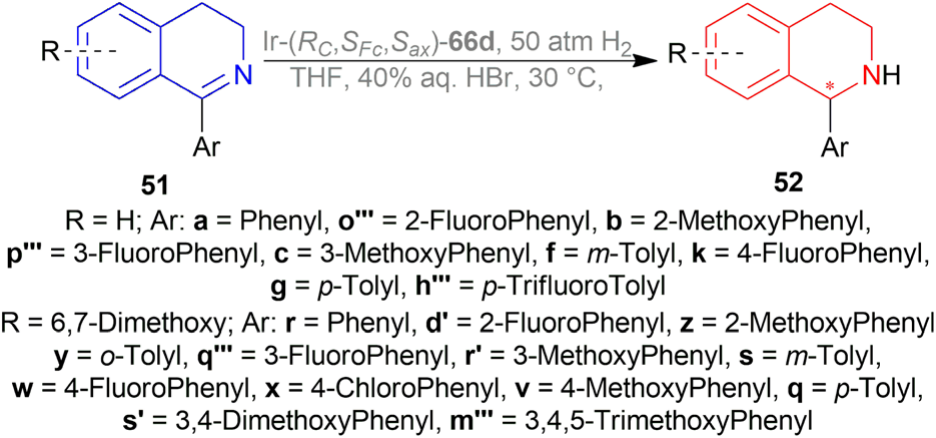
AH of 1-aryl-DHIQ with Josiphos-type binaphane ligands.

Then 51a–51c, 51f, 51g, 51k, 51q–51s, 51v–51z, 51d′, 51r′, 51s′, 51h′′′, 51m′′′, 51o′′′–51q′′′ were reduced under the above-mentioned conditions by AH (Scheme 47). All of these produced excellent results (94–99% yield, 85–99% ee). Some products such as 52b, 52c, 52f, 52g, 52k, 52h′′′, 52o′′′, and 52p′′′ had similar ee despite the difference in substituent position and electronegativity on the 1-phenyl ring.

For some products, increased ee were observed because of the 6,7-dimethoxy substitution in the THIQ core *e.g.*, 52q, 52v–52z, 52d′, 52r′. 52s′ and 52m′′′ having more than one methoxy substitution on the 1-phenyl ring caused decreased ee (Scheme 47).

52a, 52w, 52s′, and 52m′′′ were intermediates of biological active compounds, such as solifenacin, TRPM8 channel receptor antagonist IV, (*S*)-cryptostyline II, and (*S*)-cryptostyline III. And (*R*)-52h′′′ and (*R*)-52x can be obtained with the ligand 66d enantiomer, which were the intermediates of the TRPM8 channel receptor antagonist V and AMPA receptor antagonist I.

#### 2.2.26 Synthesis method of (*S*)-1-phenyl-THIQ

**Scheme 48:**
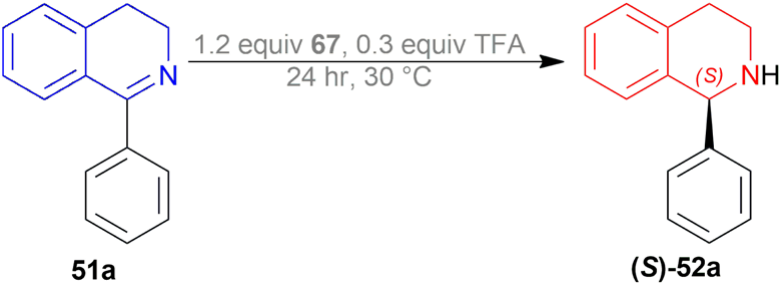
Reduction of 1-phenyl-DHIQ.

**Fig. 26:**
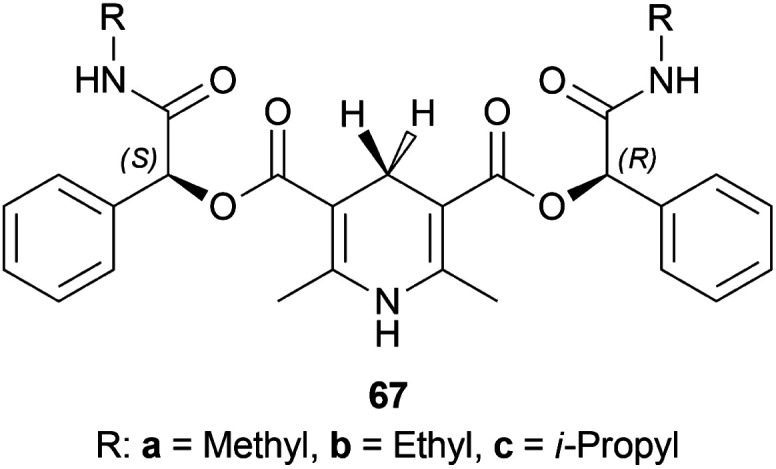
Chiral catalysts for the reduction of 1-phenyl-DHIQ.

Li *et al.* patented a method for synthesizing (*S*)-1-phenyl-THIQ (*S*)-52a (Scheme 48).^62^ Among the 9 examples described in this patent, example no. 1, 7, and 8 had the optimum yield, purity, and ee for chiral catalysts 67a–67c (Fig. 26) respectively.

The substrate 51a, catalyst 67, and TFA were dissolved in a ratio of 1 : 1.2 : 0.3 in 200 mL of toluene. The mixture was vigorously stirred for 24 h at 30 °C; adding water, quenched, extracted with ethyl acetate, concentrating the organic phase, purifying by recrystallization of the white crude product, and compound (*S*)-52a was obtained with the yield of 89, 85, and 75% and ee of 99.6, 99, and 98% for chiral catalysts 67a–67c respectively (Scheme 48).

#### 2.2.27 Refining method of solifenacin intermediate

**Fig. 27:**
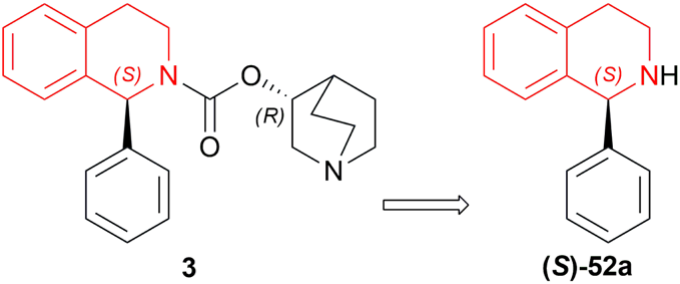
Solifenacin and its intermediate.

**Scheme 49:**
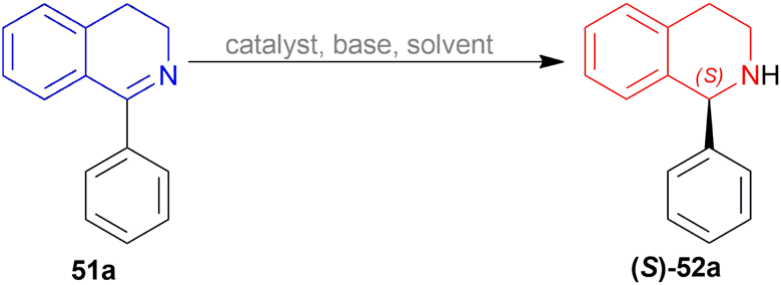
Reduction into solifenacin intermediate.

Solifenacin 3 has two chiral intermediates. Between them, Li *et al.* developed methods to synthesize and purify (*S*)-52a (Fig. 27) (Scheme 49).^63^

Among the 10 examples described in this patent, example 1 provided the most refined (*S*)-52a and the least impurity. In a high-pressure kettle, 1000 g of 51a dissolved in 10 L of ethanol is degassed completely under continuous argon introduction for 1 h 5 g of (*S*)-DIOP RuCl_2_ (*R*)-P-Me-BIMAH with 120 g potassium *tert*-butoxide was added to it and then hydrogen is replaced with the argon. The reaction was stirred at 25–35 °C and after completion, concentrated under reduced pressure to get 96% pure crude (*S*)-52a.

200 g of this crude was added to 1 L solution of a 5 : 1 mix of toluene and chlorobenzene, heated at 60 °C to dissolve, cooled to 5 °C, crystallized for 1 h, dried under reduced pressure to get 95% yield which was 99.8% pure with 0.02% maximum single purity.

Example 2–4 differed in the refinement of the crude, the second step, from example 1. When 1 L toluene was used in example 2, 90% yield with 99.3% purity and 0.07% maximum single impurity was found. 1 L chlorobenzene was used in example 3 where 94% yield with 99.6% purity and 0.06% maximum single impurity was found. In example 4, 1 L xylene caused a 90% yield with 99.2% purity and 0.06% maximum single impurity.

Example 5–6 differed in the refinement of the crude, the second step, from example 4. 40 °C temperature and 3 L xylene was used to dissolve the crude that yielded 82% with 99.6% purity and 0.04% maximum single purity in example 5. In example 6, 0.6 L xylene and 75 °C temperature dissolved the crude which caused 95% yield with 99.2% purity and 0.06% maximum single impurity.

Example 7–10 differed in the refinement of the crude, the second step, from example 1. 1 L solution of a 9 : 1 mix of toluene and chlorobenzene was used in example 7 where 89% yield with 99.5% purity and 0.04% maximum single impurity was found. In example 8, 1 L solution of a 5 : 1 mix of toluene and xylene caused 91% yield with 98.6% purity and 0.03% maximum single impurity. When 1 L solution of a 5 : 1 mix of xylene and chlorobenzene was used in example 9, 91% yield with 98.6% purity and 0.03% maximum single impurity was found. 1 L solution of a 5 : 1 mix of toluene and chlorobenzene and a crystallization temperature of 0 °C was used in example 10 where 96% yield with 98.6% purity and 0.08% maximum single impurity was found.

The only comparative example differed in the refinement of the crude, the second step, from example 1. 1 L THF was used here where 72% yield with 98.3% purity and 0.4% maximum single impurity was found (Scheme 49).

### 2.3 Examples of enantioselective reduction of 1-substituted-DHIQs possessing chiral auxiliary at the imine nitrogen by achiral metallic hydride reducing agents

#### 2.3.1 Asymmetric synthesis of fumarizine

**Scheme 50:**
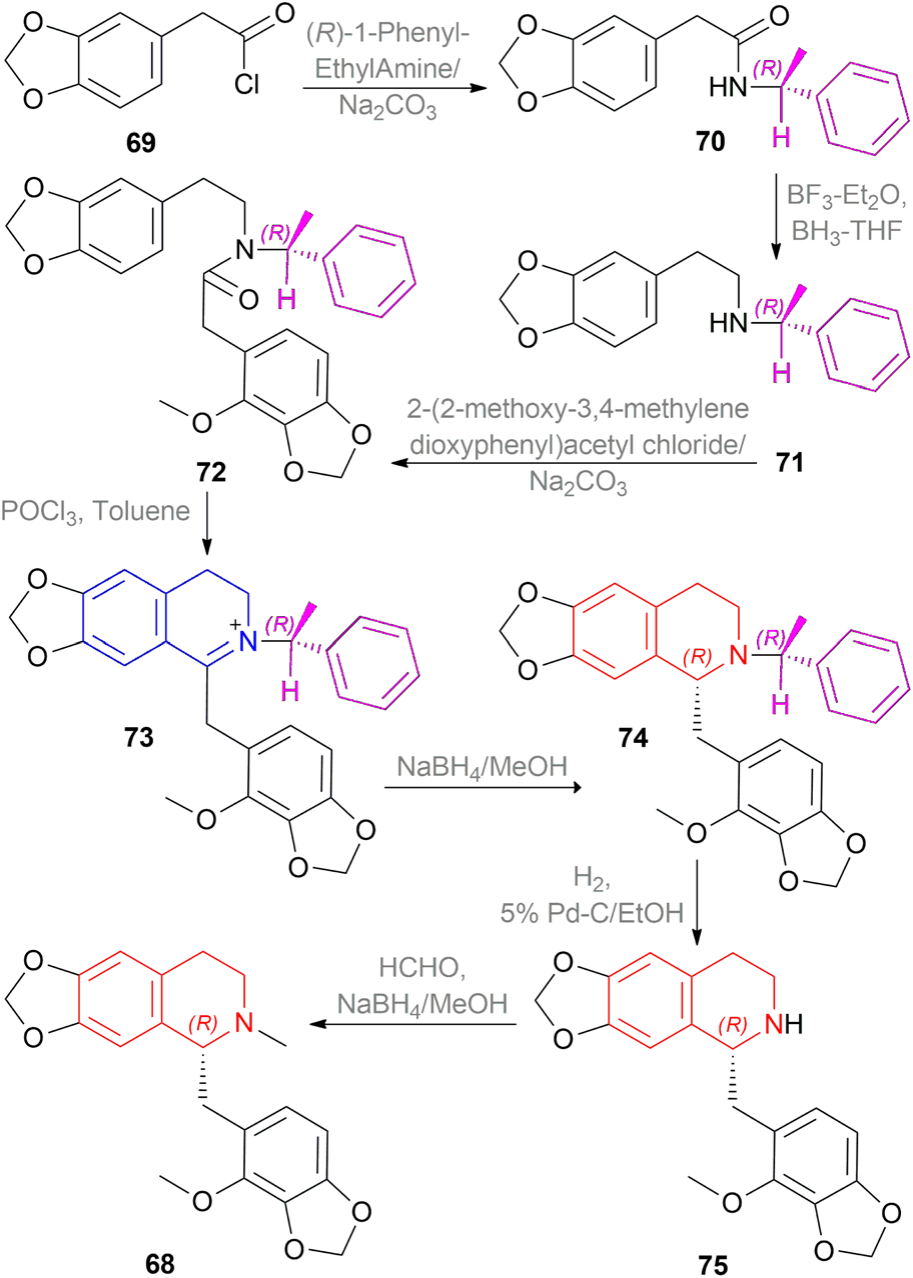
Asymmetric synthesis of fumarizine.

Kunitomo *et al.* used Polniaszek and McKee's method^64^ of chiral auxiliary to enantioselectively synthesize fumarizine 68 (Scheme 50).^65^

2-(3,4-methylenedioxyphenyl)acetyl chloride 69 was reacted with the chiral auxiliary (*R*)-1-phenylethylamine to produce *N*-[(*R*)-1-phenylethyl]-2-(3,4-methylenedioxyphenyl)acetamide 70. In this step, the chiral auxiliary was introduced into the substrate. Then 70 is reduced to *N*-[(*R*)-1-phenylethyl]-2-(3,4-methylenedioxyphenyl)ethylamine 71 with BH_3_-THF. After that, the Schotten–Baumann reaction of 71 and 2-(2-methoxy-3,4-methylenedioxyphenyl)acetyl chloride afforded *N*-[(*R*)-1-phenylethyl]-*N*-[2-(3,4-methylenedioxyphenylethyl)]-2-(2-methoxy-3,4-methylenedioxy-phenyl)acetamide 72.

Then, the Bischler–Napieralski reaction of 72 with POCl_3_ in toluene produced 1-(2-methoxy-3,4-methylenedioxybenzyl)-2-[(*R*)-1-phenylethyl]-6,7-methylenedioxy-3,4-dihydro-isoquinolin-2-ium 73 which was asymmetrically reduced with NaBH_4_ in MeOH to afford (*R*)-1-(2-methoxy-3,4-methylenedioxybenzyl)-2-[(*R*)-1-phenylethyl]-6,7-methylene-dioxy-THIQ 74. The chiral auxiliary was then removed by catalytic hydrogenation of 74 over Pd–C in ethanol to give rise (*R*)-1-(2-methoxy-3,4-methylenedioxybenzyl)-6,7-methylenedioxy-THIQ 75 having a total yield of 30% from 71. Treating 75 with HCHO and NaBH_4_ produced (*R*)-1-(2-methoxy-3,4-methylenedioxybenzyl)-2-methyl-6,7-methylenedioxy-THIQ or fumarizine 68.

So, (*R*)-configurated chiral auxiliary afforded (*R*)-configurated product.

#### 2.3.2 Asymmetric synthesis of (*R*)-noranicanine

**Scheme 51:**
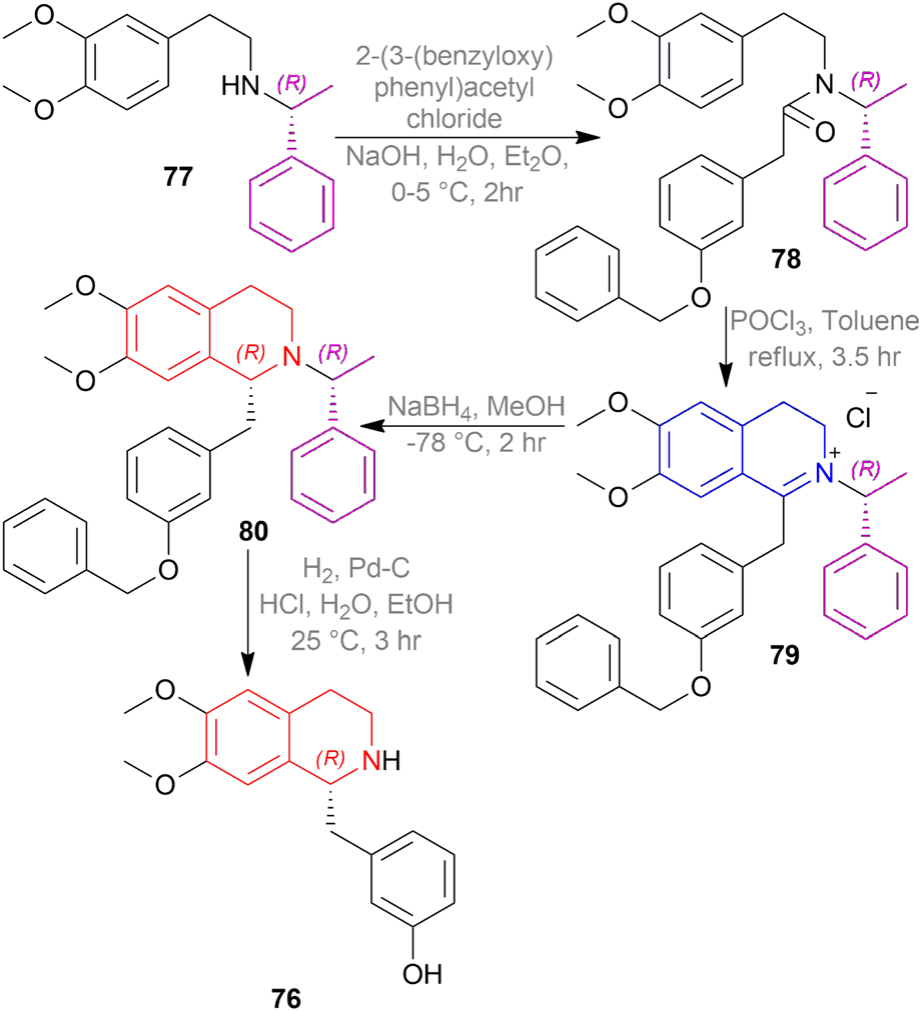
Asymmetric synthesis of (*R*)-noranicanine.

Kunitomo *et al.* used chiral auxiliary to synthesize (*R*)-noranicanine 76 (Scheme 51).^66^

At first, (*R*)-*N*-(2-(3,4-dimethoxyphenyl)ethyl)-1-phenyl-ethylamine 77 was reacted with 2-(3-(benzyloxy)phenyl)-acetyl chloride to produce (*R*)-2-(3-(benzyloxy)phenyl)-*N*-(2-(3,4-dimethoxyphenyl)ethyl)-*N*-(1-phenyl-ethyl)acetamide 78.

Then, the Bischler–Napieralski reaction of 78 with POCl_3_ in toluene produced (*R*)-1-(3-(benzyloxy)benzyl)-2-(1-phenyl-ethyl)-6,7-dimethoxy-3,4-dihydroisoquinolin-2-ium chloride 79. In this example, (*R*)-1-phenylethyl is the chiral auxiliary. 79 was asymmetrically reduced with NaBH_4_ in MeOH to afford (*R*)-1-(3-(benzyloxy)benzyl)-6,7-dimethoxy-2-((*R*)-1-phenyl-ethyl)-THIQ 80. At last, the chiral auxiliary was removed with H_2_/Pd–C to get (*R*)-1-(3-hydroxy)benzyl-6,7-dimethoxy-THIQ or (*R*)-noranicanine 76.

So, (*R*)-configurated chiral auxiliary afforded (*R*)-configurated product.

#### 2.3.3 Asymmetric synthesis of (*S*)- and (*R*)-salsolidines and (*R*)-cryptostyline

**Scheme 52:**
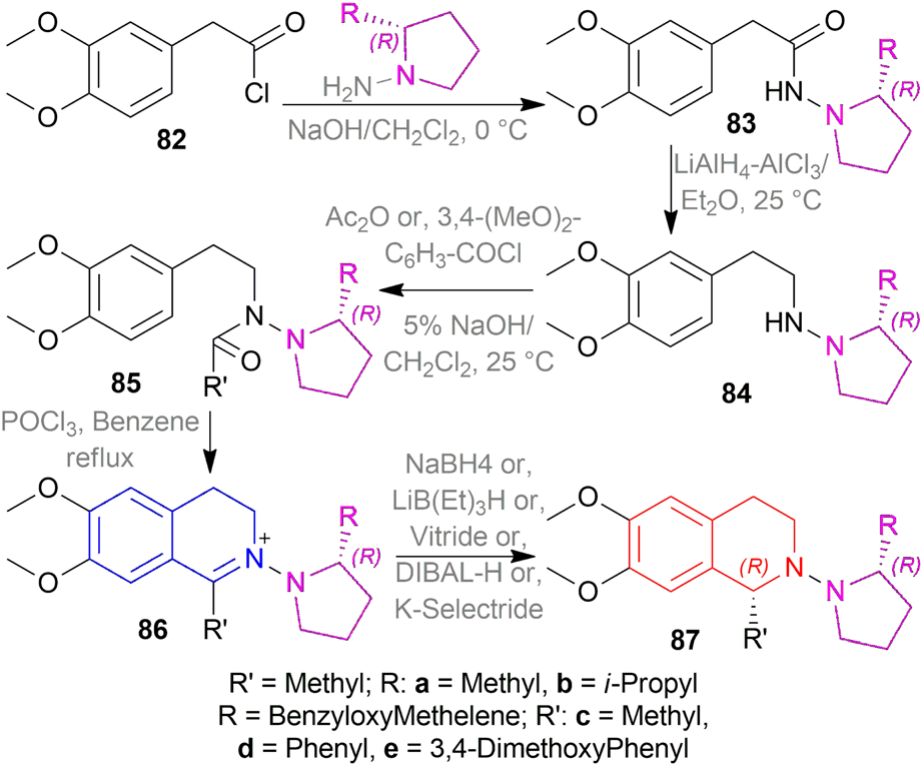
Asymmetric synthesis of (*S*)- and (*R*)-salsolidines and (*R*)-cryptostyline.

**Scheme 53:**
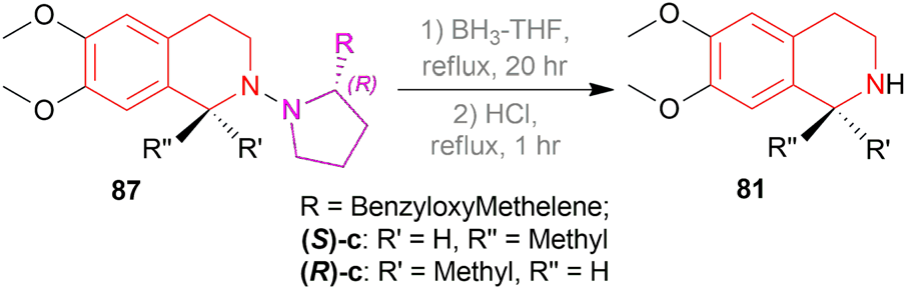
Asymmetric synthesis of (*S*)- and (*R*)-salsolidines.

**Scheme 54:**
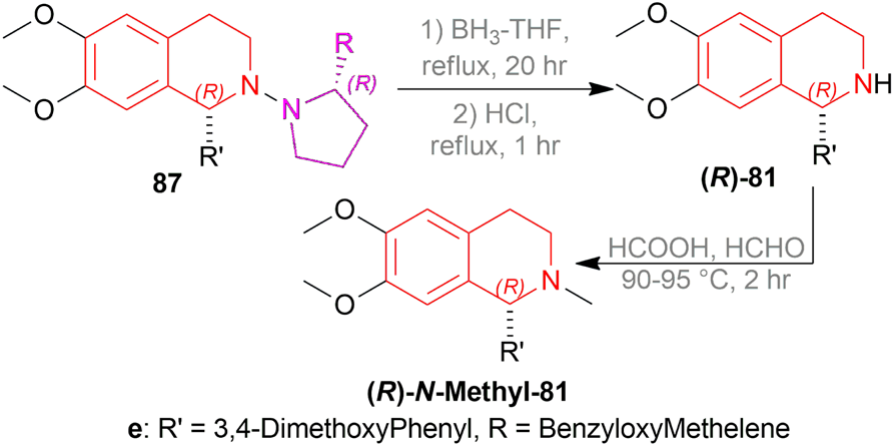
Asymmetric synthesis of (*R*)-cryptostyline.

Suzuki *et al.* synthesized (*S*)- and (*R*)-salsolidines 81c and (*R*)-cryptostyline (*R*)-*N*-methyl-81e with a chiral auxiliary (Schemes 52–54).^67^

2-(3,4-Dimethoxyphenyl)acetyl chloride 82 was reacted with (*R*)-2-substituted-pyrrolidin-1-amines to produce (*R*)-2-(3,4-dimethoxyphenyl)-*N*-(2-substituted-pyrrolidin-1-yl)acetamides 83. Then, 83 was reduced to (*R*)-*N*-(2-(3,4-dimethoxy-phenyl)ethyl)-2-substituted-pyrrolidin-1-amines 84 which was reacted with Ac_2_O or, 3,4-dimethoxybenzoyl chloride to afford (*R*)-*N*-(3,4-dimethoxyphenethyl)-*N*-(2-substituted-pyrrolidin-1-yl)amides 85.

Then, the Bischler–Napieralski reaction of 85 with POCl_3_ in benzene produced (*R*)-6,7-dimethoxy-1-substituted-2-(2-substituted-pyrrolidin-1-yl)-3,4-dihydroisoquinolin-2-iums 86. Here, 2-substituted-pyrrolidin-1-yl is the chiral auxiliary. 86 was asymmetrically reduced with various metal hydride reagents to afford (*R*)-6,7-dimethoxy-1-substituted-2-((*R*)-2-substituted-pyrrolidin-1-yl)-THIQs 100.

NaBH_4_ in MeOH at −50 °C yielded 73% (*R*)-87a (84% ee), 71% (*R*)-87b (86% ee), 75% (*R*)-87c (94% ee), and 70% (*R*)-87d (90% ee) respectively. NaBH_4_ in MeOH at −10, −50, and −90 °C yielded 88%, 84%, 81% (*R*)-87c (90%, 92%, 94% ee) respectively. Again, LiB(Et)_3_H, vitride, DIBAL-H, and K-selectride in THF at −50 °C yielded 68%, 63%, 42%, and 44% (*R*)-87c (92%, 92%, 92%, and 96% ee) respectively.

So, (*R*)-configurated chiral auxiliary afforded (*R*)-configurated product.

Then, the chiral auxiliary was removed by refluxing (*S*)-87c, (*R*)-87c, and (*R*)-87e in BH_3_-THF for 20 h and in HCl for 1 h producing 77% (*S*)-6,7-dimethoxy-1-methyl-THIQ or (*S*)-salsolidine (*S*)-87c, 77% (*R*)-6,7-dimethoxy-1-methyl-THIQ or (*R*)-salsolidine (*R*)-81c (Scheme 53), and 71% (*R*)-94e; which was *N*-methylated with HCOOH, HCHO to afford 73% (*R*)-6,7-dimethoxy-1-(3,4-dimethoxyphenyl)-2-methyl-THIQ or (*R*)-cryptostyline II (*R*)-*N*-Methyl-94e (Scheme 54).

#### 2.3.4 Enantioselective synthesis of (*R*)- and (*S*)-cryptostyline II

**Scheme 55:**
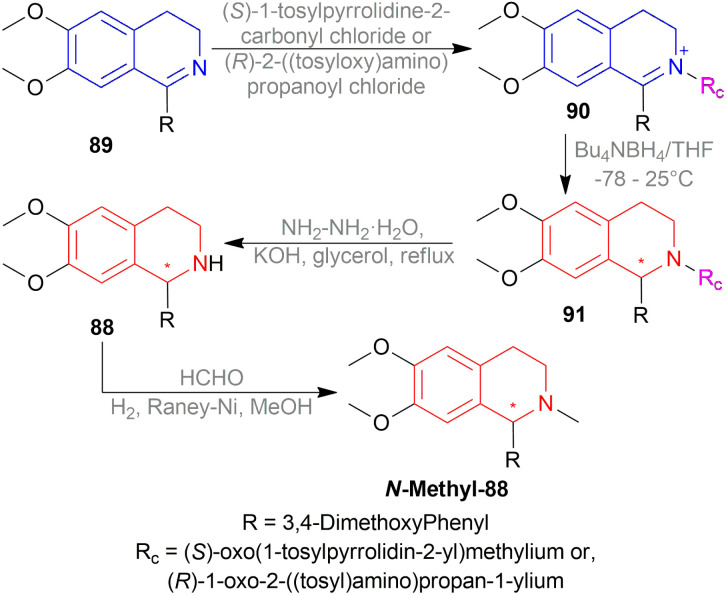
Enantioselective synthesis of (*R*)- and (*S*)-cryptostyline II.

Czarnocki and Mieczkowski synthesized (*R*)- and (*S*)-cryptostyline II *N*-Methyl-88 using chiral auxiliary (Scheme 55).^68^

1-(3,4-Dimethoxyphenyl)-6,7-dimethoxy-DHIQ 89 were reacted with (*S*)-1-tosylpyrrolidine-2-carbonyl chloride or (*R*)-2-((tosyloxy)amino)propanoyl chloride to introduce the chiral auxiliary and produced 1-(3,4-dimethoxyphenyl)-2-substituted-6,7-dimethoxy-3,4-dihydroisoquinolin-2-ium 90. Here, (*S*)-oxo(1-tosylpyrrolidin-2-yl)methylium and (R)-1-oxo-2-((tosyloxy)amino)propan-1-ylium were the chiral auxiliary respectively. 90 were reduced to (*S*)- or (*R*)-1-(3,4-dimethoxy-phenyl)-2-substituted-6,7-dimethoxy-THIQ 91. Then the chiral auxiliaries will be removed to get (*S*)- or, (*R*)-1-(3,4-dimethoxy-phenyl)-6,7-dimethoxy-THIQ 88 which were *N*-methylated to get (*S*)- or (*R*)-1-(3,4-dimethoxy-phenyl)-2-methyl-6,7-dimethoxy-THIQ or, (*S*)- or (*R*)-cryptostyline II *N*-methyl-88.

When (*S*)-1-tosylpyrrolidine-2-carbonyl chloride was used, 94% (*S*)-*N*-methyl-88 (66% ee) was found. But when (*R*)-2-((tosyl)-amino)propanoyl chloride was used, 87% yield and 59% ee of (*R*)-*N*-methyl-88 was seen.

So, (*S*)-configurated chiral auxiliary afforded (*S*)-configurated product and (*R*)-configurated chiral auxiliary afforded (*R*)-configurated product in moderate ee.

#### 2.3.5 Asymmetric synthesis of dehassiline

**Scheme 56:**
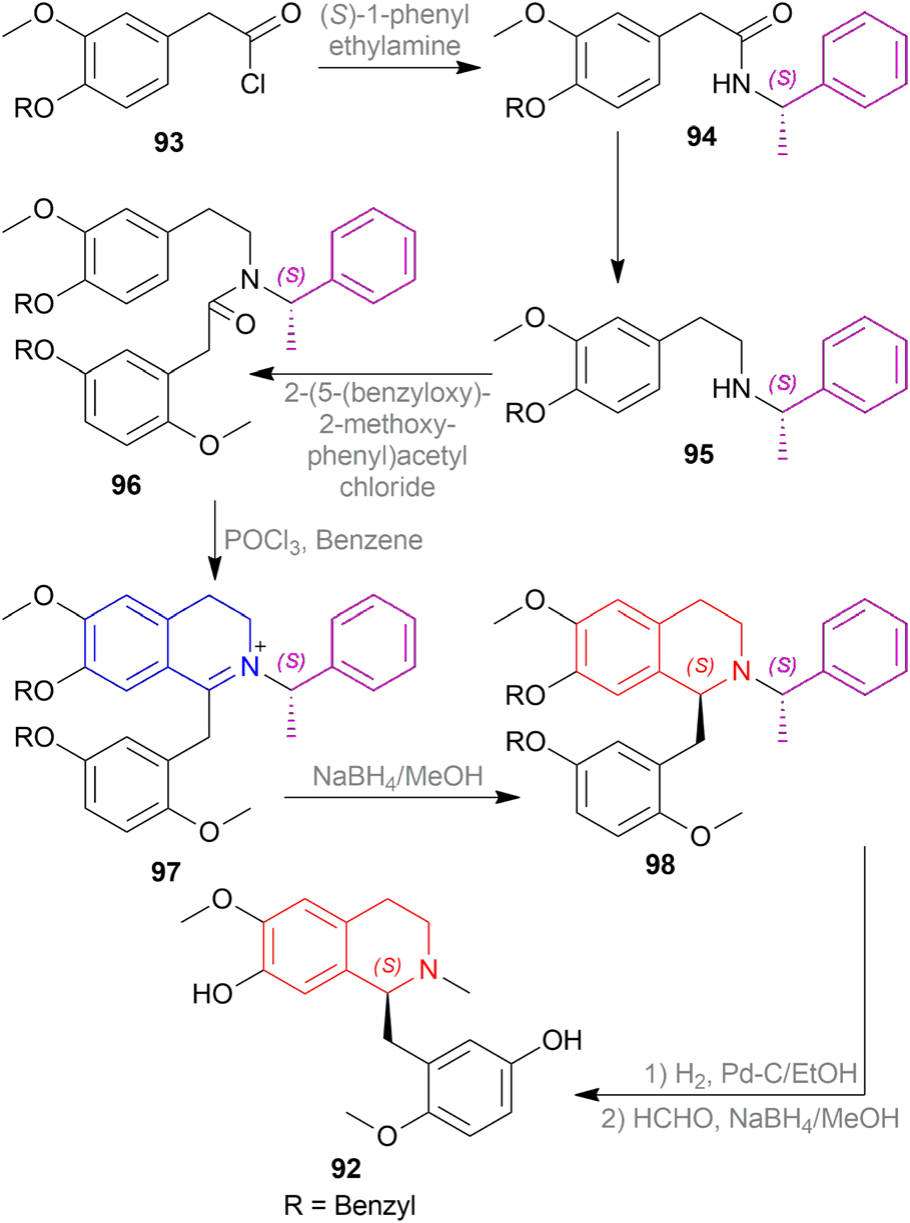
Asymmetric synthesis of dehassiline.

Kunitomo *et al.* afforded dehassaline 92 after using a chiral auxiliary for asymmetric synthesis (Scheme 56).^69^

2-(4-(Benzyloxy)-3-methoxyphenyl)acetyl chloride 93 was reacted with (*S*)-1-phenylethylamine to produce 2-(4-(benzyloxy)-3-methoxyphenyl)-*N*-((*S*)-1-phenylethyl)-acetamide 94. Then, 94 was reduced to *N*-(2-(4-(benzyloxy)-3-methoxyphenyl)ethyl)-(*S*)-1-phenylethylamine 95 which was reacted with 2-(5-(benzyloxy)-2-methoxyphenyl)acetyl chloride to afford 2-(5-(benzyloxy)-2-methoxyphenyl)-*N*-(2-(4-(benzyloxy)-3-methoxyphenyl)ethyl)-*N*-((S)-1-phenylethyl)-acetamide 96.

Then, the Bischler–Napieralski reaction of 96 with POCl_3_ in benzene produced 6-methoxy-7-(benzyloxy)-1-(5-(benzyloxy)-2-methoxybenzyl)-2-((*S*)-1-phenylethyl)-3,4-dihydro-isoquinolin-2-ium 97. Here, (*S*)-1-phenylethyl is the chiral auxiliary. 97 was asymmetrically reduced with NaBH_4_ in MeOH at −78 °C to afford (*S*)-7-(benzyloxy)-1-(5-(benzyloxy)-2-methoxybenzyl)-6-methoxy-2-((*S*)-1-phenylethyl)-THIQ 98. Then the chiral auxiliary was removed, *N*-methylated, and debenzylated to afford (*S*)-1-(5-hydroxy-2-methoxybenzyl)-7-hydroxy-6-methoxy-2-methyl-THIQ or dehassiline 92.

So, (*S*)-configurated chiral auxiliary afforded (*S*)-configurated product.

#### 2.3.6 Enantioselective syntheses of dopaminergic (*R*)- and (*S*)-benzyltetrahydroisoquinolines

**Scheme 57:**
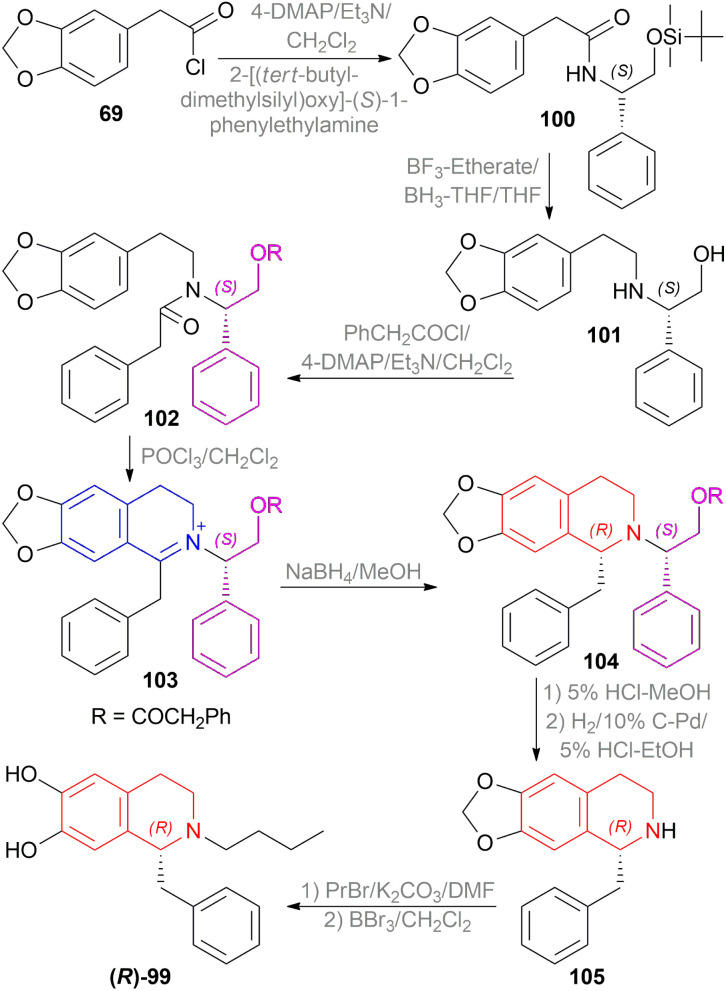
Enantioselective synthesis of (*S*)-1-benzyl-2-propyl-6,7-dihydroxy-THIQ.

Cabedo *et al.* synthesized (*R*)- and (*S*)-1-benzyl-2-propyl-6,7-dihydroxy-THIQ 99 with chiral auxiliary (Scheme 57).^70^

2-(3,4-Methylenedioxyphenyl)acetyl chloride 69 was reacted with 2-[(*tert*-butyldimethylsilyl)oxy]-(*S*)-1-phenylethylamine to produce *N*-[2-*tert*-butyldimethylsilyl-(*S*)-1-phenylethoxy]-2-(3,4-methylenedioxyphenyl)acetamide 100 which was reduced to *N*-[2-(3,4-methylenedioxyphenyl)ethyl]-(*S*)-1-phenyl-ethanolamine 101. Then, 101 was added with phenylacetyl chloride to afford *N*-[(*S*)-1-phenyl-2-phenylacetylethoxy-*N*-2-(3,4-methylenedioxyphenyl)ethyl]-2-phenylacetamide 102.

Then, the Bischler–Napieralski reaction of 102 with POCl_3_ in DCM produced 1-benzyl-2-[(*S*)-1-phenyl-2-phenylacetylethoxy]-6,7-methylenedioxy-3,4-dihydroisoquinolin-2-ium 103 which was asymmetrically reduced with NaBH_4_ in MeOH to afford (*R*)-1-benzyl-2-[(*S*)-1-phenyl-2-phenylacetylethoxy]-6,7-methylene-dioxy-THIQ 104. The chiral auxiliary was removed in two steps using 5% HCl in MeOH and H_2_ over 10% C–Pd in 5% HCl–EtOH producing (*R*)-1-benzyl-6,7-methylenedioxy-THIQ 105. Lastly, 105 was *N*-propylated with 1-bromopropane and K_2_CO_3_ in DMF and the 6,7-methylenedioxy part was cleaved with boron tribromide in DCM to obtain (*R*)-1-benzyl-2-propyl-6,7-dihydroxy-THIQ (*R*)-99 in 69% yield.

In the above scheme, if 2-[(*tert*-butyldimethylsilyl)oxy]-(*R*)-1-phenylethylamine was used without changing any other reagents, the final product yielded 58% (*S*)-1-benzyl-2-propyl-6,7-dihydroxy-THIQ (*S*)-99.

So, (*S*)-configurated chiral auxiliary afforded (R)-configurated product.

### 2.4 Enantioselective reduction of 1-substituted-DHIQs by enzymatic catalysis

#### 2.4.1 Enantioselective reduction by imine reductase *Ao*IRED from *Amycolatopsis orientalis*

**Scheme 58:**
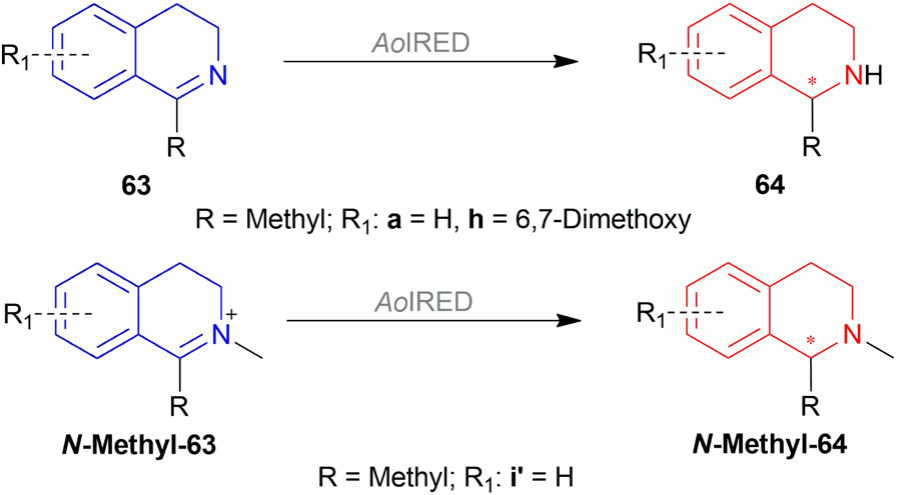
Enantioselective reduction by *Ao*IRED.

Aleku *et al.* reduced 63a, 63h, and *N*-methyl-63i′ by freshly purified imine reductase *Ao*IRED (UniProt: R4SNK4) from *Amycolatopsis orientalis* (Scheme 58).^71^ (*S*)-64a, (*S*)-64h and (*S*)-*N*-methyl-64i′ was found with 100%, 50%, and 40% conversion (81%, 79%, and 92% ee) respectively. Also, fresh lysate produced a similar 85% ee of (*S*)-64a.

But, 98% and >99% ee of (*R*)-64a was produced by AoIRED stored at 4 °C for 24 h and after 14 days respectively.

When bovine serum albumin (BSA) added to *Ao*IRED was aged for 24 h, 76% ee of (*S*)-64a was found.

*Ao*IRED derived from Y179A mutant of *Amycolatopsis orientalis* was the highest in catalytic efficiency for 63a.

#### 2.4.2 Enantioselective reduction by imine reductase *Sn*IRED from *Stackebrandtia nassauensis*

**Scheme 59:**
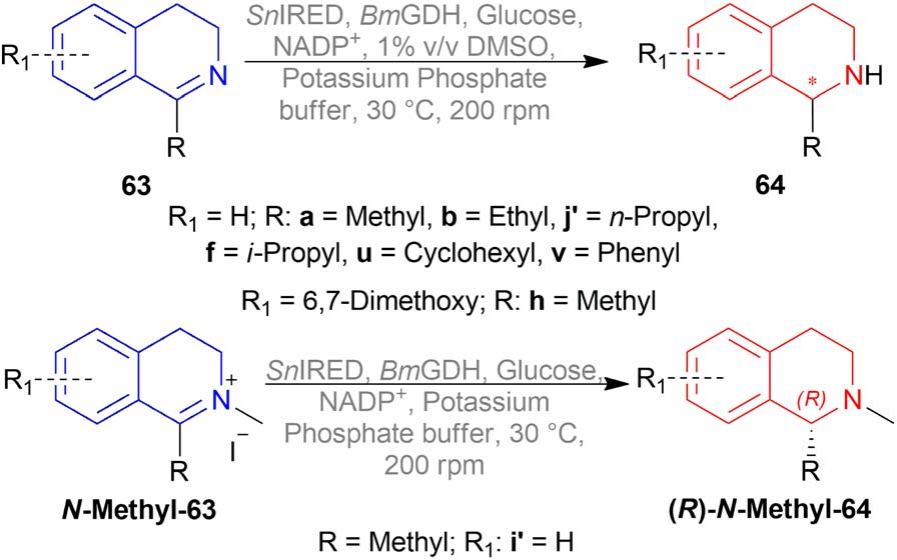
Enantioselective reduction by *Sn*IRED.

Li *et al.* screened several imine reductases (IREDs) such as *Sn*IRED from *Stackebrandtia nassauensis*, *Se*IRED from *S. espanaensis*, and *Ad*IRED from *A. decaplanina* to reduce 63a (Scheme 59).^72^ For highest specific activity and >99% ee of (*S*)-64a, *Sn*IRED was chosen.

Substrate concentration and % (v/v) of DMSO as co-solvent was also screened. 100 mM substrate with 5% DMSO had only 18% conversion even after 24 h of reaction. Then decreasing concentration, or % DMSO, or both lowered reaction time (0.5–12 h) with >99% conversion.

Then 63a, 63b, 63f, 63h, 63u, 63v, and 63j′ were reduced with *Sn*IRED, Glucose Dehydrogenase from *Bacillus megaterium* (*Bm*GDH), glucose, NADP^+^, 1% DMSO, potassium phosphate buffer (pH 7.0) while no DMSO was used for reducing *N*-methyl-63i′. 100 mM of 63a, 63b, and 63h were totally converted to (*S*)-64a, (*S*)-64b, and (*S*)-64h in 2–4 h in 72–81% yields and 93–99% ee. The other substrates were used in 5–50 mM concentration for 3–12 h to yield 73–81% of their respective products. (*S*)-64f and (*S*)-64v was produced in 98% and 51% ee, while (*R*)-64j′, (*R*)-64u, and (*R*)-*N*-methyl-64i′ was produced in 8%, 80%, and 87% ee respectively. So, size of 1-substitution and substrate binding mode was a critical factor in the stereoselectivity of *Sn*IRED.

#### 2.4.3 Enantioselective reduction by hindrance-tolerated IREDs

**Scheme 60:**
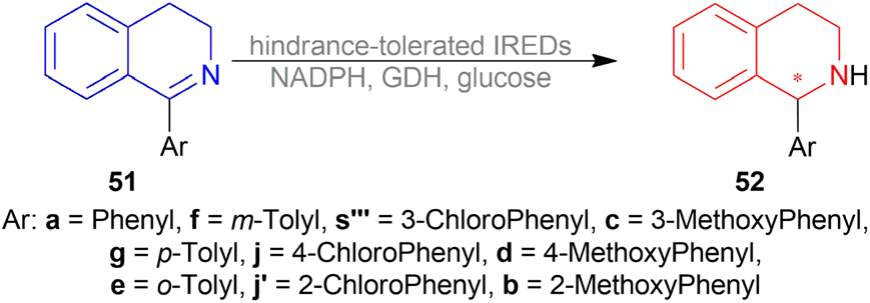
Enantioselective reduction by hindrance-tolerated IREDs.

Zhu *et al.* screened 88 novel IREDs to reduce 51a at 30 °C for 24 h with NADPH, glucose dehydrogenase (GDH) and glucose (Scheme 60).^73^ Among those IRED2, IRED8, IRED17, IRED19, IRED20, IRED32, and IRED45 had 99–100% conversion with 96 – >99% ee. *Meta*-substituted 51f, 51s′′′, 51c also had similar conversion of 99–100% and ee of 91 – >99% with these IREDs. Except IRED45, para-substituted 51g, 51j, 51d had good conversion of 99–100% and ee of >99% with these IREDs. But only IRED2 and IRED45 showed little conversion for *ortho*-substituted 51e, 51j′, 51b. IRED8, IRED17, IRED19, IRED20, IRED32 produced (*R*)-1-aryl-THIQs. IRED2 also had (*R*)-configured products except for (*S*)-52j′. In contrast, IRED45 had (*S*)-configured products except for (*R*)-52j′ and (*R*)-52b.

**Scheme 61:**
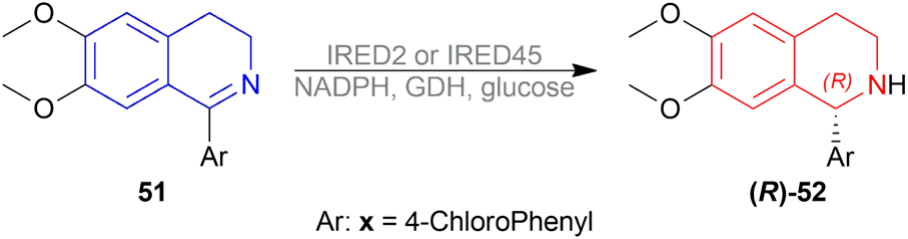
Enantioselective reduction by IRED2 or IRED45.

Then, IRED2 and IRED45 were selected for reducing 51x (Scheme 61). IRED2 produced 62% conversion with 99% ee of (*R*)-52x, and IRED45 produced 53% conversion with 92% ee of (*R*)-52x.

#### 2.4.4 Enantioselective reduction by two types of IREDs

**Scheme 62:**
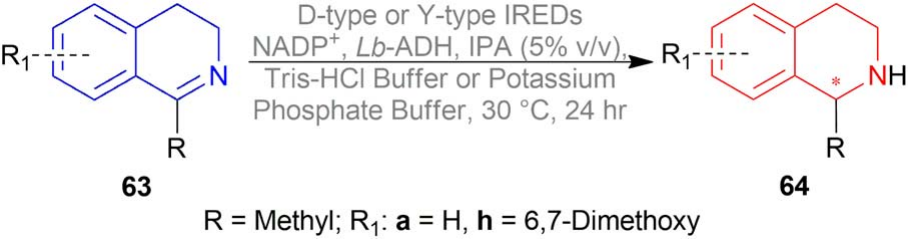
Enantioselective reduction by D-type and Y-type IREDs.

Velikogne *et al.* screened IRED-A-IRED-H (D-type) and IRED-I-IRED-N (Y-type) to reduce 63a and 63h with NADP^+^, alcohol dehydrogenase from *Lactobacillus brevis* (*Lb*-ADH), Tris–HCl buffer (pH 7.5, for IRED-A-IRED-E, IRED-I-IRED-M) or potassium phosphate buffer (pH 6.0, for IRED-F-IRED-H, IRED-N), IPA (5% v/v), 30 °C, 24 h (Scheme 62).^74^

Except IRED-B, all the D-type IREDs had conversion of 83 – >99% and 71 – >99% ee for 64a. But only IRED-D-IRED-G had low conversion of 15–49% for 64h. These IREDs mainly produced (*R*)-configured products except IRED-G. Among these, IRED-C (from *Micromonospora* sp. M42, UniProt: W7VJL8), IRED-D (*Mesorhizobium* sp. L2C089B000, UniProt: V7GV82), IRED-E (*Nocardiopsis alba*, UniProt: J7LAY5), and IRED-G (from *Streptomyces rimosus* ATCC 10970, UniProt: L8EIW6) had the highest conversion >99% and >99% ee for 64a.

All the Y-type IREDs had 59 – >99% conversion and 91 – >99% ee for (*S*)-64a. And except IRED-N, these had >99% conversion and >99% ee for (*S*)-64h. These IREDs produced mainly (*S*)-configured products. Among these, IRED-J (from *Kribbella flavida* DSM 17836, UniProt: D2PR38), IRED-L (from *Nocardia brasiliensis* ATCC 700358, K0F8R0), and IRED-M (from saccharothrix espanaensis ATCC 51144, UniProt: K0K4C6) had the highest conversion and % ee for both (*S*)-64a and (*S*)-64h.

#### 2.4.5 Enantioselective reduction by IRED45 and its mutant

**Scheme 63:**
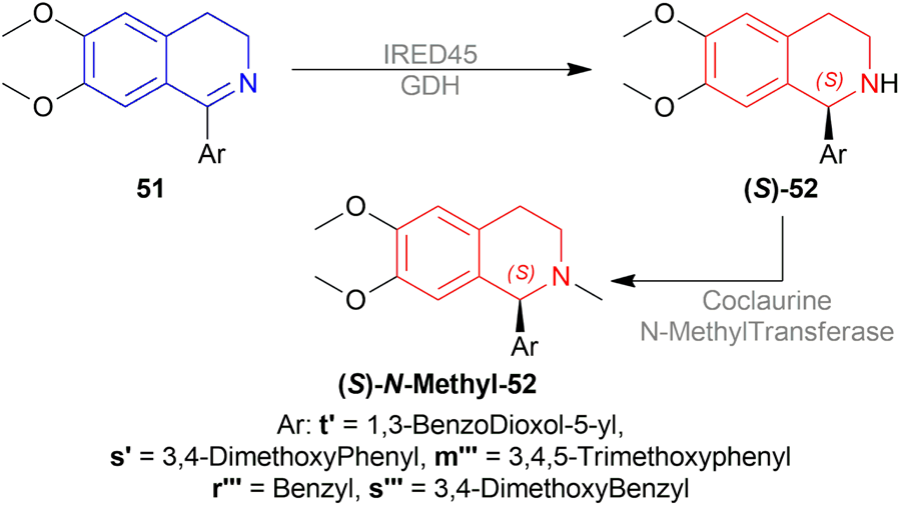
Enantioselective reduction by IRED45 and its mutant.

Yang *et al.* described reduction of 51t′, 51s′, 51m′′′, 51r′′′, 51s′′′ by IRED45 (as of Section 2.4.3) and its mutant at 30 °C for 24 h (Scheme 63).^75^ IRED45 was able to produce (*S*)-52t′, (*S*)-52r′′′, (*S*)-52s′′′ with full conversion and >99% ee. But it did not reduce 51s′, 51m′′′ which was bulkier than 51a. While the mutant W191F was not useful at all, it's another mutant F190M-W191F was similar in activity as IRED45. Another mutant F190L-W191F had full conversion and >99% ee for (*S*)-52t′, (*S*)-52m′′′, (*S*)-52r′′′, (S)-52s′′′ and 75% conversion and >99% ee for (*S*)-52s′.

Then *N*-methylation of (*S*)-52t′, (*S*)-52s′, (*S*)-52m′′′, (*S*)-52r′′′, and (S)-52s′′′ with coclaurine *N*-methyltransferase produced (*S*)-cryptostyline I, (*S*)-cryptostyline II, (*S*)-cryptostyline III, (*S*)-1-benzyl-6,7-dimethoxy-2-methyl-THIQ, and (S)-laudanosine with 100% conversion.

#### 2.4.6 Enantioselective reduction by D187 subgroup of IREDs

**Scheme 64:**
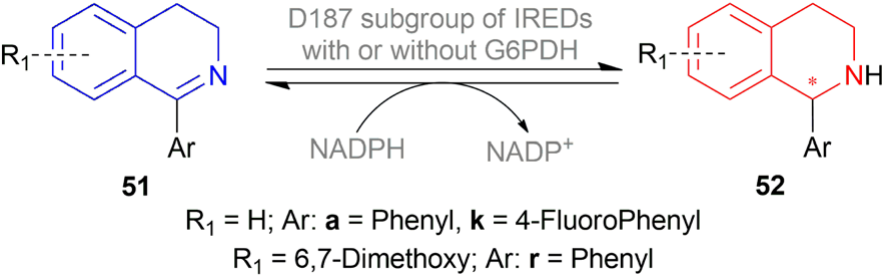
Enantioselective reduction by D187 subgroup of IREDs.

Cárdenas-Fernández *et al.* reduced 51a, 51k, and 51r with pQR2595, pQR2600, pQR2601, and pQR2612 which are four D187 subgroup of IREDs (Scheme 64).^76^

pQR2600 produced 93% ee of (*R*)-52a, 84% and 21% yield with and without glucose-6-phosphate dehydrogenase (G6PDH) respectively. Similarly, pQR2601 had shown >99% ee of (*R*)-52a, 98% and 23% yield with and without G6PDH respectively.

pQR2595 produced >99% ee of (*R*)-52k, 74% and 33% yield with and without G6PDH respectively. Similarly, pQR2612 had shown >99% ee of (*S*)-52r, 76% and 23% yield with and without G6PDH respectively.

## 3 Conclusion

Most of the isoquinoline alkaloids, a large family of natural products, are comprised of the 1-substituted-1,2,3,4-tetrahydroisoquinolines. Because of their (1-substituted-THIQs) diversified structure, innumerable biological activities, and a chiral center in their nucleus have made them fascinating targets for organic synthesis. Since these compounds contain a chiral carbon, a wide range of enantioselective synthetic methods have been reported in the last forty-one years.

The enantioselective reductions of 1-substituted-DHIQs, obtained by the Bischler–Napieralski reaction, to get the intended 1-substituted-1,2,3,4-tetrahydroisoquinoline in chiral form were accomplished by using chiral hydride reducing agents, by hydrogenation in the presence of a chiral catalyst, by enantioselective reduction of DHIQs possessing a chiral auxiliary at the imine nitrogen by achiral metallic hydride reducing agents, or by enzymatic catalysis. It has been found that using hydrogen gas and a very small quantity of chiral catalysts, asymmetric hydrogenation provides the most efficient way to synthesize enantio-enriched compounds. Therefore, there is more scope and potential for research concerning the remaining three methods.

No specific, general, or simple methods were found in this review article for the preparation of all types of isoquinoline alkaloids with high optical purity. Moreover, moderate to poor yields and stereoselectivity, inaccessibility, or high costs of starting materials and reagents are the limitations of these methods. Hence, the development of novel methods for finding 1-substituted-THIQs in optically active form can still be a subject of research.

## Author contributions

M. M. A. Asif: writing – original draft, visualization, writing – review & editing. S. R. Lisa: writing – original draft, writing – review & editing. N. Qais: supervision, writing – review & editing.

## Conflicts of interest

There are no conflicts to declare.

## Supplementary Material
